# Multi-population Genome-Wide Association Study Identifies Multiple Novel Loci associated with Asymptomatic Intracranial Large Artery Stenosis

**DOI:** 10.1101/2025.05.06.25327093

**Published:** 2025-05-07

**Authors:** Minghua Liu, Farid Khasiyev, Antonio Spagnolo-Allende, Danurys L Sanchez, Howard Andrews, Qiong Yang, Alexa Beiser, Ye Qiao, Jose Rafael Romero, Tatjana Rundek, Adam M Brickman, Jennifer J Manly, Mitchell SV Elkind, Sudha Seshadri, Christopher Chen, Oscar H Del Brutto, Saima Hilal, Bruce A Wasserman, Giuseppe Tosto, Myriam Fornage, Jose Gutierrez

**Affiliations:** 1 Department of Neurology, Vagelos College of Physicians and Surgeons, Columbia University, New York, NY, USA; 2 Department of Neurology, Saint Louis University School of Medicine, St. Louis, MO, USA; 3 Taub Institute for Research on Alzheimer’s Disease and the Aging Brain, Vagelos College of Physicians and Surgeons, Columbia University, New York, NY, USA; 4 The Gertrude H. Sergievsky Center, Vagelos College of Physicians and Surgeons, Columbia University, New York, NY, USA; 5 Biostatistics Department, Mailman School of Public Health, Columbia University, New York, NY, USA; 6 Department of Biostatistics, School of Public Health, Boston University, Boston, MA, USA; 7 Johns Hopkins University School of Medicine, Baltimore, MD, USA; 8 Brown Foundation Institute of Molecular Medicine, Mc Govern Medical School, The University of Texas Health Science Center at Houston, Houston, TX, USA; 9 Department of Neurology, Boston University School of Medicine, Boston, MA, USA; 10 Department of Neurology, University of Miami Miller School of Medicine, Miami, FL, USA; 11 Department of Public Health Sciences, University of Miami Miller School of Medicine, Miami, FL, USA; 12 Evelyn F. McKnight Brain Institute, University of Miami Miller School of Medicine, Miami, FL, USA; 13 Department of Epidemiology, Mailman School of Public Health, Columbia University, New York, NY, USA; 14 The Glenn Biggs Institute for Alzheimer’s and Neurodegenerative Diseases, University of Texas Health Sciences Center, San Antonio, TX, USA; 15 School of Medicine and Research Center, Universidad Espíritu Santo – Ecuador, Samborondón, Ecuador; 16 Memory Aging and Cognition Center, Department of Pharmacology, Yong Loo Lin School of Medicine, National University of Singapore, Singapore; 17 University of Maryland School of Medicine, Baltimore, MD, USA; 18 Human Genetics Center, School of Public Health, The University of Texas Health Science Center at Houston, Houston, TX, USA

**Keywords:** Asymptomatic Intracranial Large Artery Stenosis, Atherosclerosis, Epidemiology, GWAS, Lysophosphatidylglycerol Acyltransferase 1, Never In Mitosis Related Kinase 2

## Abstract

**Background:**

Intracranial large artery stenosis (ILAS) is one of the most common causes of stroke worldwide and is associated with the risk for future vascular events. Asymptomatic ILAS is a frequent finding on neuroimaging and shares many risk factors with atherosclerotic vascular disease. Whether asymptomatic ILAS is driven by genetic variants is not well-understood.

**Methods and Results:**

This study included 4960 participants from seven geographically diverse population-based cohorts (34% Whites, 16% African Americans, 22% Hispanics, 24% Asians, 5% native Ecuadorians). We defined asymptomatic ILAS as luminal stenosis > 50% in any large brain artery using time-of-flight magnetic resonance angiography (MRA). A genome-wide association study revealed one variant in *RP11–552D8.1* (rs75615271; OR, 1.22 [1.11–1.33]; *P*=4.85×10^−8^) associated with global ILAS at genome-wide significance (*P*<5×10^−8^). Gene-based association analysis identified a gene-set enriched in chr1q32 region, including *NEK2*, *LPGAT1*, *INTS7*, *DTL*, and *TMEM206*, in global ILAS (*P*=1.34 ×10^−7^) and anterior ILAS (*P*=1.77 ×10^−8^).

**Conclusion:**

This study reveals one variant rs75615271 associated with asymptomatic ILAS in a multi-population. Further functional studies may help elucidate the role that this variant plays in the pathophysiology of asymptomatic ILAS.

## Background

Intracranial large artery stenosis (ILAS), most often caused by intracranial atherosclerotic disease, is one of the common causes of stroke and is associated with the risk for future vascular events ^1–3^. ILAS is considered to be a major cause of stroke, accounting for 30–50% of cases of ischemic stroke in the Asian, non-Hispanic Black, and Hispanic populations, but only about 10% in non-Hispanic whites of European descent^4^. The prevalence of ILAS varies greatly according to racial/ethnic origin, e.g., ILAS is much less frequent in Western countries than in Asia^5^. Various explanations for racial/ethnic differences in the prevalence of ILAS have been advanced, including genetic susceptibility of intracranial vessels to atherosclerosis, as well as differences in lifestyle and risk factors, such as hypertension, diabetes, dyslipidemia, and smoking^6^. Previously, a case-control study revealed that variant *RNF213* c.14429G > A (p.Arg4810Lys, rs112735431) has a strong association with ILAS^7^. A recent study revealed that *RNF213* p.Arg4810Lys also increases the risk of ischemic stroke due to intracranial artery atherosclerosis^8^. Moreover, *RNF213* p.Arg4810Lys was associated with coronary artery disease and pulmonary hypertension^9–11^. The association between *RNF213* and stenosis of the intracranial, coronary, and other systemic arteries indicates that genetic traits may partially account for intracranial stenosis, either by predisposing vascular risk factors or by a direct contribution to an established atherosclerotic mechanism. Other candidate genes implicated in previous genetics studies with ILAS include *ADIPOQ*, *PDE4D*, *LPL*, and *CYP11B2*^12–15^. Asymptomatic ILAS is a frequent finding on neuroimaging. The prevalence of asymptomatic ILAS increases with age in patients with transient ischemic attack and minor stroke^16^. A study in a stroke-free population indicated that asymptomatic ILAS is a risk factor for cerebral and systemic vascular events with risk increasing as stenosis severity worsens ^17^. Asymptomatic ILAS shares many risk factors with atherosclerotic vascular disease. However, whether asymptomatic ILAS is driven by common and rare genetics is not well-understood. In this study, we performed a multi-population discovery genome-wide association study (GWAS) analysis in diverse population cohorts within and outside the United States. We detailed the variants, genes, and biologic pathways relevant to the genetic architecture of asymptomatic ILAS and described the similarities and differences between asymptomatic ILAS and other clinical cardiovascular diseases (stroke, coronary artery disease, atrial fibrillation, etc.) using Mendelian randomization, gene association analysis, and expression quantitative trait loci colocalization, and by exploring shared overlap with other large population intracranial stenosis GWAS research.

## Method

### Sampled populations

#### Atherosclerosis Risk in Communities (ARIC) study

The ARIC study is a population-based prospective cohort investigating vascular risks. It includes 15,792 persons aged 45–64 years at baseline (1987–1989), randomly selected from four US communities^18^. Participants completed seven clinic examinations conducted from 1987 to 2019. The institutional review boards at the collaborating medical institutions (The Johns Hopkins University, University of North Carolina at Chapel Hill, Wake Forest University, University of Mississippi Medical Center, and University of Minnesota) approved the study. All participants provided written informed consent.

#### The Northern Manhattan Study (NOMAS)

NOMAS is a prospective cohort initially focused on determining the incidence of stroke and vascular events in a diverse urban population^19^. Participants were recruited through random digit dialing from 1993 to 2001. Written informed consent was provided by all study participants. The study was approved by institutional review boards at Columbia University Medical Center and the University of Miami.

#### Washington Heights–Inwood Columbia Aging Project (WHICAP study)

WHICAP is a prospective, population-based study focused on aging and dementia. Established through multiple recruitment waves, participants were first recruited in 1992 from a random sample of Medicare-eligible adults residing in the neighborhoods of Washington Heights and Inwood in northern Manhattan. Participants undergo evaluations every 18–24 months, which include a comprehensive neuropsychological battery, medical and neurologic examination, and health-related survey^20^. Dementia and its subtypes are determined in a consensus conference involving neurologists and neuropsychologists. The study was approved by the institutional review board at Columbia University Medical Center. All participants provided written informed consent.

#### Epidemiology of Dementia In Singapore (EDIS study)

The EDIS study is a cross-sectional design drawing participants who were long-term local citizens from the Singapore Epidemiology of Eye Disease (SEED) study, consisting of the Singapore Chinese Eye Study, Singapore Malay Eye Study-2, and the Singapore Indian Eye Study-2, aged 60 years and above ^21–23^. Specific enrolment targets were set for the Chinese, Malay, and Indian communities to ensure that the final cohort was proportionate to Singapore’s ethnic composition. The Singapore Eye Research Institute Review Board approved the study. Bilingual coordinators obtained written informed consent from participants in their preferred language prior to enrollment.

#### Memory Clinic in Singapore (MCS study)

The MCS study involved patients attending the memory clinics at National University Hospital and St. Luke’s Hospital from 2009 to 2015. Patients were referred by primary, secondary and tertiary care facilities due to consistent memory complaints and were assessed by a team of clinicians, psychologists, and nurses in the Memory Aging and Cognition Center, National University of Singapore. The institutional review board at National University Hospital approved the study. All participants provided written informed consent.

#### Framingham Heart Study (FHS)

FHS is a prospective, community-based cohort. Participants who survived to the 7th examination were invited to undergo a brain MRI between 1999 to 2005, with a final sample of 2144 stroke-free individuals. For these analyses, we used an FHS subsample with available MRA as part of the stroke case study. The study was approved by the institutional review board at Boston Medical Center. All participants provided written informed consent.

#### The Atahualpa Project (TAP)

TAP is a population-based study designed to evaluate prevalence, incidence, and correlates of major neurological and cardiovascular disorders in community residents aged ≥ 40 years in rural Ecuador. The neuroimaging sub-study enrolled all Atahualpa residents aged ≥ 60 years who had no contraindications for magnetic resonance imaging and provided the informed consent ^24^. The study was approved by the Institutional Review Board of Hospital-Clínica Kennedy, Guayaquil.

We followed the STrengthening the Reporting of OBservational studies in Epidemiology (STROBE) reporting guidelines for cohort studies ^25^.

### Measurement of Asymptomatic ILAS

The software LKEB Automated Vessel Analysis (LAVA) (Leiden University Medical Center, The Netherlands, build date October 19th, 2018) was utilized to view all MRA images that collected from each site; then analyze the images centrally and determine the presence and severity of intracranial arterial stenosis. Briefly, this software uses a flexible 3D tubular Non-Uniform Rational B-Splines model to automatically identify the margins of the arterial lumen based on voxel intensity with excellent reliability ^12^. A prespecified and harmonized imaging analysis protocol ^26^ was employed to measure cross-sectional arterial diameters for up to 13 intracranial arteries per participant, including left and right anterior cerebral arteries (ACAs), middle cerebral arteries (MCAs), internal carotid arteries (ICAs), posterior cerebral arteries (PCAs), posterior communicating arteries (PCOMs) and intracranial portion of vertebral arteries (VAs); and the basilar artery (BA). The severity of intracranial stenosis was evaluated and quantified by two trained neurologists independently according to the narrowest lumen area compared with the immediately preceding normal segment, or the next normal appearing lumen if the stenosis was at the arterial origin. Stenosis was classified as clinically relevant when it was estimated to be equal to or larger than 50% of the normal lumen ^27^. Asymptomatic ILAS was defined as presence of one or more stenotic vessels on MRA, where the stenosis is equal to or larger than 50% of the normal lumen. Anterior ILAS referred to asymptomatic ILAS in ICAs, MCAs, ACAs, and PCOMs; posterior ILAS referred to asymptomatic ILAS in PCAs, VAs and BA; global ILAS referred to asymptomatic ILAS detected in any of the intracranial arteries.

### Genome-Wide Association Study

Description of genotyping, quality control and imputation in each study is provided in supplemental Table 1. In brief, sample and variant quality control criteria included the following: 1) <10% missingness of genotype calls, 2) Hardy-Weinberg Equilibrium p-value <1×10^−6^, 3) imputation quality < 0.3, 4) minor allele frequency (MAF) <0.0001, 5) call rate < 97.5% for MAF > 1% and call rate < 99% for MAF < 1%.

GWAS was performed separately for each race/ethnicity ^28^ group in each cohort. We used multiple logistic regression in PLINK with adjustment for age, sex, and three principal components, and filtered for variants with minor allele frequency >1%. EasyQC was employed to conduct quality control for GWAS results. Multi-population results were determined using a fixed-effect inverse-variance-based method implemented in METAL ^29^. Variants with minor allele frequencies (MAF) < 1% and those not present in at least two studies were excluded after the meta-analyses. Cross-study heterogeneity was assessed using Cochran’s Q-test, and variants with heterogeneity p-value <0.05 were excluded. Population-specific meta-analyses were performed to identify population-specific variants. An association with a *P* < 5×10^−8^ was considered genome-wide significant.

GWAS lead SNPs (*P* < 1×10^−5^) and genomic regions were annotated on the web-based platform FUMA (Functional Mapping and Annotation of Genome-Wide Association Studies)^30^. Briefly, pairwise linkage disequilibrium statistics were calculated from a 1000 Genomes Project Phase 3 reference panel (mixed for multi-population meta-analysis, White, African American, American admixed for Hispanic individual, Asian, and Amerindian). Independent GWAS loci were identified using R^2^ < 0.6 (the default parameter in FUMA) and independent lead single nucleotide polymorphisms (SNPs) were further selected from the set of independent GWAS variants using R^2^ < 0.1. Candidate SNPs are in LD with any of the independent significant SNPs at R^2^ > 0.6. A genomic region was then determined using the calculated linkage-disequilibrium structure (multiple independent SNPs were merged if < 250 kb from each linkage-disequilibrium block).

### Gene-based association and gene enrichment analysis

All GWAS variants were annotated by location (intergenic, intron, exon) and nearest gene using the single nucleotide polymorphism database (dbSNP) of nucleotide sequence information. Gene-set analysis was performed for multi-population results using Multi-Marker Analysis of GenoMic Annotation (MAGMA)^31^ implemented in the FUMA^30^. Gene sets were obtained from Msigdb v7.0^32^. Gene associations were considered significant if they met a p-value < 2.6×10^−6^ (0.05/19,021 protein coding genes). Candidate SNPs were mapped to the nearest gene within 50kb or an expression quantitative trait locus (eQTL) genes in Genotype-Tissue Expression (GTEx) project data version 8 (v8)^33^.

Mutation intolerance was calculated by probability of being loss-of-function intolerant (pLI) score from ExAC database^34^ and non-coding residual variation intolerance score (ncRVIS)^35^. The higher the pLI score is, the more intolerant to loss-of-function mutations the gene is. The higher the ncRVIS is, the more intolerant to noncoding variants the gene is. To explore the interaction between the target region and multiple genes, chromatin interaction mapping was performed. The Hi-C data was used to identify with significant chromatin interactions at FDR= 1×10^−6 36^. To test the relationship between highly expressed genes in a specific tissue and genetic association, gene-property analysis is performed using average expression of genes per tissue type as a gene covariate. Gene expression values are log2 transformed average Reads Per Kilobase of transcript per Million mapped reads (RPKM) per tissue type based on GTEx RNA-seq data. Tissue expression analysis is performed for 30 general tissue types and 53 specific tissue types in GTEx v8 database, separately. MAGMA was performed using the result of gene analysis (gene-based P-value) and tested for one side (greater) with conditioning on average expression across all tissue types.

### Mendelian Randomization Analysis

Mendelian randomization was performed to select biomarkers previously identified as risk factors or relevant to pathobiology for asymptomatic ILAS with ischemic stroke^37^, small vessel stroke^37^, atrial fibrillation (AF)^38^, and coronary artery disease^39^. To minimize bias from correlated instruments, variants with asymptomatic ILAS association p-value <1.0×10^−5^ were LD-clumped at r^2^ < 0.01 ^40^ against the 1000 Genome LD reference calculated for White, African American, Asian and Hispanic, and Ecuadorian populations. Variants with MAFs < 0.01 in the reference population were excluded from MR analysis. Causal association was primarily evaluated using the inverse-variance weighted (IVW) method, additionally performed sensitivity analysis using simple median-based method, weighted median-based method, and MR-Egger method. All MR analyses were performed using the “TwoSampleMR” R package ^40^.

## Results

### Multi-population GWAS Identifies a Novel Locus Associated with Asymptomatic ILAS

We conducted a multi-population GWAS for asymptomatic ILAS levels in 4960 participants, including 1677 Whites, 799 African Americans, 1064 Hispanics, 1191 Asians, and 229 native Ecuadorian. Mean age of the participants across studies ranged from 67 to 76 years, with proportions of women ranging from 40% to 64%. Detailed demographic information is presented in Table 1.

**Figure 1:**
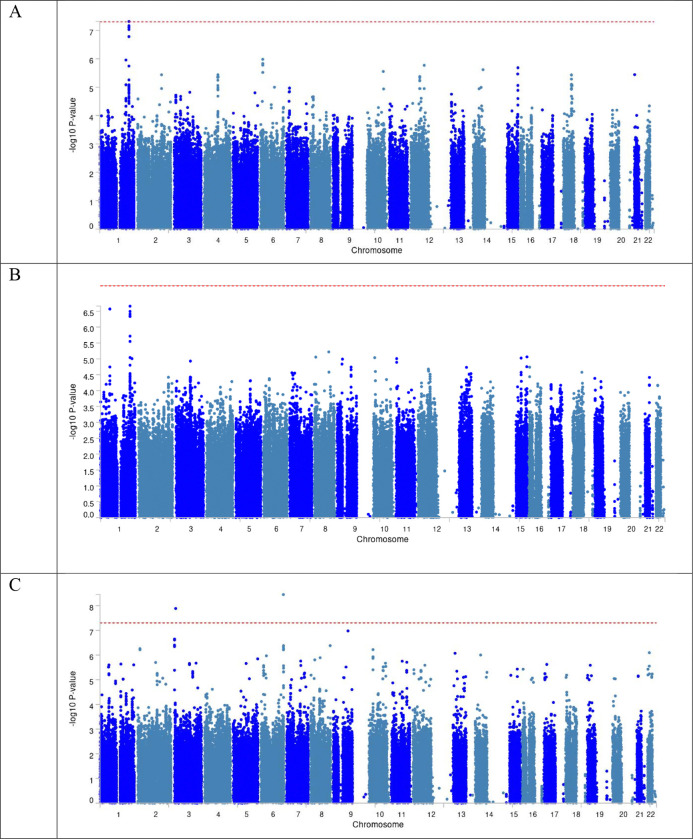
Manhattan Plot of GWAS summary statistics. (A) Global ILAS. (B) Anterior ILAS. (C) Posterior ILAS.

**Figure 2:**
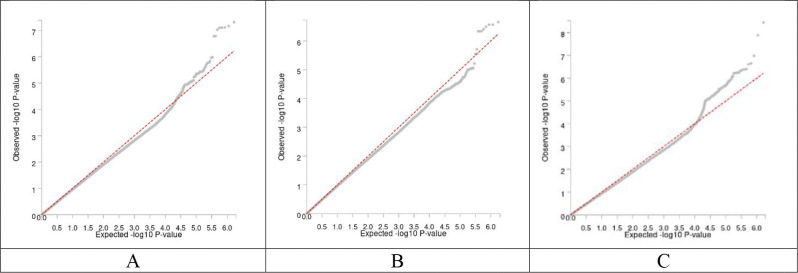
QQ plot. (A) Global ILAS. (B) Anterior ILAS. (C) Posterior ILAS.

We identified one variant rs75615271 (*RP11–552D8.1*) associated with global ILAS at genome-wide significance (*P*<5×10^−8^; Figure 1A). One copy of the A allele (AF=0.11) for rs75615271 (OR, 1.22 [1.11–1.33]; *P*=4.85×10^−8^) was associated with increased risk of global ILAS (Table 2). We did not find the variants associated with anterior or posterior ILAS at genome-wide significance. However, we identified rs75615271 as the lead variant that was associated with anterior ILAS (OR, 1.16 [1.08–1.25]; *P*=2.19×10^−7^). The genomic control lambda value for was 1.03 for global ILAS GWAS, 1.02 for anterior ILAS GWAS and 1.08 for posterior ILAS GWAS (Figure 2). We did not observe population-specific variants related to asymptomatic ILAS based on the population-specific meta-analyses at genome-wide significance (Supplemental Figure 1–6, Supplemental Table 2).

### Gene-based Association Analysis and Gene-set Enrichment

**Figure 3:**
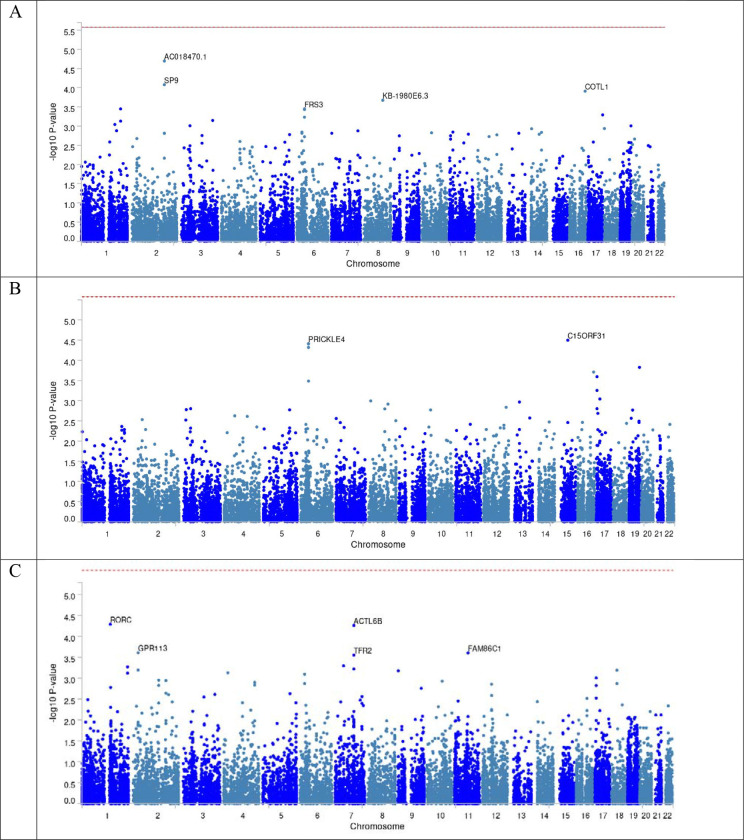
Mahattan Plot of gene-based test. (A) Global ILAS. (B) Anterior ILAS. (C) Posterior ILAS.

**Figure 4:**
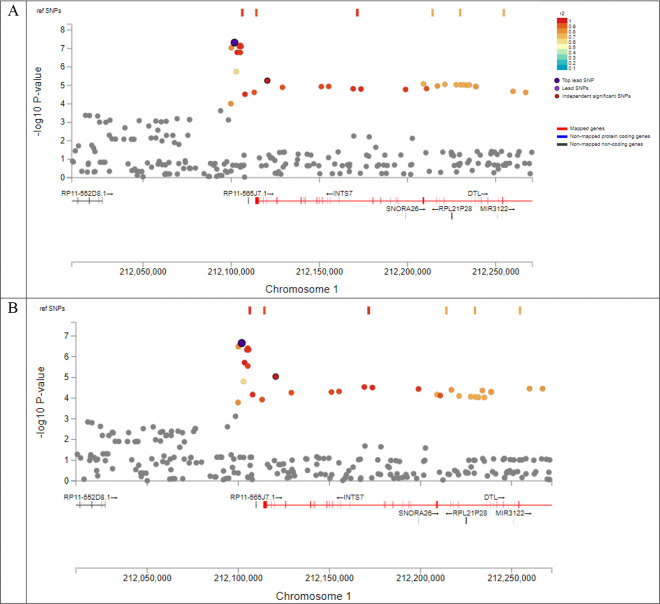

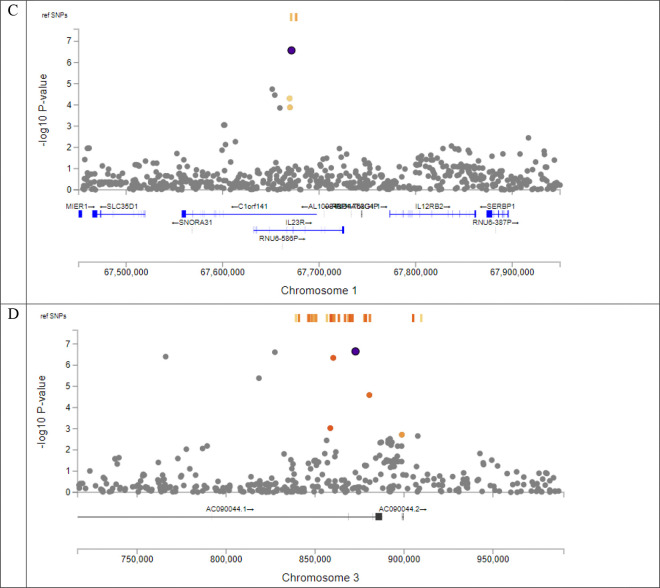

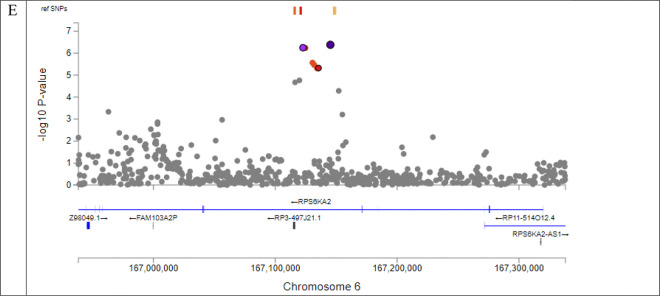
Regional plot. (A) rs75615271 (1:212101892:A:G) for global ILAS. (B) rs75615271 (1:212101892) and (C) rs79148417 (1:67671368) for anterior ILAS. (D) rs62238282 (3:872733) and (E) rs78189747 (6:167145219) for posterior ILAS.

We performed Gene-based association test and did not observe gene-based genome-wide significant associations with asymptomatic ILAS (Figure 3). The top gene was *AC018470.1* (*P*=2.02×10^−5^) for global ILAS, *C15ORF31* (*P*=3.19×10^−5^) for anterior ILAS, and *RORC* (*P*=5.20×10^−5^) for posterior ILAS, respectively. All candidate SNPs, which are in LD of any independent lead SNPs, were mapped to genes. Figure 4 shows the regional plot of top SNPs in global, anterior and posterior ILAS. Based on pLI score and the non-coding residual variation intolerance score, the most intolerant genes were *PIGN* for global ILAS, *SDE2* for anterior ILAS and *BCO2* for posterior ILAS (Table 3). The MAGMA gene-based association analysis identified one gene-set associated with anterior ILAS (Bonferroni adjusted *P*=0.035) and posterior ILAS (Bonferroni adjusted *P*=0.033), respectively (Table 4). These gene-sets were KEGG non-homologous end-joining pathway (ko03450) associated with anterior ILAS and microtubule bundle formation (GO:0005879) associated with posterior ILAS (Table 5). Genes mapped to candidate SNPs were further investigated for gene-set enrichment and functional consequences against reference panels (Table 6). We found a gene set of global ILAS and anterior ILAS that was enriched in chr1q32 region, including *NEK2*, *LPGAT1*, *INTS7*, *DTL*, and *TMEM206*. Additionally, a gene set was enriched in chr10q24 in posterior ILAS (*P*=2.48×10^−20^). Furthermore, we identified one Hispanic-specific gene(*P2RX5*) related to anterior ILAS, and one Asian specific gene (*TMPRSS7*) related to posterior ILAS (Supplemental Figure 7–9). The MAGMA gene-based association analysis identified two gene-sets associated with Hispanic specific anterior ILAS (positive regulation of immature T cell proliferation, adjusted *P*=0.002; T cell activation, adjusted *P*=0.032); and one gene-set (Reactome SARS-CoV-1 infection pathway, adjusted *P*=0.008) associated with White-specific posterior ILAS (Supplemental Table 3 and 4).

### Tissue-Specific Colocalization Analyses

**Figure 5.**
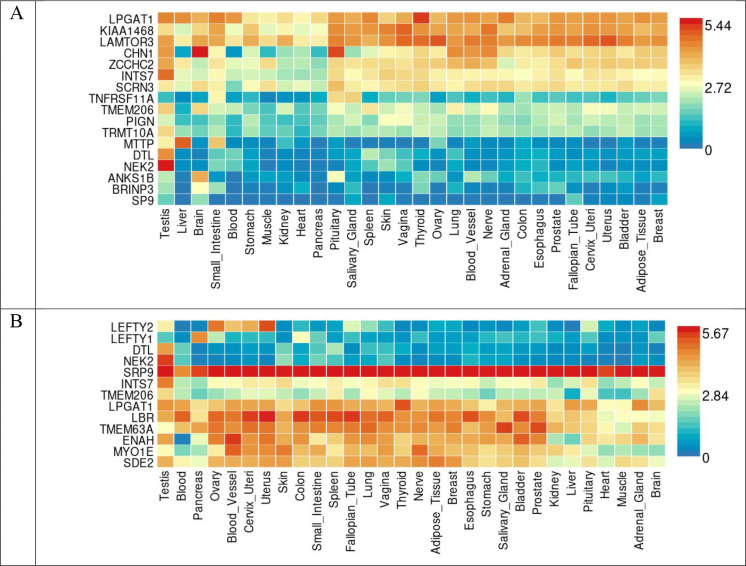

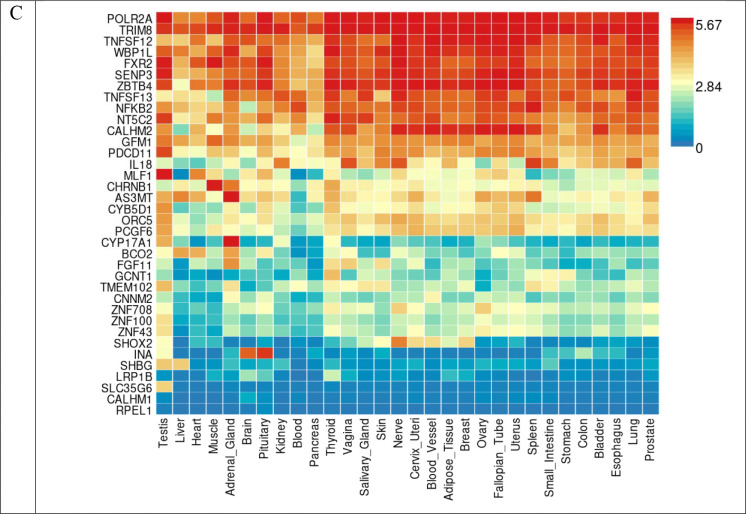
Gene expression heatmap in GTEx v8 dataset 30 general tissue types. (A) Global ILAS. (B) Anterior ILAS. (C) Posterior ILAS.

**Figure 6.**
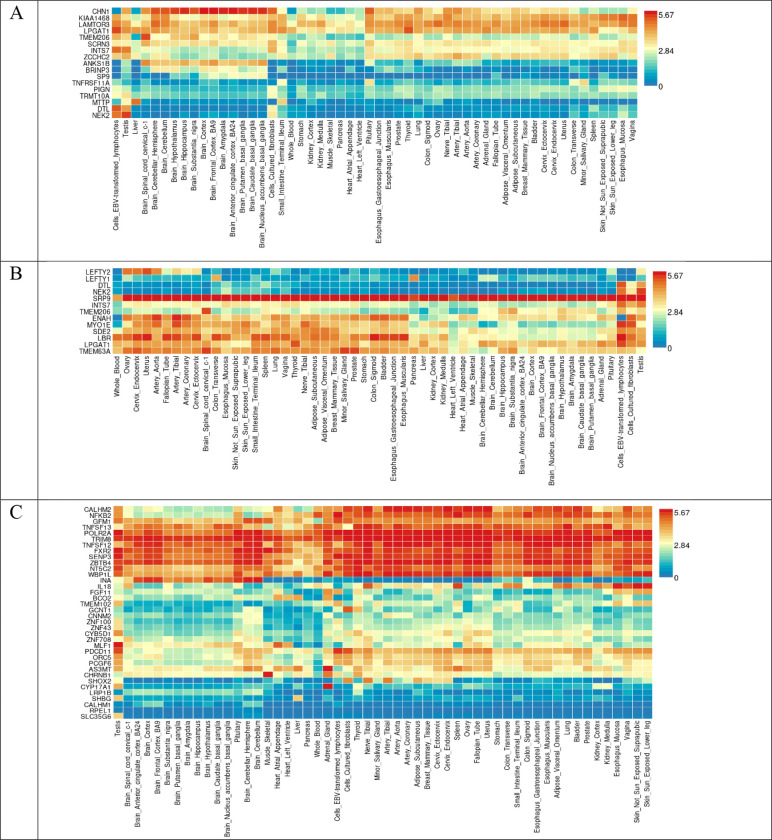
Gene expression heatmap in the GTEx v8 dataset 54 tissue types. (A) Global ILAS. (B) Anterior ILAS. (C) Posterior ILAS.

**Figure 7.**
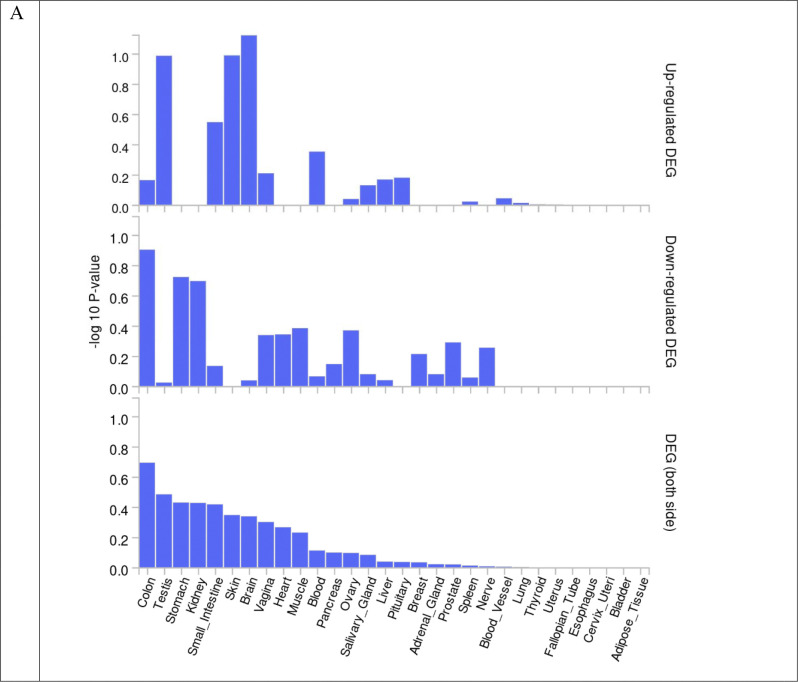

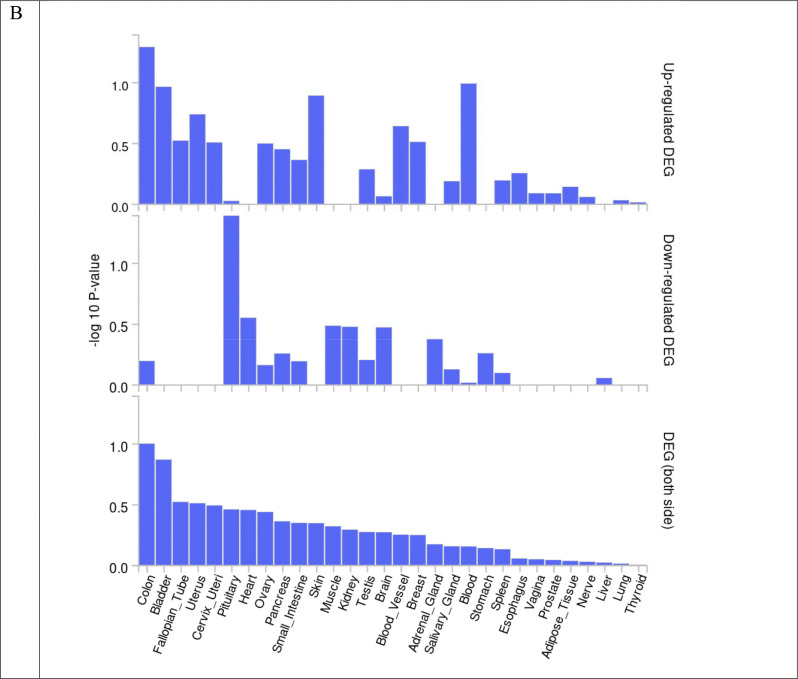

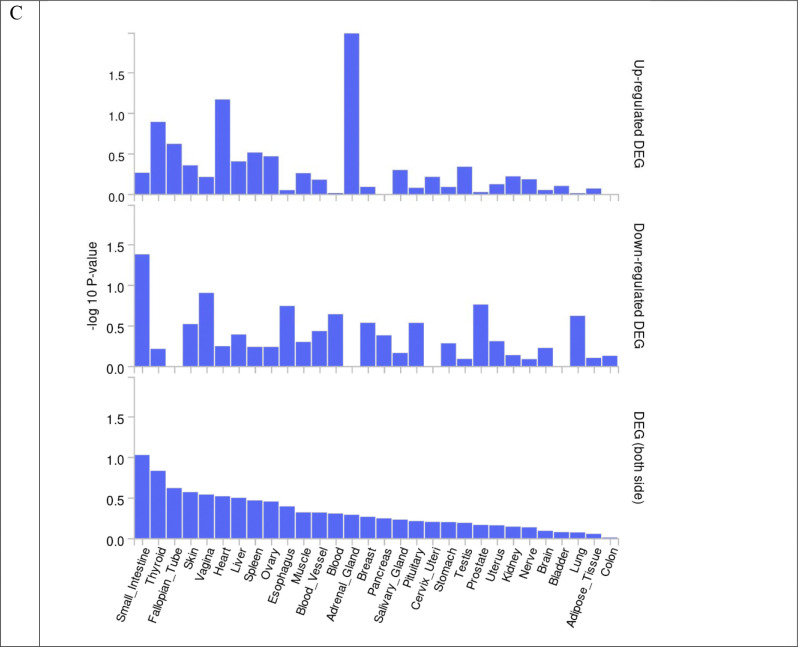
Enrichment test of differentially expressed genes in GTEx v8 dataset 30 general tissue types. (A) Global ILAS. (B) Anterior ILAS. (C) Posterior ILAS.

**Figure 8.**
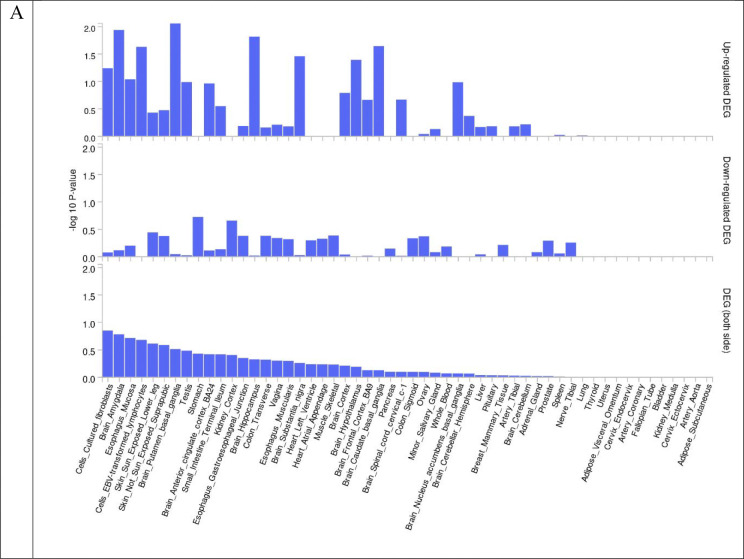

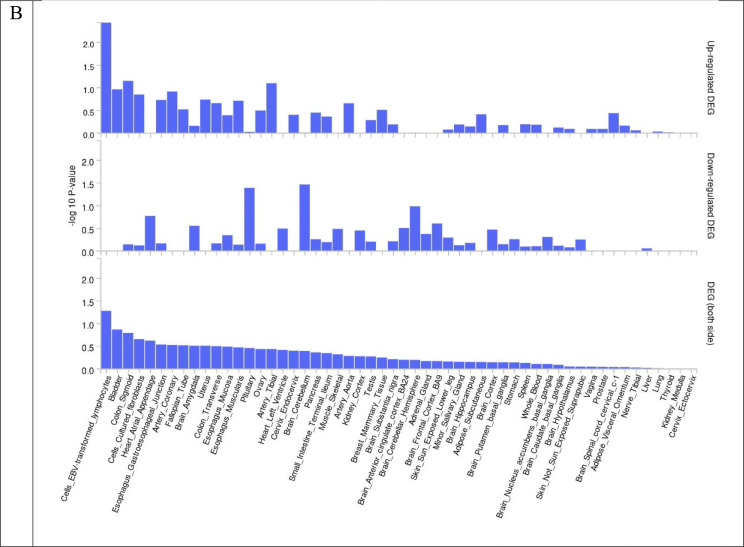

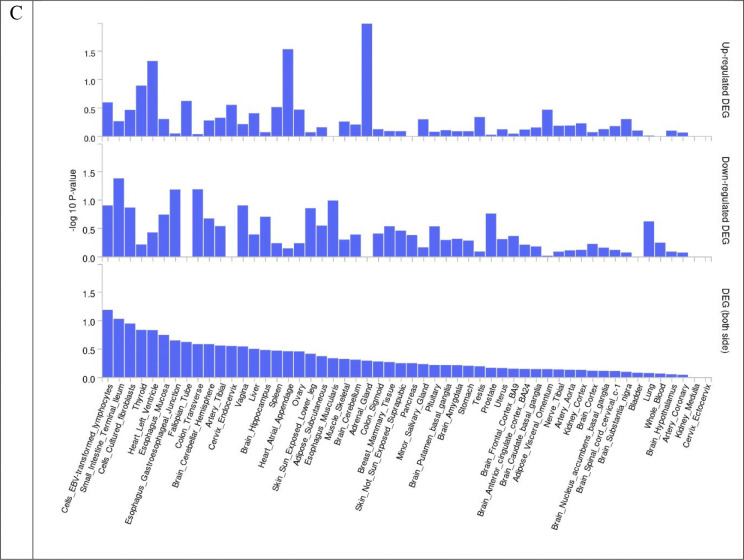
Enrichment test of differentially expressed genes in GTEx v8 dataset 54 tissue types. (A) Global ILAS. (B) Anterior ILAS. (C) Posterior ILAS.

We performed colocalization analysis for the locus identified in the GWAS analysis and gene-based analysis with gene expression using Genotype-Tissue Expression v8 eQTL data (Table 7). Tissue specific gene expression of 30 general tissue types and 53 specific tissue types of Genotype-Tissue Expression eQTL v8 were presented by heatmap (Figure 5 and 6). In the analysis of 30 general tissues, we identified that *CHN1* associated with global ILAS had higher mRNA expression in the brain tissue (Figure 5). We did not observe the differentially expressed genes based on the enrichment test (Figure 7 and 8).

**Figure 9:**
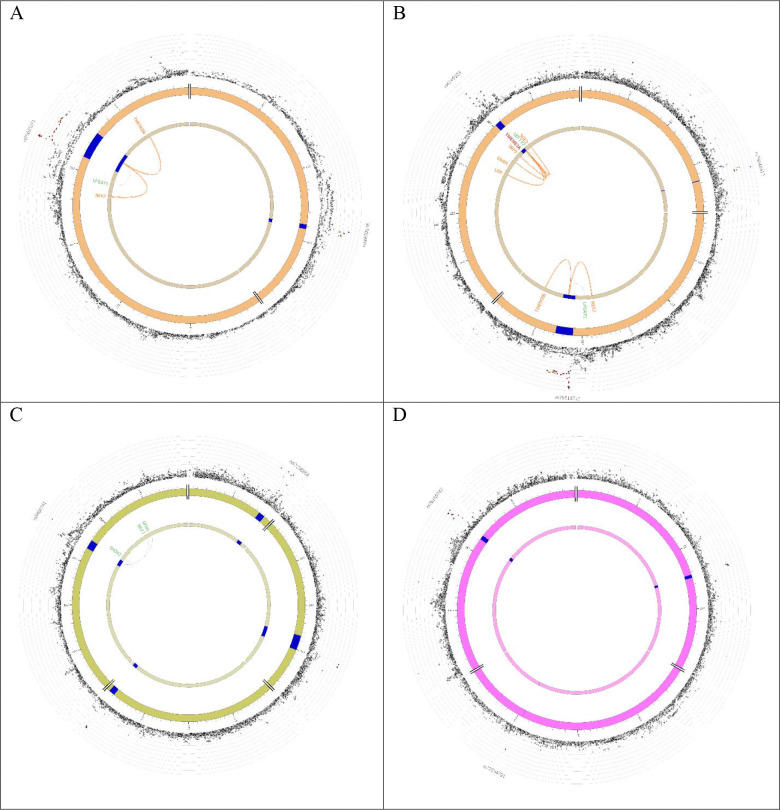
Circos plot for the genomewide level locus. Chromatin interaction between genomic risk locus (blue arc) and genes were showed by orange line. The green color line linked the top SNP and the eQTL genes. (A) Chromosome 1 top SNPs for Global ILAS. (B) Chromosome 1 rs75615271 top SNP for Anterior ILAS. (C) Chromosome 3 top SNP for Posterior ILAS. (D) Chromosome 6 top SNP for Posterior ILAS.

The circos plot showed multiple chromatin interactions between the genomic risk locus on chromosome 1 and genes *TMEM206*, *NEK2*, and *LPGAT1* for global ILAS (Figure 9A); chromosome 1 and genes *SDE2*, *LEGTY2*, *SRP9*, *ENAH*, *LBR*, *TMEM63A*, *TMEM206*, *NEK2*, and *LPGAT1* for anterior ILAS (Figure 9B). For the posterior ILAS, the loci associated with lead SNPs had chromatin interaction with multiple genes (Figure 9C).

### Mendelian Randomization

**Figure 10:**
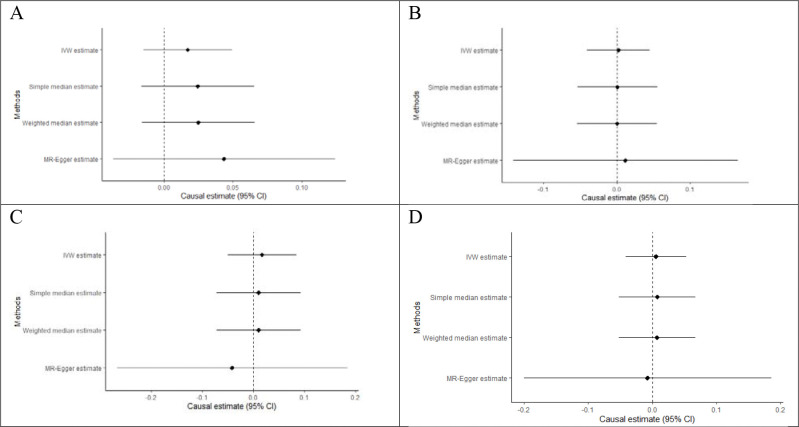
Forest plots of Two-sample MR analysis. (A) Ischemic stroke. (B) Small vessel stroke. (C) Coronary Artery Disease. (D) Atrial Fibrillation.

To establish a causal pathway from ILAS to stroke, coronary artery disease, and atrial fibrillation, we performed a Mendelian Randomization (MR) analysis. We did not observe any association of ILAS with stroke, cardiovascular disease, or atrial fibrillation (Table 8, Figure 10).

## Discussion

This is the first study to investigate the genetic determinants of asymptomatic ILAS in multi-populations. We identified associations of novel genetic loci with asymptomatic ILAS genetic architecture. Beyond mapping to the nearest genes, we also showed the biological impact of our findings using in silico functional analyses. Our results demonstrated that genetic loci are coupled with gene expression information, which imply biologically relevant pathways.

In this multi-population analysis, we observed a novel variant rs75615271 mapped to *RP11–552D8.1* that has a significant association with global ILAS. *RP11–552D8.1* is PGAT1 antisense RNA 1 (LPGAT1-AS1), located in the Chr1p31, which plays a crucial role in regulating gene expression at multiple levels. Lysophosphatidylglycerol acyltransferase 1 (*LPGAT1*) encodes protein that catalyzes the reacylation of lysophosphatidylglycerol into phosphatidylglycerol. It is a key precursor for the cardiolipin synthesis, which is involved in lipid biosynthesis ^41^. *LPGAT1* has been reported to regulate the biosynthesis of triacylglycerol, which is important for maintaining phospholipid homeostasis and modulating the structural integrity of mitochondrial membranes^42^. Additionally, *LPGAT1* plays a role in lipid metabolism, and its impact on body mass index and body fat have been confirmed ^43^. These effects of *LPGAT1* on the organism occur when it is in its regular expression profile, but elevated expression levels may contribute to the development of certain diseases. Previous studies showed that *LPGAT1* gene expression is upregulated in tumor tissue compared to normal tissue ^44–46^. In our study, the variant *RP11–552D8.1* rs75615271 was associated with presence of asymptomatic ILAS.

A gene set associated with asymptomatic ILAS was identified, including *LPGAT1*, never in mitosis related kinase 2 (*NEK2*), integrator complex subunit 7 (*INTS7*), denticleless E3 ubiquitin protein ligase homolog (*DTL*), proton activated chloride channel 1 (*PACC1*, also known as *TMEM206*).

*NEK2* encodes a serine/threonine-protein kinase that is essential for mitotic regulation. This protein localizes to the centrosome, and undetectable during G1 phase, but accumulates progressively throughout the S phase, reaching peak levels in late G2 phase^47^. *NEK2* is associated with poor prognosis of clear cell renal cell carcinoma and promotes tumor cell growth and metastasis ^48^. High expression of *NEK2* was associated with vascular invasion and tumor grade in multiple patient cohorts of pancreatic cancer^49^. *NEK2* is abnormally overexpressed in a wide range of human cancers and is implicated in various aspects of malignant transformation, including tumorigenesis, drug resistance and tumor progression^50^.

*INTS7* encodes a subunit of the integrator complex that is associated with the C-terminal domain of RNA polymerase II and mediates 3’-end processing of the small nuclear RNAs U1 and U2. The expression level of *INTS7* may correlate with tumor microenvireoment, immunotherapy responsiveness^51^. Several studies showed that *INTS7* is upregulated in several solid tumors, such as cholangiocarcinoma, hepatocarcinoma, cervical squamous cell carcinoma, endocervical adenocarcinoma, and breast cancer ^52^. In addition, *INTS7* has been linked to bipolar disorder ^53^. *DTL*, also known as *CDT2* gene, contributes to ubiquitin-protein transferase activity that is involved in several key processes, including protein ubiquitination, regulation of G2/M transition of mitotic cell cycle, and translesion synthesis^54^. *CDT2* contains multiple WD40-repeat domains that play an essential role in regulating the *CDT1* degradation after DNA damage ^55^. Previous studies have shown that the CRL4-CDT2 complex, together with Rad6/18, monoubiquitinated PCNA promote the translation DNA synthesis in undamaged cells ^56^. In addition, the CRL4-CDT2 complex can degrade DNA replication-related proteins in a proteasome-dependent manner during DNA replication, implying a crucial role of *CDT2* in the regulation of DNA replication ^57^. Further research revealed that *CDT2* is augmented in head and neck squamous cell carcinoma (HNSCC) and is necessary for those tumor cells to proliferate. Its main role is to inhibit abnormal DNA replication. Inactivation of CRL4-CDT2 increases the radiosensitivity of HNSCC cells^58^. Moreover, USP46 protein could mediate the stability of *CDT2* and promote the growth of HPV-positive tumors, suggesting the potential role of *DTL* in tumor progression ^59^. *PACC1*, also known as *TMEM206*, is an integral component of plasma membrane, which is involved in pH-gated chloride channel activity and chloride transport^60^. Previous studies revealed that *TMEM206* is linked to cell volume changes under acidic pH and had the functions of the proton-activated Cl^−^ channel^61–64^. A recent study indicated the key role of *TMEM206* in macropinosome resolution ^65^. Macropinocytosis is of central importance for cancer cells, as they employ it to take up nutrients and proliferate in hypoxic, acidic and nutrient poor environments ^66^.

Purinergic receptor P2X 5 (*P2RX5*) was identified as a Hispanic-specific gene related to anterior ILAS. P2X5 is a member of the P2X family of ATP-gated nonselective cation channels, which exist as trimeric assemblies^67^. *P2RX5* encodes the P2X5 purinergic receptor, a ligand-gated ion channel activated by ATP, and plays a role in endothelial cell differentiation and autocrine regulation ^68, 69^, as well as having functional roles in adult mouse astrocytes ^70^. A study showed surface and intracellular *P2RX5* expression was upregulated in activated antigen-specific CD4+ T cell clones, which indicated a functional role of the human *P2RX5* splice variant in T cell activation and immunoregulation ^71^. In humans, *P2RX5* exists as a natural deletion mutant lacking amino acids 328–349 of exon 10, meaning that only a proportion of the human population express fully functional P2X5 receptors, and amino acid substitutions within this gene can markedly impact the receptor’s responsiveness to its ATP ligand ^72^. A study using DNA methylation and genetics indicated *P2RX5* have related roles in the cerebrovascular system ^73^ .

Transmembrane Serine Protease 7 (*TMPRSS7*) was identified in an Asian-specific gene related to posterior ILAS. *TMPRSS7* encodes the protein that belongs to the type II transmembrane serine protease family, the 17 human members. They play physiological and pathological roles in digestion, cardiac function, and blood pressure regulation ^74–76^. They have also been implicated in tumor growth, invasion and metastasis, and the genetic variant rs1844925 of *TMPRSS7* has been associated with the risk for and prognosis of breast cancer ^77^. Another study reported that rs147783135 of *TMPRSS7* was related to ischemic stroke, with the minor T allele being protective against this condition ^78^. Given the potential roles of *TMPRSS7* in tumor growth and blood pressure regulation, the association of this gene with ischemic stroke may reflect an effect on atherosclerosis or blood pressure^74–76^.

Our study has some limitations that need to be recognized. First, due to the relatively modest sample sizes of each population, the statistical power to detect population-specific associations or functional associations was limited. Consequently, imbalance of cases and controls across different datasets may limit the applicability of the study’s finding to population-specific groups. Lastly, different datasets were genotyped using different GWAS platforms. It is not clear how this might have affected the imputation quality.

## Conclusions

In summary, we identified one significant variant associated with asymptomatic ILAS in a multi-population. Our study provides insights into a potential biological mechanism for the association between these loci and asymptomatic ILAS. Identifying genes associated with these loci and understanding their function may help us to elucidate the mechanism through which asymptomatic ILAS may influence cerebrovascular health.

## Supplementary Material

Supplement 1

## Figures and Tables

**Figure 11:**
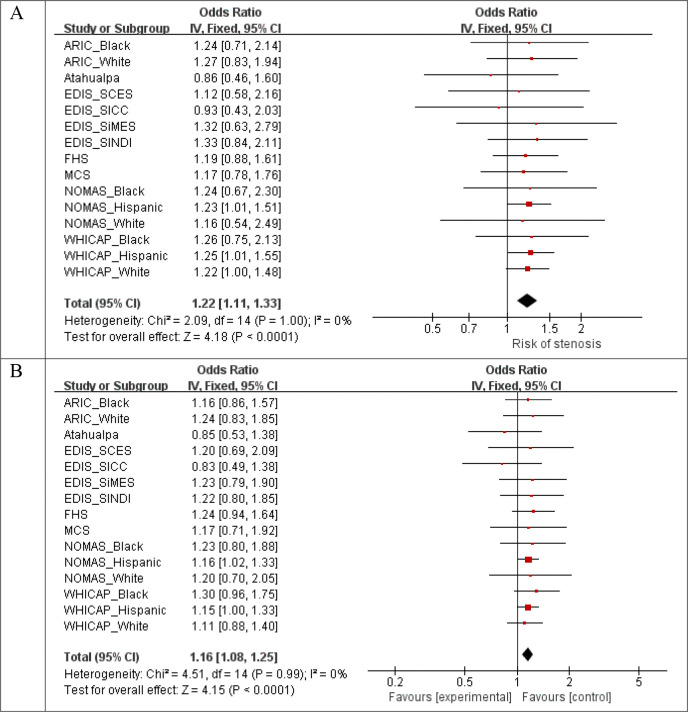

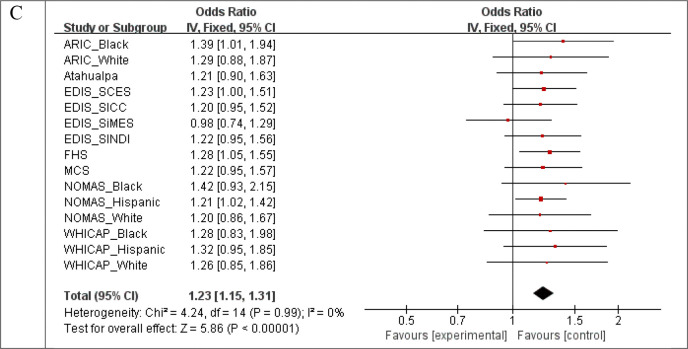
Forest plots for the meta-analysis of top variants. (A) rs75615271 in global ILAS. (B) rs75615271 in anterior ILAS. (C) rs62238282 in posterior ILAS.

**Table 1:** Demographic information of studies

Cohort	ARIC	NOMAS	EDIS	MCS	FHS	Atahualpa	WHICAP

N = 4960	1565	1109	841	350	206	229	660

Age, mean (sd)	75.8 (5.3)	70.1 (8.4)	70.2 (6.6)	71.0 (8.2)	72.7 (11.7)	66.7 (6.4)	76.4 (6.2)
Female (%)	58.8	60.5	51.7	55.7	54.3	40.1	64
Ancestry (%)							
European	74.3	13.8	-	-	100	-	23.4
African	25.7	16.6	-	-	-	-	32.2
Hispanic	-	69.6	-	-	-	-	44.2
Asian	-	-	100	100	-	-	
Native Ecuadorian	-	-	-	-	-	100	
Hypertension (%)	74.9	68.1	80.8	67.8	68.4	47.6	69
Diabetes (%)	33.9	19	37.3	31.8	23.9	32.3	30.1
Dyslipidemia (%)	56	45	76	71.4	51.6	17	51.5
Current Smoking (%)	6	52.7	27.9	7.1	9.3	1.7	5.3
Asymptomatic ILAS (%)	10.4	7.4	7.7	20.7	21	6.1	9.8
Anterior asymptomatic ILAS (%)	4.5	4.9	3.6	17.1	16.6	5.2	7.2
Posterior asymptomatic ILAS (%)	7.6	2.8	5.1	7.5	9	1.3	4

**Table 2:** Variants (P <5×10–8) associated with ILAS

Asymptomatic ILAS	uniqID	rsID	chr	pos	A1	A2	AF	OR	95% CI		P-value	Nearest gene	Annotation

**Global**	1:212101892:A:G	rs75615271	1	212101892	A	G	0.112	1.221	1.112	1.328	4.85E-08	RP11-552D8.1	non-coding intronic
	1:190390297:A:C	rs16832316	1	190390297	C	A	0.145	1.218	1.215	1.221	1.10E-06	BRINP3	intronic
	2:175200014:A:G	rs34372833	2	175200014	A	G	0.251	1.205	1.200	1.210	3.66E-06	SP9	intronic
	4:100475847:C:T	rs72906793	4	100475847	T	C	0.391	1.146	1.141	1.151	3.67E-06	TRMT10A	intronic,3downstream
	12:66707644:G:T	rs12301608	12	66707644	T	G	0.27	1.109	1.098	1.119	4.21E-06	HELB	intronic

**Anterior**	1:212101892:A:G	rs75615271	1	212101892	A	G	0.126	1.161	1.082	1.248	2.19E-07	RP11-552D8.1	non-coding intronic
	1:67671368:A:T	rs79148417	1	67671368	T	A	0.09	1.154	1.069	1.238	2.69E-07	IL23R. C1orf141	5upstream,intronic
	8:11388764:A:G	rs2249259	8	11388764	A	G	0.581	1.132	1.050	1.215	8.83E-06	BLK	intronic
	15:59443410:A:G	rs111507056	15	59443410	A	G	0.466	1.125	1.046	1.203	9.47E-06	MYO1E	intronic
	1:226032677:A:G	rs4149228	1	226032677	G	A	0.146	1.114	1.034	1.195	9.87E-06	EPHX1	non-coding intronic

**Posterior**	3:872733:C:T	rs62238282	3	872733	T	C	0.425	1.225	1.153	1.308	2.25E-07	AC090044.1	non-coding intronic
	8:142292415:A:C	rs114966000	8	142292415	A	C	0.166	1.218	1.141	1.294	4.16E-07	SLC45A4	non-coding intronic,intronic
	2:16584490:C:T	rs78164946	2	16584490	T	C	0.411	1.215	1.143	1.288	5.52E-07	AC010880.1	non-coding intronic
	10:30125824:C:T	rs72806925	10	30125824	T	C	0.085	1.214	1.136	1.292	6.04E-07	SVIL	non-coding intronic
	22:28296928:A:G	rs141874957	22	28296928	G	A	0.131	1.146	1.071	1.220	8.03E-07	PITPNB	non-coding intronic,intronic

**Table 3:** Mapped genes

Asymptomatic ILAS	Gene	Symbol	entrezID	chr	start	end	pLI	ncRVIS	IndSigSNPs

**Global**	ENSG00000065600	TMEM206	55248	1	212537273	212588243	8.01E-06	−0.130673507	rs74872539
	ENSG00000109270	LAMTOR3	8649	4	100799493	100815647	0.680579445	0.00466647	rs72906793
	ENSG00000117650	NEK2	4751	1	211836114	211848960	8.72E-07	−0.63820179	rs74872539
	ENSG00000123684	LPGAT1	9926	1	211916799	212004114	0.967547481	1.854780042	rs74872539
	ENSG00000128656	CHN1	1123	2	175664091	175870097	0.83742443	-	rs34372833
	ENSG00000134444	KIAA1468	57614	18	59854491	59974355	0.99999956	−0.173206824	rs74803525
	ENSG00000138823	MTTP	4547	4	100484918	100545156	0.012583367	0.759771402	rs72906793
	ENSG00000141655	TNFRSF11A	8792	18	59992520	60058516	0.879917354	2.378759843	rs74803525
	ENSG00000141664	ZCCHC2	54877	18	60190240	60254942	0.305659357	−0.580417645	rs74803525
	ENSG00000143476	DTL	51514	1	212208919	212280742	0.007013382	0.365079452	rs74872539
	ENSG00000143493	INTS7	25896	1	212113741	212208884	0.860980155	0.732840227	rs74872539
	ENSG00000144306	SCRN3	79634	2	175260458	175294303	1.32E-06	0.557980338	rs34372833
	ENSG00000145331	TRMT10A	93587	4	100467866	100485189	2.84E-05	−0.268447548	rs72906793
	ENSG00000162670	BRINP3	339479	1	190066792	190446759	0.003759093	−0.040916772	rs16832316
	ENSG00000185046	ANKS1B	56899	12	99120235	100378432	0.992771668	-	rs10777950
	ENSG00000197563	PIGN	23556	18	59710800	59854351	9.02E-16	−0.03863799	rs74803525
	ENSG00000217236	SP9	100131390	2	175199674	175203220	0.498147938	-	rs34372833
	ENSG00000268241	AC018470.1	-	2	175200601	175202151	-	-	rs34372833

**Anterior**	ENSG00000065600	TMEM206	55248	1	212537273	212588243	8.01E-06	−0.130673507	rs74872539
	ENSG00000117650	NEK2	4751	1	211836114	211848960	8.72E-07	−0.63820179	rs74872539
	ENSG00000123684	LPGAT1	9926	1	211916799	212004114	0.967547481	1.854780042	rs74872539
	ENSG00000143476	DTL	51514	1	212208919	212280742	0.007013382	0.365079452	rs74872539
	ENSG00000143493	INTS7	25896	1	212113741	212208884	0.860980155	0.732840227	rs74872539
	ENSG00000143742	SRP9	6726	1	225965531	225978168	0.308171839	−0.229774838	rs4149228
	ENSG00000143751	SDE2	163859	1	226170403	226187032	5.17E-07	−1.619069595	rs4149228
	ENSG00000143768	LEFTY2	10637	1	226124298	226129189	0.122897476	−0.647907632	rs4149228
	ENSG00000143815	LBR	3930	1	225589204	225616627	0.997025227	−0.491760231	rs4149228
	ENSG00000154380	E-H	55740	1	225674537	225840844	0.995700037	−0.004988799	rs4149228
	ENSG00000157483	MYO1E	4643	15	59427113	59665099	0.047977893	0.925227799	rs111507056
	ENSG00000196187	TMEM63A	9725	1	226033237	226070069	6.11E-05	0.74106925	rs4149228
	ENSG00000243709	LEFTY1	10637	1	226073982	226099082	6.88E-05	0.426227262	rs4149228
	ENSG00000255835	RP4-559A3.7	-	1	226074396	226111978	-	-	rs4149228

**Posterior**	ENSG00000076685	NT5C2	22978	10	104845940	104953056	0.052197863	0.04832858	rs76325870
	ENSG00000077150	NFKB2	4791	10	104153867	104162281	0.999696458	−0.681516998	rs76325870
	ENSG00000129214	SHBG	6462	17	7517382	7536700	0.000188842	0.239750308	rs11658781
	ENSG00000129245	FXR2	9513	17	7494548	7518189	0.999770109	-	rs11658781
	ENSG00000138172	CALHM2	51063	10	105206543	105212660	5.29E-07	−0.689941205	rs76325870
	ENSG00000148795	CYP17A1	1586	10	104590288	104597290	0.035030608	0.086681977	rs76325870
	ENSG00000148798	I-	9118	10	105036920	105050108	0.292917015	−0.860661456	rs76325870
	ENSG00000148842	CNNM2	54805	10	104678050	104849978	0.994106431	−0.511524122	rs76325870
	ENSG00000148843	PDCD11	22984	10	105156405	105206049	0.468448698	−0.805378431	rs76325870
	ENSG00000150782	IL18	3606	11	112013974	112034840	0.102774109	0.711064152	rs2103173
	ENSG00000156374	PCGF6	84108	10	105062553	1.05E+08	0.000251716	−0.491784974	rs76325870
	ENSG00000161955	TNFSF13	8741	17	7461609	7.46E+06	0.877768013	−0.927188875	rs11658781
	ENSG00000161956	SENP3	26168	17	7465192	7.48E+06	0.99599542	-	rs11658781
	ENSG00000161958	FGF11	2256	17	7341592	7.35E+06	0.068195836	−0.090451681	rs11658781
	ENSG00000164815	ORC5	5001	7	103766788	1.04E+08	2.29E-05	1.088750131	rs17159448
	ENSG00000166272	WBP1L	54838	10	104503727	1.05E+08	0.909387314	0.614640467	rs76325870
	ENSG00000168702	LRP1B	53353	2	140988992	142889270	0.99999919	−0.165250943	rs6722921
	ENSG00000168779	SHOX2	6474	3	157814948	1.58E+08	0.085211228	0.382488989	rs9866741
	ENSG00000168827	GFM1	85476	3	158362067	1.58E+08	1.66E-06	0.025437529	rs9866741
	ENSG00000170175	CHRNB1	1140	17	7348380	7.36E+06	4.34E-06	−0.373259936	rs11658781
	ENSG00000171206	TRIM8	81603	10	104404253	1.04E+08	0.992012341	−0.521290389	rs76325870
	ENSG00000174282	ZBTB4	57659	17	7362685	7.39E+06	0.998490203	−0.565704482	rs11658781
	ENSG00000178053	MLF1	4291	3	158288952	1.58E+08	1.28E-06	−0.002234274	rs9866741
	ENSG00000181222	POLR2A	5430	17	7387685	7.42E+06	0.999999942	0.931618008	rs11658781
	ENSG00000181284	TMEM102	284114	17	7338762	7.34E+06	-	0.016672853	rs11658781
	ENSG00000182141	ZNF708	7562	19	21473962	21512227	4.18E-09	1.095220078	rs112846999
	ENSG00000182224	CYB5D1	124637	17	7761064	7.77E+06	4.58E-05	−1.966049949	rs11658781
	ENSG00000185933	CALHM1	255022	10	105213144	1.05E+08	9.36E-05	0.209560501	rs76325870
	ENSG00000187210	GCNT1	2650	9	79034752	7.91E+07	0.066576637	0.748297206	rs17721472
	ENSG00000197020	ZNF100	163227	19	21905568	2.20E+07	7.10E-13	1.454526972	rs112846999
	ENSG00000197580	BCO2	83875	11	112046190	112095422	1.83E-13	−0.120152033	rs2103173
	ENSG00000198521	ZNF43	7594	19	21987752	22034927	2.15E-08	0.35246967	rs112846999
	ENSG00000214435	AS3MT	57412	10	104629273	104661656	5.09E-07	0.726881178	rs76325870
	ENSG00000235376	RPEL1	729020	10	105005644	105007773	0.002399072	-	rs76325870
	ENSG00000239697	TNFSF12	407977	17	7452208	7464925	0.997954015	−0.448531377	rs11658781
	ENSG00000259224	SLC35G6	643664	17	7384721	7386383	0.000990015	0.390339512	rs11658781
	ENSG00000268503	AC007421.1	-	17	7517264	7517427	-	-	rs11658781

**Table 4:** MAGMA pathway analysis

Asymptomatic ILAS	Gene Set	N genes	Beta	SE	P value	Bonferroni adjusted P value

**Global**	GOBP_NEGATIVE_REGULATION_OF_MRNA_METABOLIC_PROCESS	84	0.37604	0.089088	1.22E-05	0.207888784
	GOBP_IMMUNOGLOBULIN_V_D_J_RECOMBINATION	8	1.2478	0.29988	1.59E-05	0.270836475
	GOBP_TELOMERE_CAPPING	40	0.47364	0.11524	1.99E-05	0.337977244
	GOBP_SOMATIC_DIVERSIFICATION_OF_IMMUNE_RECEPTORS	78	0.37631	0.091855	2.10E-05	0.35788723
	GOMF_HMG_BOX_DOMAIN_BINDING	14	0.96363	0.23613	2.25E-05	0.383049108
	GOCC_DNA_REPAIR_COMPLEX	22	0.59916	0.14794	2.57E-05	0.43714713
	GOBP_V_D_J_RECOMBINATION	19	0.7024	0.17996	4.77E-05	0.81057035
	KEGG_NON_HOMOLOGOUS_END_JOINING	13	0.74362	0.19759	8.41E-05	1
	KAUFFMANN_MELANOMA_RELAPSE_DN	6	1.1606	0.31067	9.38E-05	1
	GOBP_SOMATIC_DIVERSIFICATION_OF_IMMUNOGLOBULINS	66	0.37845	0.10289	0.0001179	1

**Anterior**	KEGG_NON_HOMOLOGOUS_END_JOINING	13	0.91476	0.19849	2.04E-06	0.03471673
	CONRAD_GERMLINE_STEM_CELL	9	1.0626	0.2405	5.00E-06	0.085094525
	GOBP_MITOCHONDRIAL_ELECTRON_TRANSPORT_UBIQUINOL_TO_CYTOCHROME_C	10	0.96122	0.23832	2.76E-05	0.469637696
	GOBP_POSITIVE_REGULATION_OF_ENDOTHELIAL_CELL_MATRIX_ADHESION_VIA_FIBRO NECTIN	5	1.2922	0.32444	3.42E-05	0.58146897
	GOMF_SINGLE_STRANDED_DNA_ENDODEOXYRIBONUCLEASE_ACTIVITY	10	0.85128	0.22238	6.48E-05	1
	GOBP_REGULATION_OF_BICELLULAR_TIGHT_JUNCTION_ASSEMBLY	20	0.65589	0.17217	6.99E-05	1
	WP_DNA_REPAIR_PATHWAYS_FULL_NETWORK	114	0.25849	0.069082	9.17E-05	1
	GOCC_MITOCHONDRIAL_RESPIRATORY_CHAIN_COMPLEX_III	9	0.96756	0.26231	0.0001131	1
	GOBP_REGULATION_OF_DNA_DAMAGE_RESPONSE_SIGNAL_TRANSDUCTION_BY_P53_C LASS_MEDIATOR_RESULTING_IN_TRANSCRIPTION_OF_P21_CLASS_MEDIATOR	6	1.1276	0.30955	0.0001352	1
	GOCC_PLATELET_DENSE_GRANULE_LUMEN	14	0.68594	0.18925	0.0001452	1

**Posterior**	GOCC_MICROTUBULE_BUNDLE	6	1.2049	0.26099	1.96E-06	0.033412216
	GOCC_CATALYTIC_STEP_1_SPLICEOSOME	10	1.1587	0.27096	9.56E-06	0.162505286
	GOBP_REGULATION_OF_CHOLESTEROL_BIOSYNTHETIC_PROCESS	19	0.72552	0.18063	2.97E-05	0.504295924
	GOCC_KATANIN_COMPLEX	5	1.7731	0.44551	3.46E-05	0.5887131
	GOCC_BLOOD_MICROPARTICLE	105	0.31743	0.08329	6.94E-05	1
	GOCC_EXON_EXON_JUNCTION_COMPLEX	9	1.0436	0.2752	7.49E-05	1
	GOBP_BLOOD_VESSEL_MATURATION	8	1.1217	0.29942	9.00E-05	1
	GOCC_PHAGOCYTIC_CUP	28	0.57012	0.15524	0.0001205	1
	GOBP_NEGATIVE_REGULATION_OF_LOW_DENSITY_LIPOPROTEIN_RECEPTOR_ACTIVITY	7	1.0035	0.28517	0.0002173	1
	GOBP_REGULATION_OF_MEMBRANE_LIPID_DISTRIBUTION	59	0.39946	0.11503	0.0002584	1

**Table 5:** Geneset GO analysis

GENE	Symbol	CHR	START	STOP	N_SNPS	P

**KEGG non-homologous end-joining pathway associated with anterior ILAS**						
ENSG00000079246	XRCC5	2	216970187	217073026	137	0.53086
ENSG00000187736	NHEJ1	2	219938039	220027587	96	0.12963
ENSG00000152422	XRCC4	5	82371317	82651606	191	0.011586
ENSG00000113522	RAD50	5	131889711	131982313	41	0.11128
ENSG00000122678	POLM	7	44109846	44124139	11	0.039869
ENSG00000253729	PRKDC	8	48683669	48874743	34	0.66164
ENSG00000152457	DCLRE1C	10	14937358	14998431	38	0.001697
ENSG00000107447	DNTT	10	98062085	98100321	34	0.49185
ENSG00000166169	POLL	10	103336639	103350027	7	0.1911
ENSG00000168496	FEN1	11	61558109	61566716	1	0.4407
ENSG00000020922	MRE11A	11	94150895	94229074	39	0.14837
ENSG00000174405	LIG4	13	108857787	108872716	15	0.18778
ENSG00000196419	XRCC6	22	42015123	42062044	5	0.90636
**GOCC_MICROTUBULE_BUNDLE asssociated posterior ILAS**						
ENSG00000171368	TPPP	5	658883	695510	36	0.13995
ENSG00000137343	ATAT1	6	30592619	30616600	12	0.20469
ENSG00000072518	MARK2	11	63604400	63680491	28	0.34922
ENSG00000137497	NUMA1	11	71711910	71793739	11	0.041269
ENSG00000159713	TPPP3	16	67421712	67429438	1	0.7629
ENSG00000168502	SOGA2	18	8703659	8834776	117	0.21855

**Table 6:** Gene enrichment analysis

Category	GeneSet	N_genes	N_overlap_genes	p	adjP	genes

**Global ILAS**						
Positional_gene_sets	chr1q32	141	5	1.34E-07	4.02E-05	NEK2,LPGAT1,INTS7,DTL,TMEM206
Positional_gene_sets	chr4q23	19	3	6.08E-07	8.38E-05	TRMT10A,MTTP,LAMTOR3
Positional_gene_sets	chr18q21	83	4	8.38E-07	8.38E-05	PIGN,KIAA1468,TNFRSF11A,ZCCHC2
Positional_gene_sets	chr2q31	79	3	4.80E-05	0.003600179	SP9,SCRN3,CHN1
**Anterior ILAS**						
Positional_gene_sets	chr1q42	89	6	9.12E-12	2.74E-09	LBR, ENAH, SRP9, TMEM63A, LEFTY1, LEFTY2, SDE2
Positional_gene_sets	chr1q32	141	5	1.77E-08	2.65E-06	NEK2, LPGAT1, INTS7, DTL, TMEM206
Curated_gene_sets	PATIL_LIVER_CANCER	619	7	2.99E-08	1.94E-04	NEK2, LPGAT1, INTS7, DTL, TMEM206, LBR, ENAH
Curated_gene_sets	OISHI_CHOLANGIOMA_STEM_CELL_LIKE_UP	329	5	1.20E-06	3.90E-03	LPGAT1, INTS7, LBR, ENAH, SRP9
Curated_gene_sets	ZHOU_CELL_CYCLE_GENES_IN_IR_RESPONSE_6HR	82	3	1.76E-05	3.39E-02	NEK2, DTL, LBR
Curated_gene_sets	LOPEZ_MESOTHELIOMA_SURVIVAL_DN	11	2	2.09E-05	3.39E-02	NEK2, ENAH
Immunologic signatures	GSE30962_ACUTE_VS_CHRONIC_LCMV_PRIMARY_INF_CD8_TCELL_DN	193	4	5.21E-06	2.72E-02	NEK2, LPGAT1, DTL, MYO1E
**Posterior ILAS**						
Positional_gene_sets	chr10q24	117	13	2.48E-20	7.44E-18	NFKB2, TRIM8, WBP1L, CYP17A1, AS3MT, CNNM2, NT5C2, RPEL1, INA, PCGF6, PDCD11, CALHM2, CALHM1
Positional_gene_sets	chr17p13	227	12	7.86E-15	1.18E-12	TMEM102, FGF11, CHRNB1, ZBTB4, SLC35G6, POLR2A, TNFSF12, TNFSF12-TNFSF13, TNFSF13, SENP3, FXR2, SHBG, CYB5D1
Positional_gene_sets	chr19p12	29	3	2.35E-05	2.35E-03	ZNF708, ZNF100, ZNF43
Positional_gene_sets	chr3q25	66	3	2.80E-04	2.10E-02	SHOX2, MLF1, GFM1
GWAS catalog reported genes	Brain morphology (MOSTest)	1049	20	2.73E-16	1.21E-12	TRIM8, WBP1L, CYP17A1, AS3MT, CNNM2, NT5C2, INA, PCGF6, TMEM102, FGF11, CHRNB1, ZBTB4, SLC35G6, POLR2A, TNFSF12, TNFSF12-TNFSF13, TNFSF13, SENP3, FXR2, SHBG, CYB5D1
GWAS catalog reported genes	Response to cognitive-behavioural therapy in major depressive disorder	31	8	6.51E-16	1.44E-12	CNNM2, NT5C2, RPEL1, INA, PCGF6, PDCD11, CALHM2, CALHM1
GWAS catalog reported genes	Autism spectrum disorder or schizophrenia	355	10	9.22E-10	1.36E-06	TRIM8, WBP1L, CYP17A1, AS3MT, CNNM2, NT5C2, RPEL1, INA, PCGF6, PDCD11
GWAS catalog reported genes	Waist-to-hip ratio adjusted for BMI (age >50)	178	7	4.31E-08	4.76E-05	NFKB2, TRIM8, WBP1L, CYP17A1, AS3MT, CNNM2, NT5C2
GWAS catalog reported genes	Mean arterial pressure	109	6	5.94E-08	5.26E-05	WBP1L, CYP17A1, AS3MT, CNNM2, NT5C2, RPEL1
GWAS catalog reported genes	Schizophrenia	613	10	1.64E-07	1.21E-04	TRIM8, WBP1L, CYP17A1, AS3MT, CNNM2, NT5C2, RPEL1, INA, PCGF6, PDCD11
GWAS catalog reported genes	White matter lesion progression	9	3	5.54E-07	3.50E-04	AS3MT, CNNM2, NT5C2
GWAS catalog reported genes	IgM levels	13	3	1.88E-06	1.04E-03	TNFSF13, SENP3, FXR2
GWAS catalog reported genes	Arsenic metabolism	2	2	3.64E-06	1.79E-03	AS3MT, CNNM2
GWAS catalog reported genes	Waist-to-hip ratio adjusted for BMI	695	9	5.12E-06	2.26E-03	NFKB2, TRIM8, WBP1L, CYP17A1, AS3MT, CNNM2, NT5C2, POLR2A, SHBG
GWAS catalog reported genes	Refractive error	1381	12	6.24E-06	2.51E-03	NFKB2, TRIM8, TMEM102, FGF11, CHRNB1, ZBTB4, SLC35G6, POLR2A, TNFSF12, TNFSF12-TNFSF13, SENP3, FXR2, SHBG
GWAS catalog reported genes	Corpus callosum posterior volume	3	2	1.09E-05	4.02E-03	ZBTB4, SLC35G6
GWAS catalog reported genes	Blood pressure	74	4	1.23E-05	4.20E-03	CYP17A1, AS3MT, CNNM2, NT5C2
GWAS catalog reported genes	Mean arterial pressure (long-term average)	4	2	2.18E-05	6.03E-03	WBP1L, AS3MT
GWAS catalog reported genes	Systolic blood pressure (long-term average)	4	2	2.18E-05	6.03E-03	WBP1L, NT5C2
GWAS catalog reported genes	Diastolic blood pressure (long-term average)	4	2	2.18E-05	6.03E-03	WBP1L, AS3MT
GWAS catalog reported genes	Autism spectrum disorder	39	3	5.80E-05	1.51E-02	AS3MT, CNNM2, NT5C2
GWAS catalog reported genes	Hypertension	113	4	6.54E-05	1.61E-02	WBP1L, CYP17A1, CNNM2, NT5C2
GWAS catalog reported genes	Interleukin-18 levels	7	2	7.60E-05	1.77E-02	IL18, BCO2
GWAS catalog reported genes	Pulse pressure	560	7	8.42E-05	1.86E-02	SHOX2, TRIM8, WBP1L, CYP17A1, CNNM2, NT5C2, RPEL1
GWAS catalog reported genes	Coronary heart disease	55	3	1.63E-04	3.43E-02	CYP17A1, CNNM2, NT5C2
GWAS catalog reported genes	Prostate cancer	273	5	1.70E-04	3.43E-02	TRIM8, WBP1L, CYP17A1, AS3MT, CNNM2
GWAS catalog reported genes	Waist-to-hip ratio adjusted for BMI x sex x age interaction (4df test)	299	5	2.60E-04	4.97E-02	NFKB2, TRIM8, WBP1L, CYP17A1, AS3MT
GWAS catalog reported genes	Systolic blood pressure	676	7	2.70E-04	4.97E-02	MLF1, WBP1L, CYP17A1, AS3MT, CNNM2, NT5C2, RPEL1

**Table 7:** Co-localization of ILAS with Tissue-Specific Gene Expression

Asymptomatic ILAS	uniqID	db	tissue	gene	symbol	chr	pos	Risk Allele	p

**Global**	18:59793695:A:C	GTEx/v8	Brain_Cortex	ENSG00000197563	PIGN	18	59793695	A	9.75362E-14
	18:59779709:A:G	GTEx/v8	Brain_Cortex	ENSG00000197563	PIGN	18	59779709	G	2.67214E-13
	18:59780393:A:T	GTEx/v8	Brain_Cortex	ENSG00000197563	PIGN	18	59780393	A	2.67214E-13
	18:59789376:C:T	GTEx/v8	Brain_Cortex	ENSG00000197563	PIGN	18	59789376	C	2.67214E-13
	18:59794258:A:G	GTEx/v8	Brain_Cortex	ENSG00000197563	PIGN	18	59794258	A	2.67214E-13
	18:59818637:A:G	GTEx/v8	Brain_Cortex	ENSG00000197563	PIGN	18	59818637	A	2.67214E-13
	18:59819308:A:G	GTEx/v8	Brain_Cortex	ENSG00000197563	PIGN	18	59819308	G	2.67214E-13
	18:59845510:C:T	GTEx/v8	Brain_Cortex	ENSG00000197563	PIGN	18	59845510	C	2.67214E-13
	18:59896321:A:G	GTEx/v8	Brain_Cortex	ENSG00000197563	PIGN	18	59896321	A	2.75616E-13
	18:59908592:C:T	GTEx/v8	Brain_Cortex	ENSG00000197563	PIGN	18	59908592	T	2.75616E-13
	18:59919077:C:T	GTEx/v8	Brain_Cortex	ENSG00000197563	PIGN	18	59919077	C	2.75616E-13
	18:59847898:C:T	GTEx/v8	Brain_Cortex	ENSG00000197563	PIGN	18	59847898	T	3.02E-13
	18:59945172:A:G	GTEx/v8	Brain_Cortex	ENSG00000197563	PIGN	18	59945172	A	1.00835E-10
	18:59976217:A:G	GTEx/v8	Brain_Cortex	ENSG00000197563	PIGN	18	59976217	G	1.00835E-10
	18:59896321:A:G	GTEx/v8	Brain_Caudate_basal_ganglia	ENSG00000197563	PIGN	18	59896321	A	1.06218E-10
	18:59908592:C:T	GTEx/v8	Brain_Caudate_basal_ganglia	ENSG00000197563	PIGN	18	59908592	T	1.06218E-10
	18:59919077:C:T	GTEx/v8	Brain_Caudate_basal_ganglia	ENSG00000197563	PIGN	18	59919077	C	1.06218E-10
	4:100467808:A:T	GTEx/v8	Artery_Tibial	ENSG00000145331	TRMT10A	4	100467808	A	1.1766E-10
	4:100486295:A:G	GTEx/v8	Artery_Tibial	ENSG00000145331	TRMT10A	4	100486295	G	1.1766E-10
	4:100541636:A:T	GTEx/v8	Artery_Tibial	ENSG00000145331	TRMT10A	4	100541636	T	1.1766E-10
	4:100556160:C:T	GTEx/v8	Artery_Tibial	ENSG00000145331	TRMT10A	4	100556160	C	1.1766E-10
	18:59845510:C:T	GTEx/v8	Brain_Caudate_basal_ganglia	ENSG00000197563	PIGN	18	59845510	C	1.93205E-10
	18:59847898:C:T	GTEx/v8	Brain_Caudate_basal_ganglia	ENSG00000197563	PIGN	18	59847898	T	1.93205E-10
	18:59779709:A:G	GTEx/v8	Brain_Caudate_basal_ganglia	ENSG00000197563	PIGN	18	59779709	G	2.01185E-10
	18:59780393:A:T	GTEx/v8	Brain_Caudate_basal_ganglia	ENSG00000197563	PIGN	18	59780393	A	2.01185E-10
	18:59789376:C:T	GTEx/v8	Brain_Caudate_basal_ganglia	ENSG00000197563	PIGN	18	59789376	C	2.01185E-10
	18:59794258:A:G	GTEx/v8	Brain_Caudate_basal_ganglia	ENSG00000197563	PIGN	18	59794258	A	2.01185E-10
	18:59818637:A:G	GTEx/v8	Brain_Caudate_basal_ganglia	ENSG00000197563	PIGN	18	59818637	A	2.01185E-10
	18:59819308:A:G	GTEx/v8	Brain_Caudate_basal_ganglia	ENSG00000197563	PIGN	18	59819308	G	2.01185E-10
	18:59982808:C:T	GTEx/v8	Brain_Cortex	ENSG00000197563	PIGN	18	59982808	C	4.04565E-10
	18:59793695:A:C	GTEx/v8	Brain_Caudate_basal_ganglia	ENSG00000197563	PIGN	18	59793695	A	1.15741E-09
	18:59919077:C:T	GTEx/v8	Cells_Cultured_fibroblasts	ENSG00000197563	PIGN	18	59919077	C	1.13647E-08
	18:59908592:C:T	GTEx/v8	Cells_Cultured_fibroblasts	ENSG00000197563	PIGN	18	59908592	T	1.91141E-08
	18:59896321:A:G	GTEx/v8	Cells_Cultured_fibroblasts	ENSG00000197563	PIGN	18	59896321	A	2.57177E-08
	4:100475847:C:T	GTEx/v8	Artery_Tibial	ENSG00000145331	TRMT10A	4	100475847	T	3.13553E-08
	4:100479983:A:C	GTEx/v8	Artery_Tibial	ENSG00000145331	TRMT10A	4	100479983	C	3.13553E-08
	4:100487712:A:G	GTEx/v8	Artery_Tibial	ENSG00000145331	TRMT10A	4	100487712	A	3.13553E-08
	4:100520584:A:G	GTEx/v8	Artery_Tibial	ENSG00000145331	TRMT10A	4	100520584	G	3.13553E-08
	4:100506372:A:G	GTEx/v8	Artery_Tibial	ENSG00000145331	TRMT10A	4	100506372	A	3.76133E-08
	18:59976217:A:G	GTEx/v8	Cells_Cultured_fibroblasts	ENSG00000197563	PIGN	18	59976217	G	5.67966E-08
	18:59982808:C:T	GTEx/v8	Cells_Cultured_fibroblasts	ENSG00000197563	PIGN	18	59982808	C	5.67966E-08
	4:100541413:A:G	GTEx/v8	Artery_Tibial	ENSG00000145331	TRMT10A	4	100541413	A	6.2968E-08
	18:59945172:A:G	GTEx/v8	Cells_Cultured_fibroblasts	ENSG00000197563	PIGN	18	59945172	A	7.68673E-08
	4:100525515:G:T	GTEx/v8	Artery_Tibial	ENSG00000145331	TRMT10A	4	100525515	G	1.17706E-07
	4:100527074:C:T	GTEx/v8	Artery_Tibial	ENSG00000145331	TRMT10A	4	100527074	C	1.17706E-07
	18:59945172:A:G	GTEx/v8	Brain_Caudate_basal_ganglia	ENSG00000197563	PIGN	18	59945172	A	1.89824E-07
	18:59818637:A:G	GTEx/v8	Brain_Frontal_Cortex_BA9	ENSG00000197563	PIGN	18	59818637	A	2.08408E-07
	18:59819308:A:G	GTEx/v8	Brain_Frontal_Cortex_BA9	ENSG00000197563	PIGN	18	59819308	G	2.08408E-07
	18:59847898:C:T	GTEx/v8	Brain_Frontal_Cortex_BA9	ENSG00000197563	PIGN	18	59847898	T	2.08763E-07
	18:59779709:A:G	GTEx/v8	Brain_Frontal_Cortex_BA9	ENSG00000197563	PIGN	18	59779709	G	2.30019E-07
	18:59780393:A:T	GTEx/v8	Brain_Frontal_Cortex_BA9	ENSG00000197563	PIGN	18	59780393	A	2.30019E-07
	18:59789376:C:T	GTEx/v8	Brain_Frontal_Cortex_BA9	ENSG00000197563	PIGN	18	59789376	C	2.30019E-07
	18:59794258:A:G	GTEx/v8	Brain_Frontal_Cortex_BA9	ENSG00000197563	PIGN	18	59794258	A	2.30019E-07
	18:59793695:A:C	GTEx/v8	Breast_Mammary_Tissue	ENSG00000134444	KIAA1468	18	59793695	A	2.90192E-07
	18:59845510:C:T	GTEx/v8	Brain_Frontal_Cortex_BA9	ENSG00000197563	PIGN	18	59845510	C	3.00774E-07
	18:59896321:A:G	GTEx/v8	Breast_Mammary_Tissue	ENSG00000134444	KIAA1468	18	59896321	A	3.11111E-07
	18:59976217:A:G	GTEx/v8	Brain_Caudate_basal_ganglia	ENSG00000197563	PIGN	18	59976217	G	3.15245E-07
	18:59982808:C:T	GTEx/v8	Brain_Caudate_basal_ganglia	ENSG00000197563	PIGN	18	59982808	C	3.15245E-07
	18:59818637:A:G	GTEx/v8	Breast_Mammary_Tissue	ENSG00000134444	KIAA1468	18	59818637	A	3.49238E-07
	18:59819308:A:G	GTEx/v8	Breast_Mammary_Tissue	ENSG00000134444	KIAA1468	18	59819308	G	3.49238E-07
	18:59845510:C:T	GTEx/v8	Breast_Mammary_Tissue	ENSG00000134444	KIAA1468	18	59845510	C	3.49238E-07
	18:59847898:C:T	GTEx/v8	Breast_Mammary_Tissue	ENSG00000134444	KIAA1468	18	59847898	T	3.49238E-07
	18:59896321:A:G	GTEx/v8	Brain_Frontal_Cortex_BA9	ENSG00000197563	PIGN	18	59896321	A	4.26403E-07
	18:59908592:C:T	GTEx/v8	Brain_Frontal_Cortex_BA9	ENSG00000197563	PIGN	18	59908592	T	4.26403E-07
	18:59919077:C:T	GTEx/v8	Brain_Frontal_Cortex_BA9	ENSG00000197563	PIGN	18	59919077	C	4.26403E-07
	18:59779709:A:G	GTEx/v8	Brain_Putamen_basal_ganglia	ENSG00000197563	PIGN	18	59779709	G	4.50731E-07
	18:59780393:A:T	GTEx/v8	Brain_Putamen_basal_ganglia	ENSG00000197563	PIGN	18	59780393	A	4.50731E-07
	18:59789376:C:T	GTEx/v8	Brain_Putamen_basal_ganglia	ENSG00000197563	PIGN	18	59789376	C	4.50731E-07
	18:59794258:A:G	GTEx/v8	Brain_Putamen_basal_ganglia	ENSG00000197563	PIGN	18	59794258	A	4.50731E-07
	18:59945172:A:G	GTEx/v8	Brain_Cerebellum	ENSG00000134444	KIAA1468	18	59945172	A	5.9136E-07
	18:59976217:A:G	GTEx/v8	Brain_Cerebellum	ENSG00000134444	KIAA1468	18	59976217	G	5.9136E-07
	18:59982808:C:T	GTEx/v8	Brain_Cerebellum	ENSG00000134444	KIAA1468	18	59982808	C	5.9136E-07
	2:175200014:A:G	GTEx/v8	Muscle_Skeletal	ENSG00000144306	SCRN3	2	175200014	A	6.18328E-07
	18:59818637:A:G	GTEx/v8	Brain_Putamen_basal_ganglia	ENSG00000197563	PIGN	18	59818637	A	6.36515E-07
	18:59819308:A:G	GTEx/v8	Brain_Putamen_basal_ganglia	ENSG00000197563	PIGN	18	59819308	G	6.36515E-07
	18:59845510:C:T	GTEx/v8	Brain_Putamen_basal_ganglia	ENSG00000197563	PIGN	18	59845510	C	6.36515E-07
	18:59847898:C:T	GTEx/v8	Brain_Putamen_basal_ganglia	ENSG00000197563	PIGN	18	59847898	T	6.36515E-07
	18:59818637:A:G	GTEx/v8	Brain_Cerebellum	ENSG00000134444	KIAA1468	18	59818637	A	6.89215E-07
	18:59819308:A:G	GTEx/v8	Brain_Cerebellum	ENSG00000134444	KIAA1468	18	59819308	G	6.89215E-07
	18:59779709:A:G	GTEx/v8	Breast_Mammary_Tissue	ENSG00000134444	KIAA1468	18	59779709	G	7.82614E-07
	18:59780393:A:T	GTEx/v8	Breast_Mammary_Tissue	ENSG00000134444	KIAA1468	18	59780393	A	7.82614E-07
	18:59789376:C:T	GTEx/v8	Breast_Mammary_Tissue	ENSG00000134444	KIAA1468	18	59789376	C	7.82614E-07
	18:59794258:A:G	GTEx/v8	Breast_Mammary_Tissue	ENSG00000134444	KIAA1468	18	59794258	A	7.82614E-07
	18:59896321:A:G	GTEx/v8	Brain_Hippocampus	ENSG00000197563	PIGN	18	59896321	A	8.56812E-07
	18:59908592:C:T	GTEx/v8	Brain_Hippocampus	ENSG00000197563	PIGN	18	59908592	T	8.56812E-07
	18:59919077:C:T	GTEx/v8	Brain_Hippocampus	ENSG00000197563	PIGN	18	59919077	C	8.56812E-07
	18:59779709:A:G	GTEx/v8	Brain_Cerebellum	ENSG00000134444	KIAA1468	18	59779709	G	8.75256E-07
	18:59780393:A:T	GTEx/v8	Brain_Cerebellum	ENSG00000134444	KIAA1468	18	59780393	A	8.75256E-07
	18:59789376:C:T	GTEx/v8	Brain_Cerebellum	ENSG00000134444	KIAA1468	18	59789376	C	8.75256E-07
	18:59794258:A:G	GTEx/v8	Brain_Cerebellum	ENSG00000134444	KIAA1468	18	59794258	A	8.75256E-07
	18:59793695:A:C	GTEx/v8	Brain_Putamen_basal_ganglia	ENSG00000197563	PIGN	18	59793695	A	1.10212E-06
	18:59919077:C:T	GTEx/v8	Artery_Tibial	ENSG00000197563	PIGN	18	59919077	C	1.15871E-06
	18:59793695:A:C	GTEx/v8	Brain_Cerebellum	ENSG00000134444	KIAA1468	18	59793695	A	1.22053E-06
	18:59896321:A:G	GTEx/v8	Brain_Putamen_basal_ganglia	ENSG00000197563	PIGN	18	59896321	A	1.23261E-06
	18:59908592:C:T	GTEx/v8	Brain_Putamen_basal_ganglia	ENSG00000197563	PIGN	18	59908592	T	1.23261E-06
	18:59919077:C:T	GTEx/v8	Brain_Putamen_basal_ganglia	ENSG00000197563	PIGN	18	59919077	C	1.23261E-06
	18:59945172:A:G	GTEx/v8	Breast_Mammary_Tissue	ENSG00000134444	KIAA1468	18	59945172	A	1.46253E-06
	18:59976217:A:G	GTEx/v8	Breast_Mammary_Tissue	ENSG00000134444	KIAA1468	18	59976217	G	1.46253E-06
	18:59982808:C:T	GTEx/v8	Breast_Mammary_Tissue	ENSG00000134444	KIAA1468	18	59982808	C	1.46253E-06
	18:59793695:A:C	GTEx/v8	Brain_Spinal_cord_cervical_c-1	ENSG00000197563	PIGN	18	59793695	A	1.63882E-06
	18:59845510:C:T	GTEx/v8	Brain_Cerebellum	ENSG00000134444	KIAA1468	18	59845510	C	1.76795E-06
	18:59847898:C:T	GTEx/v8	Brain_Cerebellum	ENSG00000134444	KIAA1468	18	59847898	T	1.76795E-06
	18:59919077:C:T	GTEx/v8	Breast_Mammary_Tissue	ENSG00000134444	KIAA1468	18	59919077	C	1.83529E-06
	18:59896321:A:G	GTEx/v8	Lung	ENSG00000134444	KIAA1468	18	59896321	A	1.97537E-06
	18:59982808:C:T	GTEx/v8	Lung	ENSG00000134444	KIAA1468	18	59982808	C	2.25256E-06
	18:59945172:A:G	GTEx/v8	Brain_Cortex	ENSG00000134444	KIAA1468	18	59945172	A	2.33455E-06
	18:59976217:A:G	GTEx/v8	Brain_Cortex	ENSG00000134444	KIAA1468	18	59976217	G	2.33455E-06
	18:59908592:C:T	GTEx/v8	Whole_Blood	ENSG00000197563	PIGN	18	59908592	T	2.46232E-06
	18:59793695:A:C	GTEx/v8	Brain_Nucleus_accumbens_basal_ganglia	ENSG00000197563	PIGN	18	59793695	A	2.59458E-06
	18:59896321:A:G	GTEx/v8	Artery_Tibial	ENSG00000197563	PIGN	18	59896321	A	2.73953E-06
	4:100467808:A:T	GTEx/v8	Artery_Aorta	ENSG00000145331	TRMT10A	4	100467808	A	2.95628E-06
	4:100486295:A:G	GTEx/v8	Artery_Aorta	ENSG00000145331	TRMT10A	4	100486295	G	2.95628E-06
	4:100541636:A:T	GTEx/v8	Artery_Aorta	ENSG00000145331	TRMT10A	4	100541636	T	2.95628E-06
	18:59919077:C:T	GTEx/v8	Whole_Blood	ENSG00000197563	PIGN	18	59919077	C	3.14324E-06
	18:59896321:A:G	GTEx/v8	Brain_Spinal_cord_cervical_c-1	ENSG00000197563	PIGN	18	59896321	A	3.15615E-06
	18:59908592:C:T	GTEx/v8	Brain_Spinal_cord_cervical_c-1	ENSG00000197563	PIGN	18	59908592	T	3.15615E-06
	18:59919077:C:T	GTEx/v8	Brain_Spinal_cord_cervical_c-1	ENSG00000197563	PIGN	18	59919077	C	3.15615E-06
	18:59908592:C:T	GTEx/v8	Artery_Tibial	ENSG00000197563	PIGN	18	59908592	T	3.20602E-06
	18:59919077:C:T	GTEx/v8	Lung	ENSG00000134444	KIAA1468	18	59919077	C	3.39821E-06
	18:59945172:A:G	GTEx/v8	Brain_Hippocampus	ENSG00000197563	PIGN	18	59945172	A	3.62901E-06
	18:59976217:A:G	GTEx/v8	Brain_Hippocampus	ENSG00000197563	PIGN	18	59976217	G	3.62901E-06
	18:59982808:C:T	GTEx/v8	Brain_Hippocampus	ENSG00000197563	PIGN	18	59982808	C	3.62901E-06
	18:59779709:A:G	GTEx/v8	Brain_Hippocampus	ENSG00000197563	PIGN	18	59779709	G	3.65434E-06
	18:59780393:A:T	GTEx/v8	Brain_Hippocampus	ENSG00000197563	PIGN	18	59780393	A	3.65434E-06
	18:59789376:C:T	GTEx/v8	Brain_Hippocampus	ENSG00000197563	PIGN	18	59789376	C	3.65434E-06
	18:59794258:A:G	GTEx/v8	Brain_Hippocampus	ENSG00000197563	PIGN	18	59794258	A	3.65434E-06
	18:59896321:A:G	GTEx/v8	Brain_Cerebellum	ENSG00000134444	KIAA1468	18	59896321	A	3.76377E-06
	18:59908592:C:T	GTEx/v8	Brain_Cerebellum	ENSG00000134444	KIAA1468	18	59908592	T	3.76377E-06
	18:59919077:C:T	GTEx/v8	Brain_Cerebellum	ENSG00000134444	KIAA1468	18	59919077	C	3.76377E-06
	18:59945172:A:G	GTEx/v8	Brain_Frontal_Cortex_BA9	ENSG00000197563	PIGN	18	59945172	A	3.78334E-06
	18:59976217:A:G	GTEx/v8	Brain_Frontal_Cortex_BA9	ENSG00000197563	PIGN	18	59976217	G	3.78334E-06
	18:59982808:C:T	GTEx/v8	Brain_Frontal_Cortex_BA9	ENSG00000197563	PIGN	18	59982808	C	3.78334E-06
	18:59793695:A:C	GTEx/v8	Brain_Frontal_Cortex_BA9	ENSG00000197563	PIGN	18	59793695	A	3.83633E-06
	18:59818637:A:G	GTEx/v8	Brain_Hippocampus	ENSG00000197563	PIGN	18	59818637	A	3.83872E-06
	18:59819308:A:G	GTEx/v8	Brain_Hippocampus	ENSG00000197563	PIGN	18	59819308	G	3.83872E-06
	18:59945172:A:G	GTEx/v8	Lung	ENSG00000134444	KIAA1468	18	59945172	A	3.84412E-06
	18:59976217:A:G	GTEx/v8	Lung	ENSG00000134444	KIAA1468	18	59976217	G	3.84412E-06
	18:59908592:C:T	GTEx/v8	Breast_Mammary_Tissue	ENSG00000134444	KIAA1468	18	59908592	T	4.70908E-06
	18:59908592:C:T	GTEx/v8	Lung	ENSG00000134444	KIAA1468	18	59908592	T	4.94079E-06
	4:100556160:C:T	GTEx/v8	Artery_Aorta	ENSG00000145331	TRMT10A	4	100556160	C	4.9971E-06
	18:59779709:A:G	GTEx/v8	Brain_Nucleus_accumbens_basal_ganglia	ENSG00000197563	PIGN	18	59779709	G	5.11256E-06
	18:59780393:A:T	GTEx/v8	Brain_Nucleus_accumbens_basal_ganglia	ENSG00000197563	PIGN	18	59780393	A	5.11256E-06
	18:59789376:C:T	GTEx/v8	Brain_Nucleus_accumbens_basal_ganglia	ENSG00000197563	PIGN	18	59789376	C	5.11256E-06
	18:59794258:A:G	GTEx/v8	Brain_Nucleus_accumbens_basal_ganglia	ENSG00000197563	PIGN	18	59794258	A	5.11256E-06
	18:59896321:A:G	GTEx/v8	Whole_Blood	ENSG00000197563	PIGN	18	59896321	A	5.51454E-06
	18:59896321:A:G	GTEx/v8	Brain_Nucleus_accumbens_basal_ganglia	ENSG00000197563	PIGN	18	59896321	A	5.52581E-06
	18:59908592:C:T	GTEx/v8	Brain_Nucleus_accumbens_basal_ganglia	ENSG00000197563	PIGN	18	59908592	T	5.52581E-06
	18:59919077:C:T	GTEx/v8	Brain_Nucleus_accumbens_basal_ganglia	ENSG00000197563	PIGN	18	59919077	C	5.52581E-06
	18:59793695:A:C	GTEx/v8	Lung	ENSG00000134444	KIAA1468	18	59793695	A	5.83491E-06
	18:59896321:A:G	GTEx/v8	Brain_Cortex	ENSG00000134444	KIAA1468	18	59896321	A	6.00598E-06
	18:59908592:C:T	GTEx/v8	Brain_Cortex	ENSG00000134444	KIAA1468	18	59908592	T	6.00598E-06
	18:59919077:C:T	GTEx/v8	Brain_Cortex	ENSG00000134444	KIAA1468	18	59919077	C	6.00598E-06
	18:59818637:A:G	GTEx/v8	Brain_Nucleus_accumbens_basal_ganglia	ENSG00000197563	PIGN	18	59818637	A	6.21964E-06
	18:59819308:A:G	GTEx/v8	Brain_Nucleus_accumbens_basal_ganglia	ENSG00000197563	PIGN	18	59819308	G	6.21964E-06
	4:100525515:G:T	GTEx/v8	Heart_Left_Ventricle	ENSG00000145331	TRMT10A	4	100525515	G	6.44572E-06
	4:100527074:C:T	GTEx/v8	Heart_Left_Ventricle	ENSG00000145331	TRMT10A	4	100527074	C	6.44572E-06
	4:100541413:A:G	GTEx/v8	Heart_Left_Ventricle	ENSG00000145331	TRMT10A	4	100541413	A	6.44572E-06
	18:59779709:A:G	GTEx/v8	Brain_Spinal_cord_cervical_c-1	ENSG00000197563	PIGN	18	59779709	G	6.72715E-06
	18:59780393:A:T	GTEx/v8	Brain_Spinal_cord_cervical_c-1	ENSG00000197563	PIGN	18	59780393	A	6.72715E-06
	18:59789376:C:T	GTEx/v8	Brain_Spinal_cord_cervical_c-1	ENSG00000197563	PIGN	18	59789376	C	6.72715E-06
	18:59794258:A:G	GTEx/v8	Brain_Spinal_cord_cervical_c-1	ENSG00000197563	PIGN	18	59794258	A	6.72715E-06
	18:59779709:A:G	GTEx/v8	Lung	ENSG00000134444	KIAA1468	18	59779709	G	6.84043E-06
	18:59780393:A:T	GTEx/v8	Lung	ENSG00000134444	KIAA1468	18	59780393	A	6.84043E-06
	18:59789376:C:T	GTEx/v8	Lung	ENSG00000134444	KIAA1468	18	59789376	C	6.84043E-06
	18:59794258:A:G	GTEx/v8	Lung	ENSG00000134444	KIAA1468	18	59794258	A	6.84043E-06
	18:59845510:C:T	GTEx/v8	Lung	ENSG00000134444	KIAA1468	18	59845510	C	7.52551E-06
	18:59847898:C:T	GTEx/v8	Lung	ENSG00000134444	KIAA1468	18	59847898	T	7.52551E-06
	4:100525515:G:T	GTEx/v8	Artery_Aorta	ENSG00000138823	MTTP	4	100525515	G	8.05119E-06
	4:100527074:C:T	GTEx/v8	Artery_Aorta	ENSG00000138823	MTTP	4	100527074	C	8.05119E-06
	18:59818637:A:G	GTEx/v8	Lung	ENSG00000134444	KIAA1468	18	59818637	A	8.51482E-06
	18:59819308:A:G	GTEx/v8	Lung	ENSG00000134444	KIAA1468	18	59819308	G	8.51482E-06
	18:59945172:A:G	GTEx/v8	Artery_Tibial	ENSG00000197563	PIGN	18	59945172	A	9.82997E-06

**Anterior**	8:11392659:A:T	GTEx/v8	Whole_Blood	ENSG00000154319	FAM167A	8	11392659	A	3.42999E-22
	8:11389783:A:C	GTEx/v8	Whole_Blood	ENSG00000154319	FAM167A	8	11389783	A	6.39253E-21
	8:11389783:A:C	GTEx/v8	Cells_Cultured_fibroblasts	ENSG00000136573	BLK	8	11389783	A	6.89055E-19
	8:11392659:A:T	GTEx/v8	Whole_Blood	ENSG00000136573	BLK	8	11392659	A	5.35214E-18
	8:11389783:A:C	GTEx/v8	Whole_Blood	ENSG00000136573	BLK	8	11389783	A	6.79125E-18
	8:11392659:A:T	GTEx/v8	Cells_Cultured_fibroblasts	ENSG00000136573	BLK	8	11392659	A	1.30303E-17
	8:11389858:C:G	GTEx/v8	Whole_Blood	ENSG00000154319	FAM167A	8	11389858	G	2.57648E-17
	8:11389858:C:G	GTEx/v8	Cells_Cultured_fibroblasts	ENSG00000136573	BLK	8	11389858	G	2.67075E-17
	8:11389858:C:G	GTEx/v8	Whole_Blood	ENSG00000136573	BLK	8	11389858	G	4.88635E-16
	8:11392659:A:T	GTEx/v8	Cells_Cultured_fibroblasts	ENSG00000154319	FAM167A	8	11392659	A	2.75464E-10
	8:11392659:A:T	GTEx/v8	Cells_EBV-transformed_lymphocytes	ENSG00000154319	FAM167A	8	11392659	A	6.49307E-10
	8:11389224:C:G	GTEx/v8	Whole_Blood	ENSG00000154319	FAM167A	8	11389224	G	9.89402E-10
	8:11389783:A:C	GTEx/v8	Cells_Cultured_fibroblasts	ENSG00000154319	FAM167A	8	11389783	A	1.55573E-09
	8:11389783:A:C	GTEx/v8	Cells_EBV-transformed_lymphocytes	ENSG00000154319	FAM167A	8	11389783	A	3.44908E-09
	8:11388764:A:G	GTEx/v8	Whole_Blood	ENSG00000154319	FAM167A	8	11388764	A	6.99512E-09
	8:11389858:C:G	GTEx/v8	Cells_Cultured_fibroblasts	ENSG00000154319	FAM167A	8	11389858	G	2.13493E-08
	8:11389858:C:G	GTEx/v8	Cells_EBV-transformed_lymphocytes	ENSG00000154319	FAM167A	8	11389858	G	3.18535E-08
	8:11389783:A:C	GTEx/v8	Artery_Tibial	ENSG00000079459	FDFT1	8	11389783	A	1.42525E-07
	8:11392659:A:T	GTEx/v8	Artery_Tibial	ENSG00000079459	FDFT1	8	11392659	A	2.80128E-07
	1:226032677:A:G	GTEx/v8	Brain_Cerebellar_Hemisphere	ENSG00000143768	LEFTY2	1	226032677	G	6.82364E-07
	1:226070546:A:G	GTEx/v8	Brain_Cerebellar_Hemisphere	ENSG00000143768	LEFTY2	1	226070546	A	6.82364E-07
	8:11392659:A:T	GTEx/v8	Lung	ENSG00000136573	BLK	8	11392659	A	7.931E-07
	1:226032677:A:G	GTEx/v8	Brain_Cerebellum	ENSG00000143768	LEFTY2	1	226032677	G	1.47316E-06
	1:226070546:A:G	GTEx/v8	Brain_Cerebellum	ENSG00000143768	LEFTY2	1	226070546	A	1.47316E-06
	8:11388764:A:G	GTEx/v8	Whole_Blood	ENSG00000136573	BLK	8	11388764	A	2.03518E-06
	8:11392659:A:T	GTEx/v8	Cells_EBV-transformed_lymphocytes	ENSG00000136573	BLK	8	11392659	A	2.27931E-06
	8:11389858:C:G	GTEx/v8	Artery_Tibial	ENSG00000079459	FDFT1	8	11389858	G	2.70627E-06
	8:11389783:A:C	GTEx/v8	Lung	ENSG00000136573	BLK	8	11389783	A	3.38392E-06
	8:11389783:A:C	GTEx/v8	Cells_EBV-transformed_lymphocytes	ENSG00000136573	BLK	8	11389783	A	4.6159E-06
	8:11392659:A:T	GTEx/v8	Heart_Left_Ventricle	ENSG00000255394	C8orf49	8	11392659	A	5.38174E-06
	8:11389858:C:G	GTEx/v8	Lung	ENSG00000136573	BLK	8	11389858	G	6.01359E-06

**Posterior**	11:113195595:A:G	GTEx/v8	Pituitary	ENSG00000149292	TTC12	11	113195595	A	1.55519E-25
	11:113195595:A:G	GTEx/v8	Heart_Left_Ventricle	ENSG00000149292	TTC12	11	113195595	A	1.00232E-20
	11:113195595:A:G	GTEx/v8	Brain_Caudate_basal_ganglia	ENSG00000149292	TTC12	11	113195595	A	1.21968E-17
	17:7386279:C:T	GTEx/v8	Cells_Cultured_fibroblasts	ENSG00000161955	TNFSF13	17	7386279	T	6.18394E-16
	17:7403259:C:T	GTEx/v8	Cells_Cultured_fibroblasts	ENSG00000161955	TNFSF13	17	7403259	C	7.10261E-16
	17:7391920:C:T	GTEx/v8	Cells_Cultured_fibroblasts	ENSG00000161955	TNFSF13	17	7391920	C	8.88347E-16
	17:7394281:C:T	GTEx/v8	Cells_Cultured_fibroblasts	ENSG00000161955	TNFSF13	17	7394281	T	8.88347E-16
	17:7395801:C:T	GTEx/v8	Cells_Cultured_fibroblasts	ENSG00000161955	TNFSF13	17	7395801	C	8.88347E-16
	17:7401690:C:T	GTEx/v8	Cells_Cultured_fibroblasts	ENSG00000161955	TNFSF13	17	7401690	T	8.88347E-16
	11:113195595:A:G	GTEx/v8	Heart_Atrial_Appendage	ENSG00000149292	TTC12	11	113195595	a	2.69102E-15
	11:113195595:A:G	GTEx/v8	Brain_Anterior_cingulate_cortex_BA24	ENSG00000149292	TTC12	11	113195595	a	8.83003E-15
	11:113195595:A:G	GTEx/v8	Brain_Hypothalamus	ENSG00000149292	TTC12	11	113195595	a	1.05025E-13
	11:113195595:A:G	GTEx/v8	Brain_Putamen_basal_ganglia	ENSG00000149292	TTC12	11	113195595	a	2.51182E-13
	17:7391920:C:T	GTEx/v8	Whole_Blood	ENSG00000170175	CHRNB1	17	7391920	C	1.05284E-11
	17:7394281:C:T	GTEx/v8	Whole_Blood	ENSG00000170175	CHRNB1	17	7394281	T	1.05284E-11
	17:7395801:C:T	GTEx/v8	Whole_Blood	ENSG00000170175	CHRNB1	17	7395801	C	1.05284E-11
	17:7401690:C:T	GTEx/v8	Whole_Blood	ENSG00000170175	CHRNB1	17	7401690	T	1.05284E-11
	17:7403259:C:T	GTEx/v8	Whole_Blood	ENSG00000170175	CHRNB1	17	7403259	C	1.16018E-11
	17:7386279:C:T	GTEx/v8	Whole_Blood	ENSG00000170175	CHRNB1	17	7386279	T	1.53407E-11
	11:113195595:A:G	GTEx/v8	Brain_Hippocampus	ENSG00000149292	TTC12	11	113195595	a	2.49971E-11
	11:113195595:A:G	GTEx/v8	Whole_Blood	ENSG00000149292	TTC12	11	113195595	a	5.43544E-11
	11:113195595:A:G	GTEx/v8	Brain_Nucleus_accumbens_basal_ganglia	ENSG00000149292	TTC12	11	113195595	a	6.57033E-11
	11:113195595:A:G	GTEx/v8	Brain_Cortex	ENSG00000149292	TTC12	11	113195595	a	6.76894E-11
	17:7386279:C:T	GTEx/v8	Heart_Left_Ventricle	ENSG00000170175	CHRNB1	17	7386279	T	1.2993E-10
	17:7391920:C:T	GTEx/v8	Heart_Left_Ventricle	ENSG00000170175	CHRNB1	17	7391920	C	1.35145E-10
	17:7394281:C:T	GTEx/v8	Heart_Left_Ventricle	ENSG00000170175	CHRNB1	17	7394281	T	1.35145E-10
	17:7395801:C:T	GTEx/v8	Heart_Left_Ventricle	ENSG00000170175	CHRNB1	17	7395801	C	1.35145E-10
	17:7401690:C:T	GTEx/v8	Heart_Left_Ventricle	ENSG00000170175	CHRNB1	17	7401690	T	1.35145E-10
	17:7403259:C:T	GTEx/v8	Heart_Left_Ventricle	ENSG00000170175	CHRNB1	17	7403259	C	1.35145E-10
	17:7386279:C:T	GTEx/v8	Cells_Cultured_fibroblasts	ENSG00000170175	CHRNB1	17	7386279	T	1.51381E-10
	17:7391920:C:T	GTEx/v8	Cells_Cultured_fibroblasts	ENSG00000170175	CHRNB1	17	7391920	C	2.05059E-10
	17:7394281:C:T	GTEx/v8	Cells_Cultured_fibroblasts	ENSG00000170175	CHRNB1	17	7394281	T	2.05059E-10
	17:7395801:C:T	GTEx/v8	Cells_Cultured_fibroblasts	ENSG00000170175	CHRNB1	17	7395801	C	2.05059E-10
	17:7401690:C:T	GTEx/v8	Cells_Cultured_fibroblasts	ENSG00000170175	CHRNB1	17	7401690	T	2.05059E-10
	17:7403259:C:T	GTEx/v8	Cells_Cultured_fibroblasts	ENSG00000170175	CHRNB1	17	7403259	C	3.50435E-10
	11:113195595:A:G	GTEx/v8	Breast_Mammary_Tissue	ENSG00000149292	TTC12	11	113195595	A	5.04056E-10
	11:113195595:A:G	GTEx/v8	Brain_Substantia_nigra	ENSG00000149292	TTC12	11	113195595	A	9.08906E-10
	19:21951413:A:T	GTEx/v8	Heart_Left_Ventricle	ENSG00000197020	ZNF100	19	21951413	A	2.02532E-09
	11:113195595:A:G	GTEx/v8	Brain_Spinal_cord_cervical_c-1	ENSG00000149292	TTC12	11	113195595	A	3.09538E-09
	11:113195595:A:G	GTEx/v8	Artery_Tibial	ENSG00000170209	ANKK1	11	113195595	A	4.13705E-09
	11:113195595:A:G	GTEx/v8	Brain_Amygdala	ENSG00000149292	TTC12	11	113195595	A	4.49215E-09
	11:113195595:A:G	GTEx/v8	Whole_Blood	ENSG00000170209	ANKK1	11	113195595	A	4.57178E-09
	19:21881795:A:T	GTEx/v8	Heart_Left_Ventricle	ENSG00000197020	ZNF100	19	21881795	T	5.79743E-09
	19:21946686:C:T	GTEx/v8	Heart_Left_Ventricle	ENSG00000197020	ZNF100	19	21946686	T	5.79743E-09
	11:112210052:C:T	GTEx/v8	Artery_Tibial	ENSG00000197580	BCO2	11	112210052	C	6.85951E-09
	11:112214091:C:T	GTEx/v8	Artery_Tibial	ENSG00000197580	BCO2	11	112214091	C	6.85951E-09
	11:112210999:A:G	GTEx/v8	Artery_Tibial	ENSG00000197580	BCO2	11	112210999	G	7.03575E-09
	11:112211026:A:C	GTEx/v8	Artery_Tibial	ENSG00000197580	BCO2	11	112211026	A	7.03575E-09
	11:112212041:A:G	GTEx/v8	Artery_Tibial	ENSG00000197580	BCO2	11	112212041	G	7.03575E-09
	11:112211380:C:G	GTEx/v8	Artery_Tibial	ENSG00000197580	BCO2	11	112211380	G	7.07863E-09
	11:113195595:A:G	GTEx/v8	Brain_Frontal_Cortex_BA9	ENSG00000149292	TTC12	11	113195595	a	1.84882E-08
	17:7386279:C:T	GTEx/v8	Brain_Caudate_basal_ganglia	ENSG00000170175	CHRNB1	17	7386279	T	3.44314E-08
	17:7391920:C:T	GTEx/v8	Brain_Caudate_basal_ganglia	ENSG00000170175	CHRNB1	17	7391920	C	3.44314E-08
	17:7394281:C:T	GTEx/v8	Brain_Caudate_basal_ganglia	ENSG00000170175	CHRNB1	17	7394281	T	3.44314E-08
	17:7395801:C:T	GTEx/v8	Brain_Caudate_basal_ganglia	ENSG00000170175	CHRNB1	17	7395801	C	3.44314E-08
	17:7401690:C:T	GTEx/v8	Brain_Caudate_basal_ganglia	ENSG00000170175	CHRNB1	17	7401690	T	3.44314E-08
	17:7403259:C:T	GTEx/v8	Brain_Caudate_basal_ganglia	ENSG00000170175	CHRNB1	17	7403259	C	3.44314E-08
	17:7386279:C:T	GTEx/v8	Artery_Tibial	ENSG00000170175	CHRNB1	17	7386279	T	5.88023E-08
	17:7391920:C:T	GTEx/v8	Cells_Cultured_fibroblasts	ENSG00000259224	SLC35G6	17	7391920	C	5.99855E-08
	17:7394281:C:T	GTEx/v8	Cells_Cultured_fibroblasts	ENSG00000259224	SLC35G6	17	7394281	T	5.99855E-08
	17:7395801:C:T	GTEx/v8	Cells_Cultured_fibroblasts	ENSG00000259224	SLC35G6	17	7395801	C	5.99855E-08
	17:7401690:C:T	GTEx/v8	Cells_Cultured_fibroblasts	ENSG00000259224	SLC35G6	17	7401690	T	5.99855E-08
	17:7386279:C:T	GTEx/v8	Cells_Cultured_fibroblasts	ENSG00000259224	SLC35G6	17	7386279	T	6.6252E-08
	17:7403259:C:T	GTEx/v8	Cells_Cultured_fibroblasts	ENSG00000259224	SLC35G6	17	7403259	C	7.46827E-08
	17:7386279:C:T	GTEx/v8	Artery_Aorta	ENSG00000170175	CHRNB1	17	7386279	T	7.58636E-08
	11:113195595:A:G	GTEx/v8	Brain_Cerebellar_Hemisphere	ENSG00000149292	TTC12	11	113195595	a	8.04008E-08
	17:7401690:C:T	GTEx/v8	Artery_Aorta	ENSG00000170175	CHRNB1	17	7401690	T	8.72209E-08
	17:7391920:C:T	GTEx/v8	Artery_Tibial	ENSG00000170175	CHRNB1	17	7391920	C	8.75186E-08
	17:7394281:C:T	GTEx/v8	Artery_Tibial	ENSG00000170175	CHRNB1	17	7394281	T	8.75186E-08
	17:7395801:C:T	GTEx/v8	Artery_Tibial	ENSG00000170175	CHRNB1	17	7395801	C	8.75186E-08
	17:7403259:C:T	GTEx/v8	Artery_Tibial	ENSG00000170175	CHRNB1	17	7403259	C	1.1097E-07
	17:7391920:C:T	GTEx/v8	Artery_Aorta	ENSG00000170175	CHRNB1	17	7391920	C	1.24047E-07
	17:7394281:C:T	GTEx/v8	Artery_Aorta	ENSG00000170175	CHRNB1	17	7394281	T	1.24047E-07
	17:7395801:C:T	GTEx/v8	Artery_Aorta	ENSG00000170175	CHRNB1	17	7395801	C	1.24047E-07
	17:7403259:C:T	GTEx/v8	Artery_Aorta	ENSG00000170175	CHRNB1	17	7403259	C	1.24047E-07
	17:7401690:C:T	GTEx/v8	Artery_Tibial	ENSG00000170175	CHRNB1	17	7401690	T	1.38837E-07
	11:112210052:C:T	GTEx/v8	Heart_Left_Ventricle	ENSG00000197580	BCO2	11	112210052	C	5.40333E-07
	11:112210999:A:G	GTEx/v8	Heart_Left_Ventricle	ENSG00000197580	BCO2	11	112210999	G	5.40333E-07
	11:112211026:A:C	GTEx/v8	Heart_Left_Ventricle	ENSG00000197580	BCO2	11	112211026	A	5.40333E-07
	11:112212041:A:G	GTEx/v8	Heart_Left_Ventricle	ENSG00000197580	BCO2	11	112212041	G	5.40333E-07
	11:112214091:C:T	GTEx/v8	Heart_Left_Ventricle	ENSG00000197580	BCO2	11	112214091	C	5.40333E-07
	11:112211380:C:G	GTEx/v8	Heart_Left_Ventricle	ENSG00000197580	BCO2	11	112211380	G	5.91741E-07
	11:113195595:A:G	GTEx/v8	Brain_Cerebellum	ENSG00000149292	TTC12	11	113195595	A	6.61692E-07
	19:21951413:A:T	GTEx/v8	Artery_Tibial	ENSG00000197020	ZNF100	19	21951413	A	6.7242E-07
	19:21881795:A:T	GTEx/v8	Artery_Tibial	ENSG00000197020	ZNF100	19	21881795	T	8.80411E-07
	19:21946686:C:T	GTEx/v8	Artery_Tibial	ENSG00000197020	ZNF100	19	21946686	T	8.80411E-07
	11:112210052:C:T	GTEx/v8	Artery_Coronary	ENSG00000197580	BCO2	11	112210052	C	1.24605E-06
	11:112210999:A:G	GTEx/v8	Artery_Coronary	ENSG00000197580	BCO2	11	112210999	G	1.24605E-06
	11:112211026:A:C	GTEx/v8	Artery_Coronary	ENSG00000197580	BCO2	11	112211026	A	1.24605E-06
	11:112212041:A:G	GTEx/v8	Artery_Coronary	ENSG00000197580	BCO2	11	112212041	G	1.24605E-06
	11:112214091:C:T	GTEx/v8	Artery_Coronary	ENSG00000197580	BCO2	11	112214091	C	1.24605E-06
	17:7386279:C:T	GTEx/v8	Brain_Amygdala	ENSG00000161958	FGF11	17	7386279	T	1.35706E-06
	17:7391920:C:T	GTEx/v8	Brain_Amygdala	ENSG00000161958	FGF11	17	7391920	C	1.35706E-06
	17:7394281:C:T	GTEx/v8	Brain_Amygdala	ENSG00000161958	FGF11	17	7394281	T	1.35706E-06
	17:7395801:C:T	GTEx/v8	Brain_Amygdala	ENSG00000161958	FGF11	17	7395801	C	1.35706E-06
	17:7401690:C:T	GTEx/v8	Brain_Amygdala	ENSG00000161958	FGF11	17	7401690	T	1.35706E-06
	17:7403259:C:T	GTEx/v8	Brain_Amygdala	ENSG00000161958	FGF11	17	7403259	C	1.35706E-06
	11:112211380:C:G	GTEx/v8	Artery_Coronary	ENSG00000197580	BCO2	11	112211380	G	1.44646E-06
	10:104844314:G:T	GTEx/v8	Artery_Aorta	ENSG00000148843	PDCD11	10	104844314	G	1.58209E-06
	10:104895493:A:T	GTEx/v8	Artery_Aorta	ENSG00000148843	PDCD11	10	104895493	A	1.58209E-06
	17:7386279:C:T	GTEx/v8	Pituitary	ENSG00000170175	CHRNB1	17	7386279	T	1.69998E-06
	17:7391920:C:T	GTEx/v8	Pituitary	ENSG00000170175	CHRNB1	17	7391920	C	1.69998E-06
	17:7394281:C:T	GTEx/v8	Pituitary	ENSG00000170175	CHRNB1	17	7394281	T	1.69998E-06
	17:7395801:C:T	GTEx/v8	Pituitary	ENSG00000170175	CHRNB1	17	7395801	C	1.69998E-06
	17:7401690:C:T	GTEx/v8	Pituitary	ENSG00000170175	CHRNB1	17	7401690	T	1.69998E-06
	17:7403259:C:T	GTEx/v8	Pituitary	ENSG00000170175	CHRNB1	17	7403259	C	1.69998E-06
	19:21951413:A:T	GTEx/v8	Breast_Mammary_Tissue	ENSG00000197020	ZNF100	19	21951413	A	2.11271E-06
	19:21881795:A:T	GTEx/v8	Breast_Mammary_Tissue	ENSG00000197020	ZNF100	19	21881795	T	3.03712E-06
	19:21946686:C:T	GTEx/v8	Breast_Mammary_Tissue	ENSG00000197020	ZNF100	19	21946686	T	3.31651E-06
	11:112211380:C:G	GTEx/v8	Heart_Atrial_Appendage	ENSG00000197580	BCO2	11	112211380	G	3.46005E-06
	17:7386279:C:T	GTEx/v8	Breast_Mammary_Tissue	ENSG00000170175	CHRNB1	17	7386279	T	3.88049E-06
	11:112210052:C:T	GTEx/v8	Heart_Atrial_Appendage	ENSG00000197580	BCO2	11	112210052	C	4.19155E-06
	11:112210999:A:G	GTEx/v8	Heart_Atrial_Appendage	ENSG00000197580	BCO2	11	112210999	G	4.19155E-06
	11:112212041:A:G	GTEx/v8	Heart_Atrial_Appendage	ENSG00000197580	BCO2	11	112212041	G	4.19155E-06
	11:112214091:C:T	GTEx/v8	Heart_Atrial_Appendage	ENSG00000197580	BCO2	11	112214091	C	4.19155E-06
	19:21881795:A:T	GTEx/v8	Heart_Atrial_Appendage	ENSG00000197020	ZNF100	19	21881795	T	4.38895E-06
	19:21946686:C:T	GTEx/v8	Heart_Atrial_Appendage	ENSG00000197020	ZNF100	19	21946686	T	4.38895E-06
	11:112211026:A:C	GTEx/v8	Heart_Atrial_Appendage	ENSG00000197580	BCO2	11	112211026	A	4.64556E-06
	17:7386279:C:T	GTEx/v8	Brain_Nucleus_accumbens_basal_ganglia	ENSG00000170175	CHRNB1	17	7386279	T	5.27327E-06
	17:7391920:C:T	GTEx/v8	Brain_Nucleus_accumbens_basal_ganglia	ENSG00000170175	CHRNB1	17	7391920	C	5.27327E-06
	17:7394281:C:T	GTEx/v8	Brain_Nucleus_accumbens_basal_ganglia	ENSG00000170175	CHRNB1	17	7394281	T	5.27327E-06
	17:7395801:C:T	GTEx/v8	Brain_Nucleus_accumbens_basal_ganglia	ENSG00000170175	CHRNB1	17	7395801	C	5.27327E-06
	17:7401690:C:T	GTEx/v8	Brain_Nucleus_accumbens_basal_ganglia	ENSG00000170175	CHRNB1	17	7401690	T	5.27327E-06
	17:7403259:C:T	GTEx/v8	Brain_Nucleus_accumbens_basal_ganglia	ENSG00000170175	CHRNB1	17	7403259	C	5.27327E-06
	17:7391920:C:T	GTEx/v8	Breast_Mammary_Tissue	ENSG00000170175	CHRNB1	17	7391920	C	5.76448E-06
	17:7394281:C:T	GTEx/v8	Breast_Mammary_Tissue	ENSG00000170175	CHRNB1	17	7394281	T	5.76448E-06
	17:7395801:C:T	GTEx/v8	Breast_Mammary_Tissue	ENSG00000170175	CHRNB1	17	7395801	C	5.76448E-06
	17:7401690:C:T	GTEx/v8	Breast_Mammary_Tissue	ENSG00000170175	CHRNB1	17	7401690	T	5.76448E-06
	17:7403259:C:T	GTEx/v8	Breast_Mammary_Tissue	ENSG00000170175	CHRNB1	17	7403259	C	7.47775E-06
	17:7386279:C:T	GTEx/v8	Brain_Frontal_Cortex_BA9	ENSG00000170175	CHRNB1	17	7386279	T	9.51075E-06
	17:7391920:C:T	GTEx/v8	Brain_Frontal_Cortex_BA9	ENSG00000170175	CHRNB1	17	7391920	C	9.51075E-06
	17:7394281:C:T	GTEx/v8	Brain_Frontal_Cortex_BA9	ENSG00000170175	CHRNB1	17	7394281	T	9.51075E-06
	17:7395801:C:T	GTEx/v8	Brain_Frontal_Cortex_BA9	ENSG00000170175	CHRNB1	17	7395801	C	9.51075E-06
	17:7401690:C:T	GTEx/v8	Brain_Frontal_Cortex_BA9	ENSG00000170175	CHRNB1	17	7401690	T	9.51075E-06
	17:7403259:C:T	GTEx/v8	Brain_Frontal_Cortex_BA9	ENSG00000170175	CHRNB1	17	7403259	C	9.51075E-06

**Table 8:** Mendelian Randomization Analysis

Outcomes	Method	Beta	se	P

Ischemic stroke				
	Simple median	0.025	0.021	0.237
	Weighted median	0.025	0.021	0.23
	Inverse variance weighted (IVW)	0.017	0.016	0.292
	MR Egger	0.044	0.041	0.288
	MR Egger intercept	−0.021	0.031	0.484
Small vessel stroke				
	Simple median	0.001	0.028	0.983
	Weighted median	0.001	0.028	0.988
	Inverse variance weighted (IVW)	0.002	0.022	0.918
	MR Egger	0.012	0.078	0.882
	MR Egger intercept	−0.006	0.048	0.901
Coronary Artery Disease				
	Simple median	0.01	0.042	0.811
	Weighted median	0.01	0.042	0.811
	Inverse variance weighted (IVW)	0.017	0.034	0.621
	MR Egger	−0.042	0.115	0.717
	MR Egger intercept	0.041	0.078	0.594
Atrial Fibrillation				
	Simple median	0.008	0.03	0.8
	Weighted median	0.007	0.03	0.815
	Inverse variance weighted (IVW)	0.006	0.024	0.816
	MR Egger	−0.008	0.098	0.938
	MR Egger intercept	0.008	0.058	0.89

**Table 9:** MAGMA gene-based association

Asymptomatic ILAS	GENE	CHR	START	STOP	NSNPS	NPARAM	N	ZSTAT	P	SYMBOL

**Global**	ENSG00000268241	2	175198601	175204151	11	4	3666	4.1055	2.02E-05	AC018470.1
	ENSG00000217236	2	175197674	175205220	14	5	3652	3.7645	8.35E-05	SP9
	ENSG00000103187	16	84597200	84653683	99	27	4446	3.6664	0.00012299	COTL1
	ENSG00000253633	8	103538968	103552896	24	6	4070	3.5239	0.0002126	KB-1980E6.3
	ENSG00000137218	6	41735914	41756280	17	6	4187	3.3842	0.00035688	FRS3
	ENSG00000143493	1	212111741	212210884	63	10	3849	3.3842	0.00035693	INTS7
	ENSG00000124593	6	41746087	41759879	13	4	4084	3.3739	0.00037062	PRICKLE4
	ENSG00000141568	17	80475589	80604538	134	20	3942	3.2854	0.00050919	FOXK2
	ENSG00000214736	6	41753400	41759636	7	3	4201	3.2451	0.00058711	TOMM6
	ENSG00000013293	3	170175372	170305863	124	22	3863	3.1897	0.00071211	SLC7A14

**Anterior**	ENSG00000268327	15	59437899	59442054	1	1	3762	3.9984	3.19E-05	C15ORF31
	ENSG00000124593	6	41746087	41759879	11	3	4134	3.9517	3.88E-05	PRICKLE4
	ENSG00000137218	6	41735914	41756280	15	5	4215	3.9482	3.94E-05	FRS3
	ENSG00000214736	6	41753400	41759636	7	3	4201	3.8997	4.82E-05	TOMM6
	ENSG00000131037	19	55581388	55601291	16	4	4473	3.6158	0.00014971	EPS8L1
	ENSG00000103187	16	84597200	84653683	107	29	4232	3.5452	0.00019614	COTL1
	ENSG00000257950	17	3564357	3601488	26	8	3799	3.4752	0.0002552	P2RX5-TAX1BP3
	ENSG00000164663	6	41755634	41865099	37	6	4353	3.4082	0.00032698	USP49
	ENSG00000083454	17	3573493	3601698	20	7	3819	3.2594	0.00055829	P2RX5
	ENSG00000154016	17	18921986	18952950	7	2	4411	3.1186	0.00090869	GRAP

**Posterior**	ENSG00000143365	1	151776547	151806348	37	11	3787	3.881	5.20E-05	RORC
	ENSG00000077080	7	100238720	100256084	5	2	3872	3.8653	5.55E-05	ACTL6B
	ENSG00000173567	2	26529041	26571685	13	4	3764	3.4825	0.00024833	GPR113
	ENSG00000158483	11	71496556	71514282	33	4	3791	3.4799	0.00025083	FAM86C1
	ENSG00000106327	7	100216039	100242402	11	2	3813	3.4485	0.00028186	TFR2
	ENSG00000136286	7	45000261	45020697	14	5	4197	3.2842	0.00051137	MYO1G
	ENSG00000179456	1	244212585	244222778	6	3	3662	3.2701	0.00053757	ZBTB18
	ENSG00000106330	7	100207725	100215007	6	1	3868	3.2356	0.00060695	MOSPD3
	ENSG00000138018	2	26529415	26620759	32	5	3772	3.2197	0.00064165	EPT1
	ENSG00000134760	18	28896052	28938992	41	4	3840	3.2163	0.00064927	DSG1

## References

[1] Gorelick PB, Wong KS, Bae HJ, Pandey DK. Large artery intracranial occlusive disease: A large worldwide burden but a relatively neglected frontier. Stroke. 2008;39:2396–2399 18535283 10.1161/STROKEAHA.107.505776

[2] Kasner SE, Chimowitz MI, Lynn MJ, Howlett-Smith H, Stern BJ, Hertzberg VS, Frankel MR, Levine SR, Chaturvedi S, Benesch CG, Predictors of ischemic stroke in the territory of a symptomatic intracranial arterial stenosis. Circulation. 2006;113:555–563 16432056 10.1161/CIRCULATIONAHA.105.578229

[3] Hilal S, Xu X, Ikram MK, Vrooman H, Venketasubramanian N, Chen C. Intracranial stenosis in cognitive impairment and dementia. Journal of cerebral blood flow and metabolism : official journal of the International Society of Cerebral Blood Flow and Metabolism. 2017;37:2262–2269 10.1177/0271678X16663752 PMC5464715 27488908

[4] Hongo H, Miyawaki S, Imai H, Shimizu M, Yagi S, Mitsui J, Ishiura H, Yoshimura J, Doi K, Qu W, Comprehensive investigation of rnf213 nonsynonymous variants associated with intracranial artery stenosis. Sci Rep. 2020;10:11942 32686731 10.1038/s41598-020-68888-1 PMC7371676

[5] Qiao Y, Suri FK, Zhang Y, Liu L, Gottesman R, Alonso A, Guallar E, Wasserman BA. Racial differences in prevalence and risk for intracranial atherosclerosis in a us community-based population. JAMA cardiology. 2017;2:1341–1348 29094154 10.1001/jamacardio.2017.4041 PMC5814999

[6] Bang OY. Intracranial atherosclerosis: Current understanding and perspectives. J Stroke. 2014;16:27–35 24741562 10.5853/jos.2014.16.1.27 PMC3961814

[7] Miyawaki S, Imai H, Shimizu M, Yagi S, Ono H, Mukasa A, Nakatomi H, Shimizu T, Saito N. Genetic variant rnf213 c.14576g>a in various phenotypes of intracranial major artery stenosis/occlusion. Stroke. 2013;44:2894–2897 23970789 10.1161/STROKEAHA.113.002477

[8] Okazaki S, Morimoto T, Kamatani Y, Kamimura T, Kobayashi H, Harada K, Tomita T, Higashiyama A, Takahashi JC, Nakagawara J, Moyamoya disease susceptibility variant rnf213 p.R4810k increases the risk of ischemic stroke attributable to large-artery atherosclerosis. Circulation. 2019;139:295–298 30615506 10.1161/CIRCULATIONAHA.118.038439

[9] Kobayashi H, Kabata R, Kinoshita H, Morimoto T, Ono K, Takeda M, Choi J, Okuda H, Liu W, Harada KH, Rare variants in rnf213, a susceptibility gene for moyamoya disease, are found in patients with pulmonary hypertension and aggravate hypoxia-induced pulmonary hypertension in mice. Pulmonary circulation. 2018;8:2045894018778155 29718794 10.1177/2045894018778155 PMC5991195

[10] Morimoto T, Mineharu Y, Ono K, Nakatochi M, Ichihara S, Kabata R, Takagi Y, Cao Y, Zhao L, Kobayashi H, Significant association of rnf213 p.R4810k, a moyamoya susceptibility variant, with coronary artery disease. PLoS One. 2017;12:e0175649 28414759 10.1371/journal.pone.0175649 PMC5393571

[11] Suzuki H, Kataoka M, Hiraide T, Aimi Y, Yamada Y, Katsumata Y, Chiba T, Kanekura K, Isobe S, Sato Y, Genomic comparison with supercentenarians identifies rnf213 as a risk gene for pulmonary arterial hypertension. Circulation. Genomic and precision medicine. 2018;11:e002317 30562119 10.1161/CIRCGEN.118.002317

[12] Liu M, Sariya S, Khasiyev F, Tosto G, Dueker ND, Cheung YK, Wright CB, Sacco RL, Rundek T, Elkind MSV, Genetic determinants of intracranial large artery stenosis in the northern manhattan study. J Neurol Sci. 2022;436:120218 35259553 10.1016/j.jns.2022.120218 PMC9018518

[13] Cui M, Zhou S, Li R, Yin Z, Yu M, Zhou H. Association of adipoq single nucleotide polymorphisms with the risk of intracranial atherosclerosis. Int J Neurosci. 2017;127:427–432 27224208 10.1080/00207454.2016.1190716

[14] Kalita J, Somarajan BI, Kumar B, Kumar S, Mittal B, Misra UK. Phosphodiesterase 4 d gene polymorphism in relation to intracranial and extracranial atherosclerosis in ischemic stroke. Dis Markers. 2011;31:191–197 22045424 10.3233/DMA-2011-0810 PMC3826922

[15] Munshi A, Sharma V, Kaul S, Rajeshwar K, Babu MS, Shafi G, Anila AN, Balakrishna N, Alladi S, Jyothy A. Association of the −344c/t aldosterone synthase (cyp11b2) gene variant with hypertension and stroke. J Neurol Sci. 2010;296:34–38 20598712 10.1016/j.jns.2010.06.013

[16] Hurford R, Wolters FJ, Li L, Lau KK, Kuker W, Rothwell PM. Prognosis of asymptomatic intracranial stenosis in patients with transient ischemic attack and minor stroke. JAMA Neurol. 2020;77:947–954 32453401 10.1001/jamaneurol.2020.1326 PMC7251503

[17] Gutierrez J, Khasiyev F, Liu M, DeRosa JT, Tom SE, Rundek T, Cheung K, Wright CB, Sacco RL, Elkind MSV. Determinants and outcomes of asymptomatic intracranial atherosclerotic stenosis. J Am Coll Cardiol. 2021;78:562–571 34353533 10.1016/j.jacc.2021.05.041 PMC8352282

[18] Knopman DS, Gottesman RF, Sharrett AR, Wruck LM, Windham BG, Coker L, Schneider AL, Hengrui S, Alonso A, Coresh J, Mild cognitive impairment and dementia prevalence: The atherosclerosis risk in communities neurocognitive study (aric-ncs). Alzheimer’s & dementia (Amsterdam, Netherlands). 2016;2:1–11 10.1016/j.dadm.2015.12.002 PMC4772876 26949733

[19] Gutierrez J, Kulick E, Park Moon Y, Dong C, Cheung K, Ahmet B, Stern Y, Alperin N, Rundek T, Sacco RL, Brain arterial diameters and cognitive performance: The northern manhattan study. J Int Neuropsychol Soc. 2018;24:335–346 29166955 10.1017/S1355617717001175 PMC5860942

[20] Manly JJ, Bell-McGinty S, Tang MX, Schupf N, Stern Y, Mayeux R. Implementing diagnostic criteria and estimating frequency of mild cognitive impairment in an urban community. Archives of neurology. 2005;62:1739–1746 16286549 10.1001/archneur.62.11.1739

[21] Wong MYZ, Tan CS, Venketasubramanian N, Chen C, Ikram MK, Cheng CY, Hilal S. Prevalence and risk factors for cognitive impairment and dementia in indians: A multiethnic perspective from a singaporean study. J Alzheimers Dis. 2019;71:341–351 31381520 10.3233/JAD-190610

[22] Hilal S, Tan CS, Xin X, Amin SM, Wong TY, Chen C, Venketasubramanian N, Ikram MK. Prevalence of cognitive impairment and dementia in malays - epidemiology of dementia in singapore study. Curr Alzheimer Res. 2017;14:620–627 26428410 10.2174/1567205012666151002123813

[23] Hilal S, Ikram MK, Saini M, Tan CS, Catindig JA, Dong YH, Lim LB, Ting EY, Koo EH, Cheung CY, Prevalence of cognitive impairment in chinese: Epidemiology of dementia in singapore study. J Neurol Neurosurg Psychiatry. 2013;84:686–692 23385846 10.1136/jnnp-2012-304080

[24] Del Brutto VJ, Zambrano M, Mera RM, Del Brutto OH. Population-based study of cerebral microbleeds in stroke-free older adults living in rural ecuador: The atahualpa project. Stroke. 2015;46:1984–1986 26022640 10.1161/STROKEAHA.115.009594

[25] von Elm E, Altman DG, Egger M, Pocock SJ, Gotzsche PC, Vandenbroucke JP, Initiative S. The strengthening the reporting of observational studies in epidemiology (strobe) statement: Guidelines for reporting observational studies. J Clin Epidemiol. 2008;61:344–349 18313558 10.1016/j.jclinepi.2007.11.008

[26] Liu M, Khasiyev F, Sariya S, Spagnolo-Allende A, Sanchez DL, Andrews H, Yang Q, Beiser A, Qiao Y, Thomas EA, Chromosome 10q24.32 variants associate with brain arterial diameters in diverse populations: A genome-wide association study. 2023:2023.2001.2031.23285251 10.1161/JAHA.123.030935 PMC10727334 38038215

[27] Chimowitz MI, Lynn MJ, Howlett-Smith H, Stern BJ, Hertzberg VS, Frankel MR, Levine SR, Chaturvedi S, Kasner SE, Benesch CGJNEJoM. Comparison of warfarin and aspirin for symptomatic intracranial arterial stenosis. 2005;352:1305–1316 10.1056/NEJMoa043033 15800226

[28] Liao KP, Sun J, Cai TA, Link N, Hong C, Huang J, Huffman JE, Gronsbell J, Zhang Y, Ho YL, High-throughput multimodal automated phenotyping (map) with application to phewas. J Am Med Inform Assoc. 2019;26:1255–1262 31613361 10.1093/jamia/ocz066 PMC6798574

[29] Willer CJ, Li Y, Abecasis GR. Metal: Fast and efficient meta-analysis of genomewide association scans. Bioinformatics. 2010;26:2190–2191 20616382 10.1093/bioinformatics/btq340 PMC2922887

[30] Watanabe K, Taskesen E, van Bochoven A, Posthuma D. Functional mapping and annotation of genetic associations with fuma. Nat Commun. 2017;8:1826 29184056 10.1038/s41467-017-01261-5 PMC5705698

[31] de Leeuw CA, Mooij JM, Heskes T, Posthuma D. Magma: Generalized gene-set analysis of gwas data. PLoS Comput Biol. 2015;11:e1004219 25885710 10.1371/journal.pcbi.1004219 PMC4401657

[32] Liberzon A, Birger C, Thorvaldsdottir H, Ghandi M, Mesirov JP, Tamayo P. The molecular signatures database (msigdb) hallmark gene set collection. Cell Syst. 2015;1:417–425 26771021 10.1016/j.cels.2015.12.004 PMC4707969

[33] Consortium GT. Human genomics. The genotype-tissue expression (gtex) pilot analysis: Multitissue gene regulation in humans. Science. 2015;348:648–660 25954001 10.1126/science.1262110 PMC4547484

[34] Lek M, Karczewski KJ, Minikel EV, Samocha KE, Banks E, Fennell T, O’Donnell-Luria AH, Ware JS, Hill AJ, Cummings BB, Analysis of protein-coding genetic variation in 60,706 humans. Nature. 2016;536:285–291 27535533 10.1038/nature19057 PMC5018207

[35] Petrovski S, Gussow AB, Wang Q, Halvorsen M, Han Y, Weir WH, Allen AS, Goldstein DB. The intolerance of regulatory sequence to genetic variation predicts gene dosage sensitivity. PLoS genetics. 2015;11:e1005492 26332131 10.1371/journal.pgen.1005492 PMC4557908

[36] Schmitt AD, Hu M, Jung I, Xu Z, Qiu Y, Tan CL, Li Y, Lin S, Lin Y, Barr CL, A compendium of chromatin contact maps reveals spatially active regions in the human genome. Cell reports. 2016;17:2042–2059 27851967 10.1016/j.celrep.2016.10.061 PMC5478386

[37] Malik R, Chauhan G, Traylor M, Sargurupremraj M, Okada Y, Mishra A, Rutten-Jacobs L, Giese AK, van der Laan SW, Gretarsdottir S, Multiancestry genome-wide association study of 520,000 subjects identifies 32 loci associated with stroke and stroke subtypes. Nature genetics. 2018;50:524–537 29531354 10.1038/s41588-018-0058-3 PMC5968830

[38] Miyazawa K, Ito K, Ito M, Zou Z, Kubota M, Nomura S, Matsunaga H, Koyama S, Ieki H, Akiyama M, Cross-ancestry genome-wide analysis of atrial fibrillation unveils disease biology and enables cardioembolic risk prediction. Nature genetics. 2023;55:187–197 36653681 10.1038/s41588-022-01284-9 PMC9925380

[39] Institute WS. Coronary artery disease: Ftp://ftp.Sanger.Ac.Uk/pub/cardiogramplusc4d/cardiogram_gwas_results.Zip. 2015

[40] Hemani G, Zheng J, Elsworth B, Wade KH, Haberland V, Baird D, Laurin C, Burgess S, Bowden J, Langdon R, The mr-base platform supports systematic causal inference across the human phenome. eLife. 2018;7 10.7554/eLife.34408 PMC5976434 29846171

[41] Soh J, Iqbal J, Queiroz J, Fernandez-Hernando C, Hussain MM. Microrna-30c reduces hyperlipidemia and atherosclerosis in mice by decreasing lipid synthesis and lipoprotein secretion. Nat Med. 2013;19:892–900 23749231 10.1038/nm.3200 PMC4121125

[42] Wei Z, Zhao J, Niebler J, Hao JJ, Merrick BA, Xia M. Quantitative proteomic profiling of mitochondrial toxicants in a human cardiomyocyte cell line. Front Genet. 2020;11:719 32733541 10.3389/fgene.2020.00719 PMC7358379

[43] Traurig MT, Orczewska JI, Ortiz DJ, Bian L, Marinelarena AM, Kobes S, Malhotra A, Hanson RL, Mason CC, Knowler WC, Evidence for a role of lpgat1 in influencing bmi and percent body fat in native americans. Obesity (Silver Spring). 2013;21:193–202 23505186 10.1002/oby.20243 PMC3666094

[44] Hou J, Aerts J, den Hamer B, van Ijcken W, den Bakker M, Riegman P, van der Leest C, van der Spek P, Foekens JA, Hoogsteden HC, Gene expression-based classification of non-small cell lung carcinomas and survival prediction. PLoS One. 2010;5:e10312 20421987 10.1371/journal.pone.0010312 PMC2858668

[45] Geng Y, Deng L, Su D, Xiao J, Ge D, Bao Y, Jing H. Identification of crucial micrornas and genes in hypoxia-induced human lung adenocarcinoma cells. Onco Targets Ther. 2016;9:4605–4616 27524914 10.2147/OTT.S103430 PMC4966693

[46] Gong H, Ma C, Li X, Zhang X, Zhang L, Chen P, Wang W, Hu Y, Huang T, Wu N, Upregulation of lpgat1 enhances lung adenocarcinoma proliferation. Front Biosci (Landmark Ed). 2023;28:89 37258473 10.31083/j.fbl2805089

[47] Wei R, Ngo B, Wu G, Lee WH. Phosphorylation of the ndc80 complex protein, hec1, by nek2 kinase modulates chromosome alignment and signaling of the spindle assembly checkpoint. Mol Biol Cell. 2011;22:3584–3594 21832156 10.1091/mbc.E11-01-0012 PMC3183014

[48] Feng X, Jiang Y, Cui Y, Xu Y, Zhang Q, Xia Q, Chen Y. Nek2 is associated with poor prognosis of clear cell renal cell carcinoma and promotes tumor cell growth and metastasis. Gene. 2023;851:147040 36370999 10.1016/j.gene.2022.147040

[49] Zhang X, Huang X, Xu J, Li E, Lao M, Tang T, Zhang G, Guo C, Zhang X, Chen W, Nek2 inhibition triggers anti-pancreatic cancer immunity by targeting pd-l1. Nat Commun. 2021;12:4536 34315872 10.1038/s41467-021-24769-3 PMC8316469

[50] Fang Y, Zhang X. Targeting nek2 as a promising therapeutic approach for cancer treatment. Cell cycle (Georgetown, Tex.). 2016;15:895–907 27019372 10.1080/15384101.2016.1152430 PMC4889274

[51] Li X, Yao Y, Qian J, Jin G, Zeng G, Zhao H. Overexpression and diagnostic significance of ints7 in lung adenocarcinoma and its effects on tumor microenvironment. Int Immunopharmacol. 2021;101:108346 34781123 10.1016/j.intimp.2021.108346

[52] Federico A, Rienzo M, Abbondanza C, Costa V, Ciccodicola A, Casamassimi A. Pan-cancer mutational and transcriptional analysis of the integrator complex. Int J Mol Sci. 2017;18 28468258 10.3390/ijms18050936 PMC5454849

[53] Greenwood TA, Akiskal HS, Akiskal KK, Bipolar Genome S, Kelsoe JR. Genome-wide association study of temperament in bipolar disorder reveals significant associations with three novel loci. Biol Psychiatry. 2012;72:303–310 22365631 10.1016/j.biopsych.2012.01.018 PMC3925336

[54] Li Z, Wang R, Qiu C, Cao C, Zhang J, Ge J, Shi Y. Role of dtl in hepatocellular carcinoma and its impact on the tumor microenvironment. Front Immunol. 2022;13:834606 35392073 10.3389/fimmu.2022.834606 PMC8980229

[55] Jin J, Arias EE, Chen J, Harper JW, Walter JC. A family of diverse cul4-ddb1-interacting proteins includes cdt2, which is required for s phase destruction of the replication factor cdt1. Molecular cell. 2006;23:709–721 16949367 10.1016/j.molcel.2006.08.010

[56] Terai K, Abbas T, Jazaeri AA, Dutta A. Crl4(cdt2) e3 ubiquitin ligase monoubiquitinates pcna to promote translesion DNA synthesis. Molecular cell. 2010;37:143–149 20129063 10.1016/j.molcel.2009.12.018 PMC2818832

[57] Evans DL, Zhang H, Ham H, Pei H, Lee S, Kim J, Billadeau DD, Lou Z. Mmset is dynamically regulated during cell-cycle progression and promotes normal DNA replication. Cell cycle (Georgetown, Tex.). 2016;15:95–105 26771714 10.1080/15384101.2015.1121323 PMC4825781

[58] Vanderdys V, Allak A, Guessous F, Benamar M, Read PW, Jameson MJ, Abbas T. The neddylation inhibitor pevonedistat (mln4924) suppresses and radiosensitizes head and neck squamous carcinoma cells and tumors. Molecular cancer therapeutics. 2018;17:368–380 28838998 10.1158/1535-7163.MCT-17-0083 PMC5805645

[59] Kiran S, Dar A, Singh SK, Lee KY, Dutta A. The deubiquitinase usp46 is essential for proliferation and tumor growth of hpv-transformed cancers. Molecular cell. 2018;72:823–835.e825 30415951 10.1016/j.molcel.2018.09.019 PMC6294304

[60] Pissas KP, Gründer S, Tian Y. Functional expression of the proton sensors asic1a, tmem206, and ogr1 together with bk(ca) channels is associated with cell volume changes and cell death under strongly acidic conditions in daoy medulloblastoma cells. Pflugers Arch. 2024;476:923–937 38627262 10.1007/s00424-024-02964-7 PMC11139714

[61] Ullrich F, Blin S, Lazarow K, Daubitz T, von Kries JP, Jentsch TJ. Identification of tmem206 proteins as pore of paorac/asor acid-sensitive chloride channels. eLife. 2019;8 10.7554/eLife.49187 PMC6663466 31318332

[62] Yang J, Chen J, Del Carmen Vitery M, Osei-Owusu J, Chu J, Yu H, Sun S, Qiu Z. Pac, an evolutionarily conserved membrane protein, is a proton-activated chloride channel. Science. 2019;364:395–399 31023925 10.1126/science.aav9739 PMC7305803

[63] Wang HY, Shimizu T, Numata T, Okada Y. Role of acid-sensitive outwardly rectifying anion channels in acidosis-induced cell death in human epithelial cells. Pflugers Arch. 2007;454:223–233 17186306 10.1007/s00424-006-0193-z

[64] Osei-Owusu J, Yang J, Leung KH, Ruan Z, Lu W, Krishnan Y, Qiu Z. Proton-activated chloride channel pac regulates endosomal acidification and transferrin receptor-mediated endocytosis. Cell reports. 2021;34:108683 33503418 10.1016/j.celrep.2020.108683 PMC7869721

[65] Zeziulia M, Blin S, Schmitt FW, Lehmann M, Jentsch TJ. Proton-gated anion transport governs macropinosome shrinkage. Nat Cell Biol. 2022;24:885–895 35590106 10.1038/s41556-022-00912-0 PMC9203271

[66] Song S, Zhang Y, Ding T, Ji N, Zhao H. The dual role of macropinocytosis in cancers: Promoting growth and inducing methuosis to participate in anticancer therapies as targets. Frontiers in oncology. 2020;10:570108 33542897 10.3389/fonc.2020.570108 PMC7851083

[67] Kotnis S, Bingham B, Vasilyev DV, Miller SW, Bai Y, Yeola S, Chanda PK, Bowlby MR, Kaftan EJ, Samad TA, Genetic and functional analysis of human p2x5 reveals a distinct pattern of exon 10 polymorphism with predominant expression of the nonfunctional receptor isoform. Molecular pharmacology. 2010;77:953–960 20223879 10.1124/mol.110.063636

[68] Zhang Y, Babczyk P, Pansky A, Kassack MU, Tobiasch E. P2 receptors influence hmscs differentiation towards endothelial cell and smooth muscle cell lineages. Int J Mol Sci. 2020;21 32867347 10.3390/ijms21176210 PMC7503934

[69] Schwiebert LM, Rice WC, Kudlow BA, Taylor AL, Schwiebert EM. Extracellular atp signaling and p2x nucleotide receptors in monolayers of primary human vascular endothelial cells. Am J Physiol Cell Physiol. 2002;282:C289–301 11788340 10.1152/ajpcell.01387.2000

[70] Lalo U, Pankratov Y, Wichert SP, Rossner MJ, North RA, Kirchhoff F, Verkhratsky A. P2x1 and p2x5 subunits form the functional p2x receptor in mouse cortical astrocytes. J Neurosci. 2008;28:5473–5480 18495881 10.1523/JNEUROSCI.1149-08.2008 PMC3844808

[71] Abramowski P, Ogrodowczyk C, Martin R, Pongs O. A truncation variant of the cation channel p2rx5 is upregulated during t cell activation. PLoS One. 2014;9:e104692 25181038 10.1371/journal.pone.0104692 PMC4152149

[72] King BF. Rehabilitation of the p2x5 receptor: A re-evaluation of structure and function. Purinergic signalling. 2023;19:421–439 36279087 10.1007/s11302-022-09903-0 PMC10247652

[73] Yap CX, Vo DD, Heffel MG, Bhattacharya A, Wen C, Yang Y, Kemper KE, Zeng J, Zheng Z, Zhu Z, Brain cell-type shifts in alzheimer’s disease, autism, and schizophrenia interrogated using methylomics and genetics. Science advances. 2024;10:eadn7655 38781333 10.1126/sciadv.adn7655 PMC11114225

[74] Hooper JD, Clements JA, Quigley JP, Antalis TM. Type ii transmembrane serine proteases. Insights into an emerging class of cell surface proteolytic enzymes. J Biol Chem. 2001;276:857–860 11060317 10.1074/jbc.R000020200

[75] Szabo R, Wu Q, Dickson RB, Netzel-Arnett S, Antalis TM, Bugge TH. Type ii transmembrane serine proteases. Thrombosis and haemostasis. 2003;90:185–193 12888865 10.1160/TH03-02-0071

[76] Antalis TM, Buzza MS, Hodge KM, Hooper JD, Netzel-Arnett S. The cutting edge: Membrane-anchored serine protease activities in the pericellular microenvironment. Biochem J. 2010;428:325–346 20507279 10.1042/BJ20100046 PMC3680374

[77] Luostari K, Hartikainen JM, Tengstrom M, Palvimo JJ, Kataja V, Mannermaa A, Kosma VM. Type ii transmembrane serine protease gene variants associate with breast cancer. PLoS One. 2014;9:e102519 25029565 10.1371/journal.pone.0102519 PMC4100901

[78] Yamada Y, Sakuma J, Takeuchi I, Yasukochi Y, Kato K, Oguri M, Fujimaki T, Horibe H, Muramatsu M, Sawabe M, Identification of six polymorphisms as novel susceptibility loci for ischemic or hemorrhagic stroke by exome-wide association studies. International journal of molecular medicine. 2017;39:1477–1491 28487959 10.3892/ijmm.2017.2972 PMC5428971

