# Supplementary material for: Multi-population Genome-Wide Association Study Identifies Multiple Novel Loci associated with Asymptomatic Intracranial Large Artery Stenosis
Source: medRxiv. 2025 May 7:2025.05.06.25327093. Preprint. [Version 1] doi: 10.1101/2025.05.06.25327093 (PMC12083599; doi:10.1101/2025.05.06.25327093)
Supplement: Supplement 1 [file media-1.zip › Supp_Figures_04092025.docx]

Figure 1 Manhattan Plot of GWAS summary statistics for Global ILAS. (A) Asian. (B) African American. (C) White. (D) Hispanic.

| A | 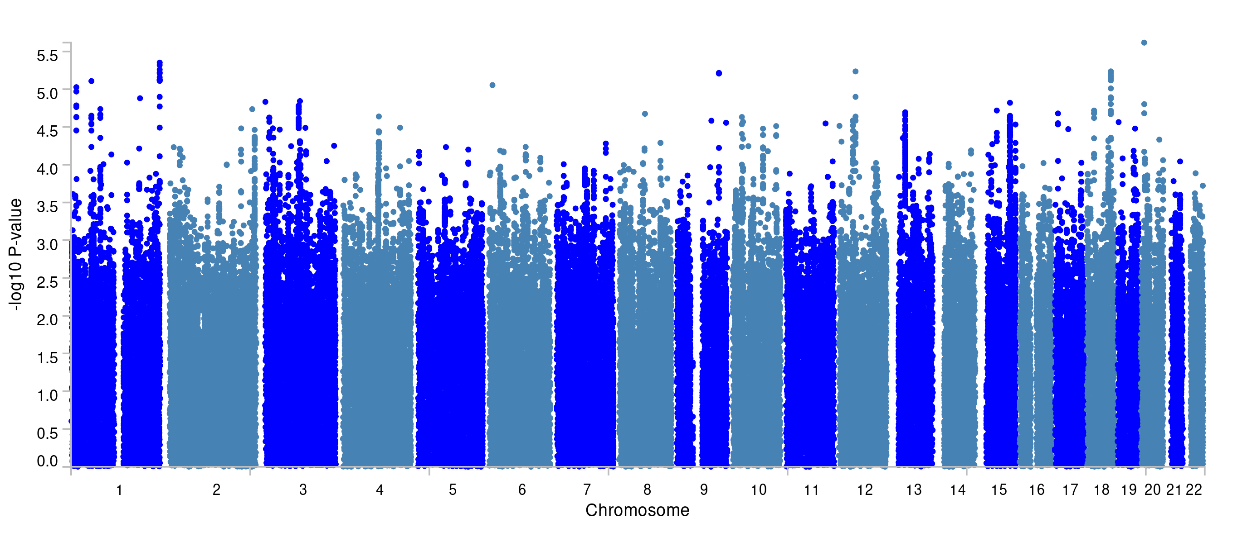 |
| --- | --- |
| B | 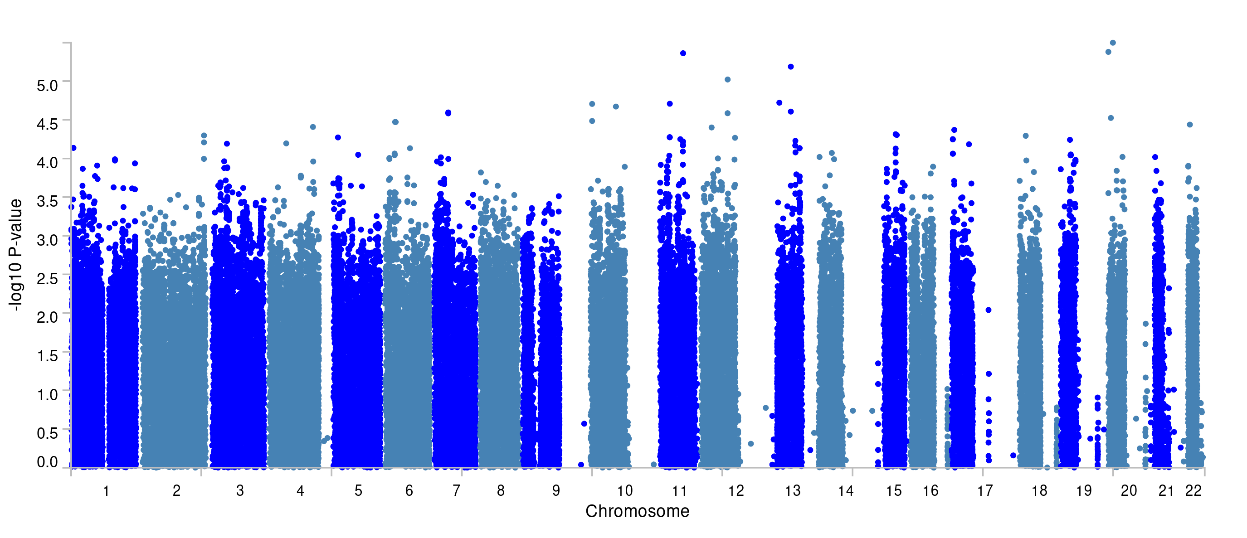 |
| C | 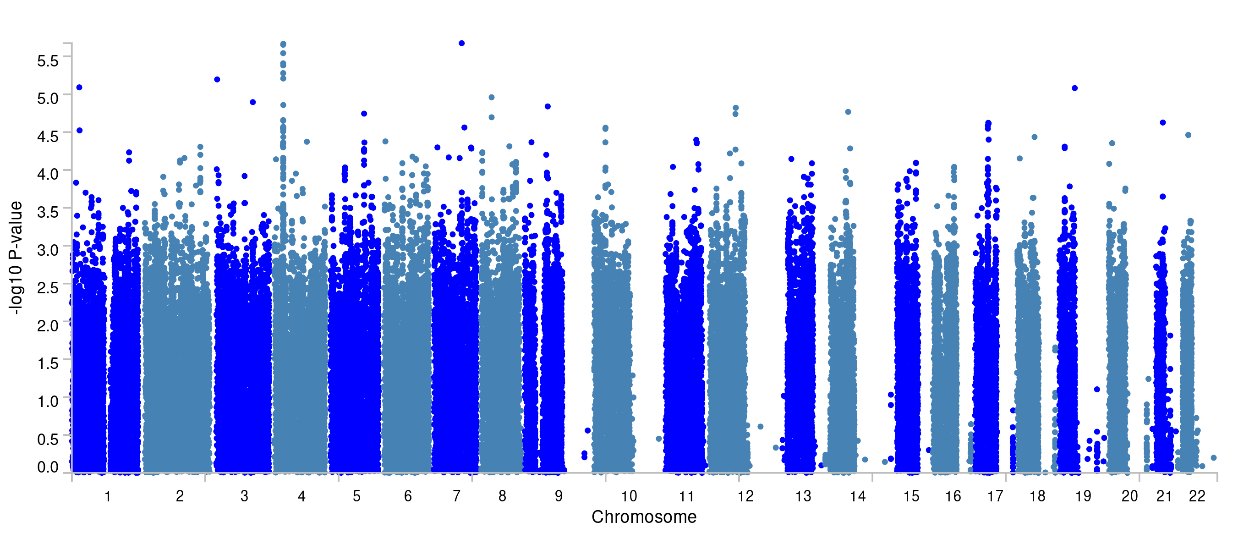 |
| D | 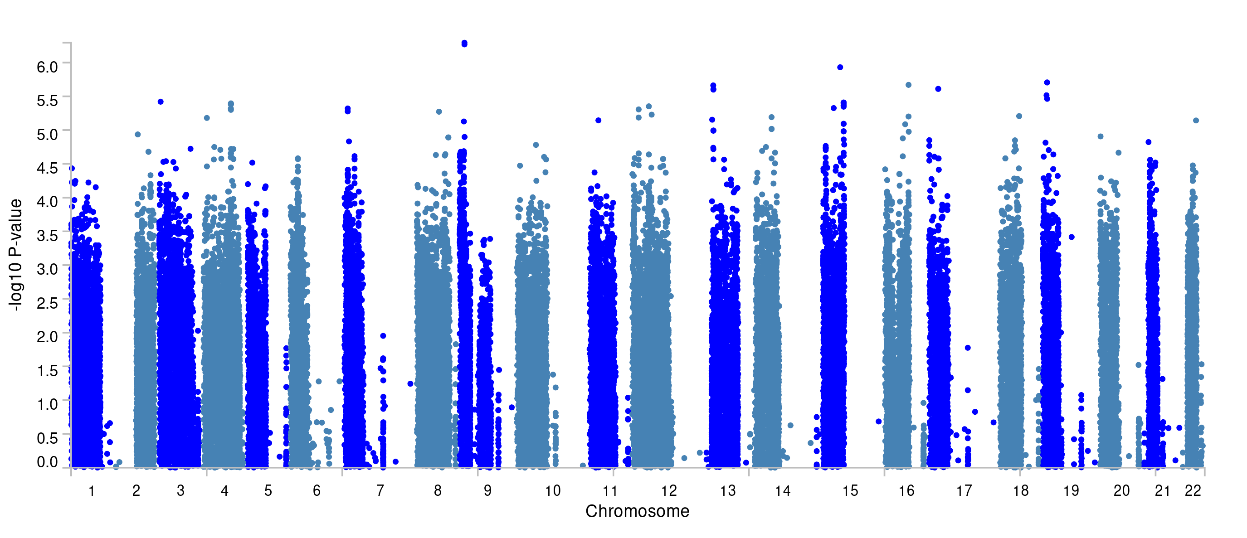 |

Figure 2 Manhattan Plot of GWAS summary statistics for Anterior ILAS. (A) Asian. (B) African American. (C) White. (D) Hispanic.

| A | 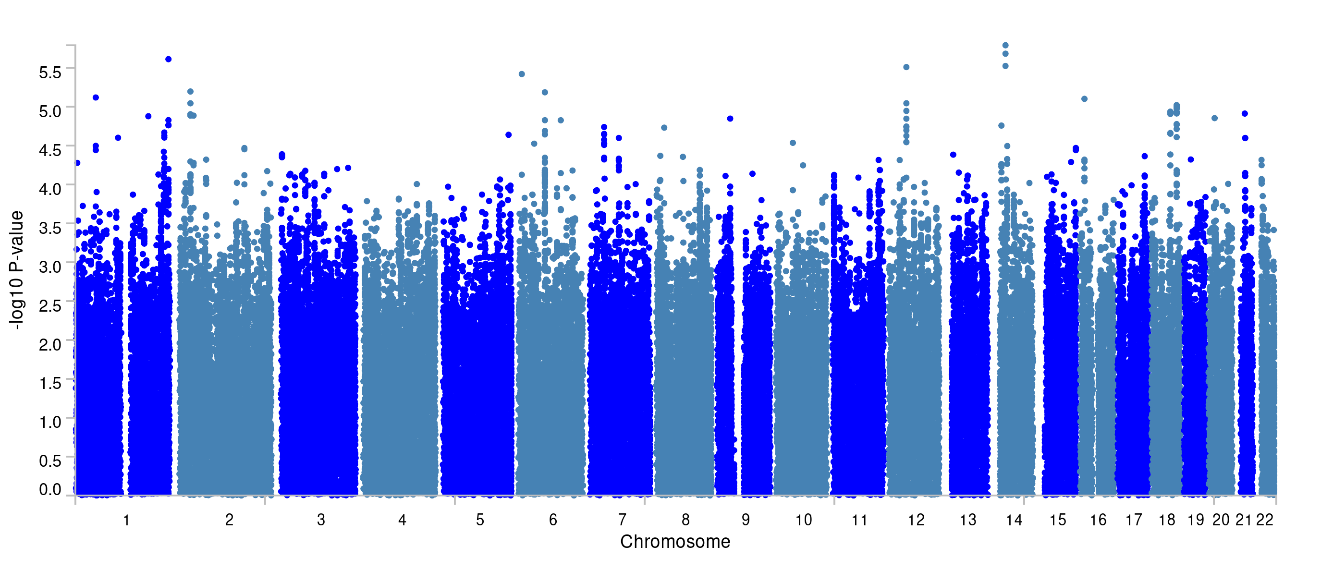 |
| --- | --- |
| B | 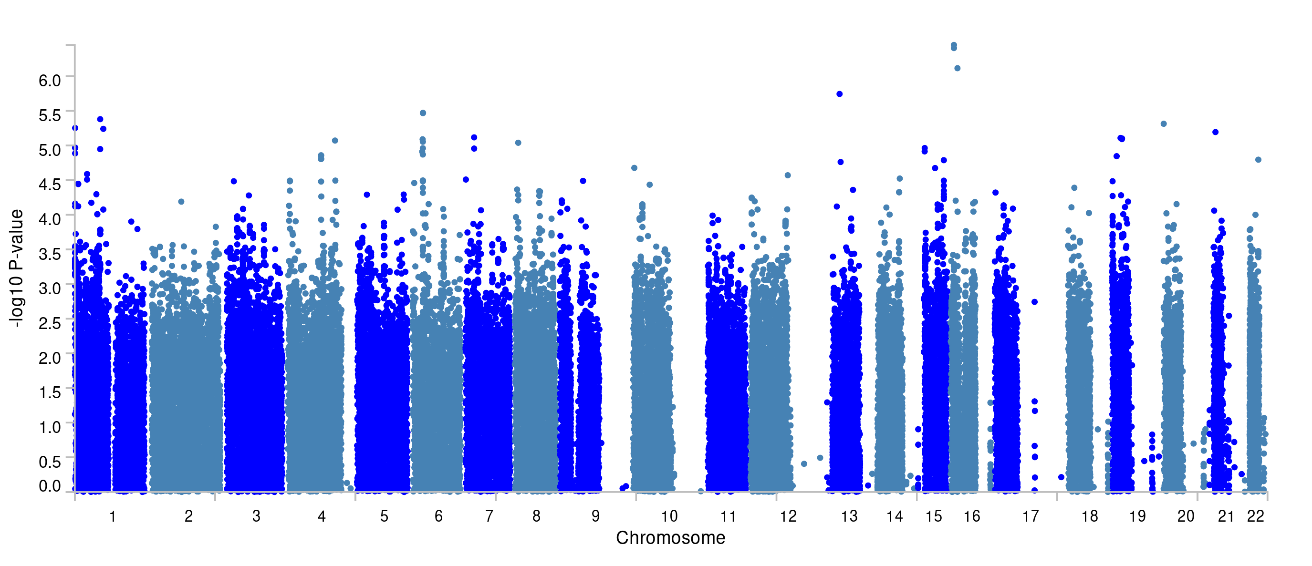 |
| C | 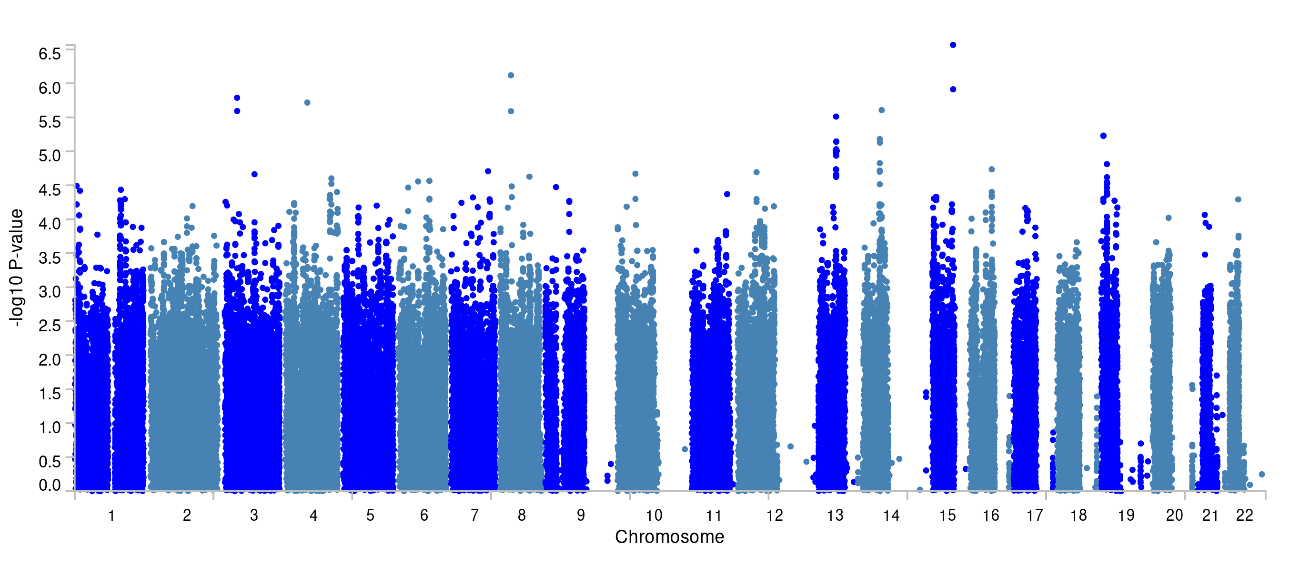 |
| D | 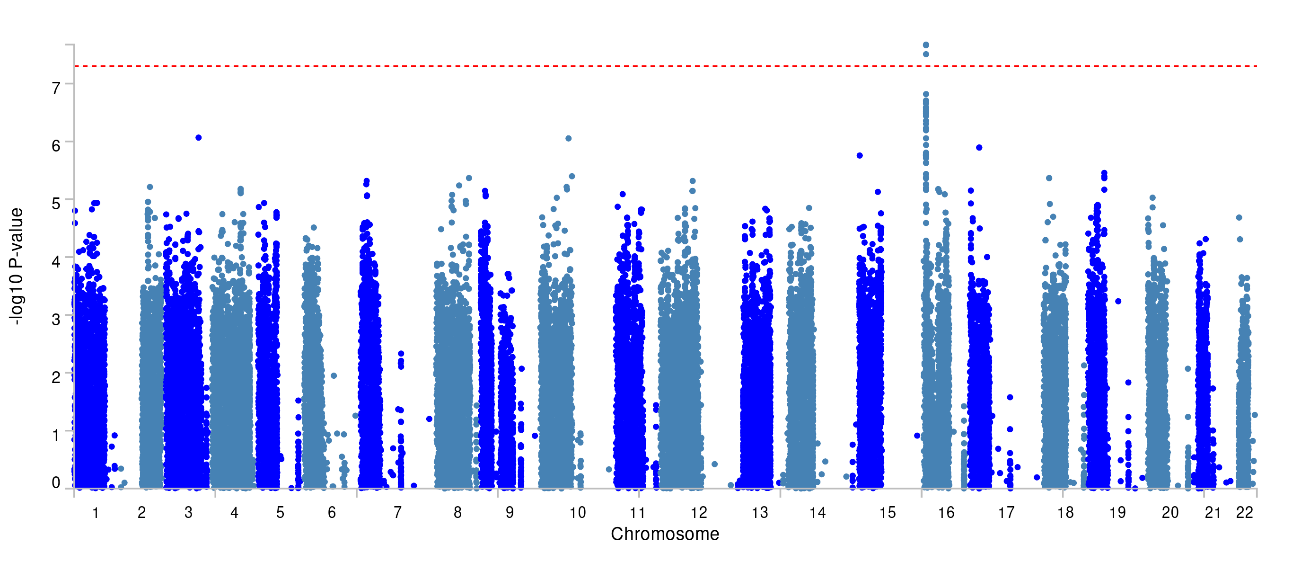 |

Figure 3 Manhattan Plot of GWAS summary statistics for Posterior ILAS. (A) Asian. (B) African American. (C) White. (D) Hispanic.

| A | 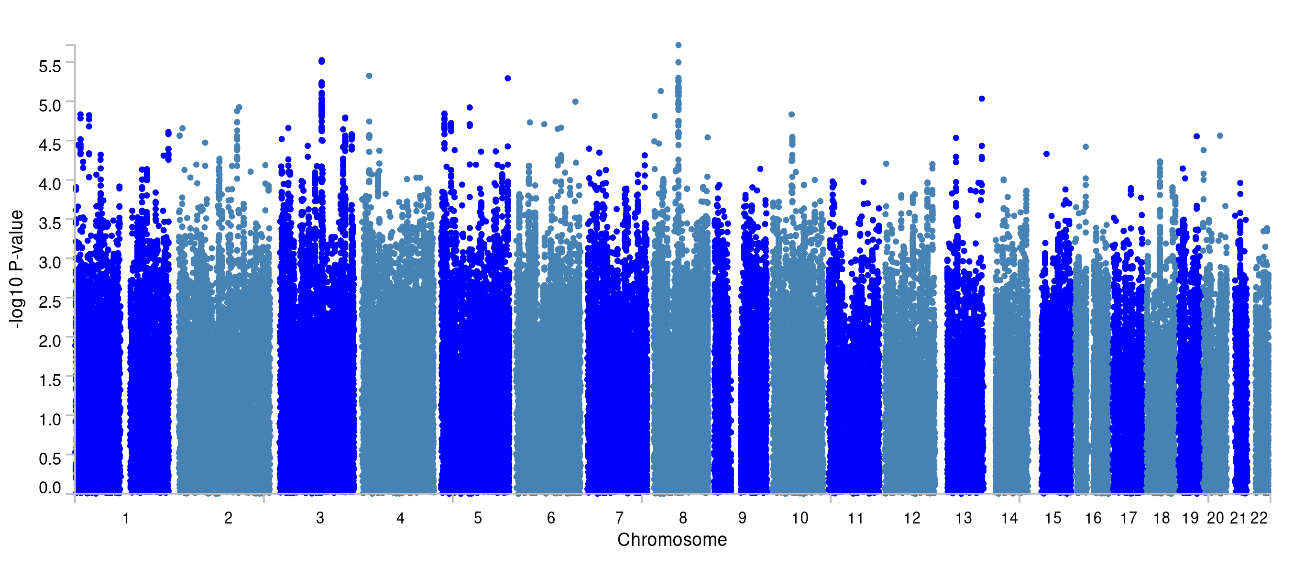 |
| --- | --- |
| B | 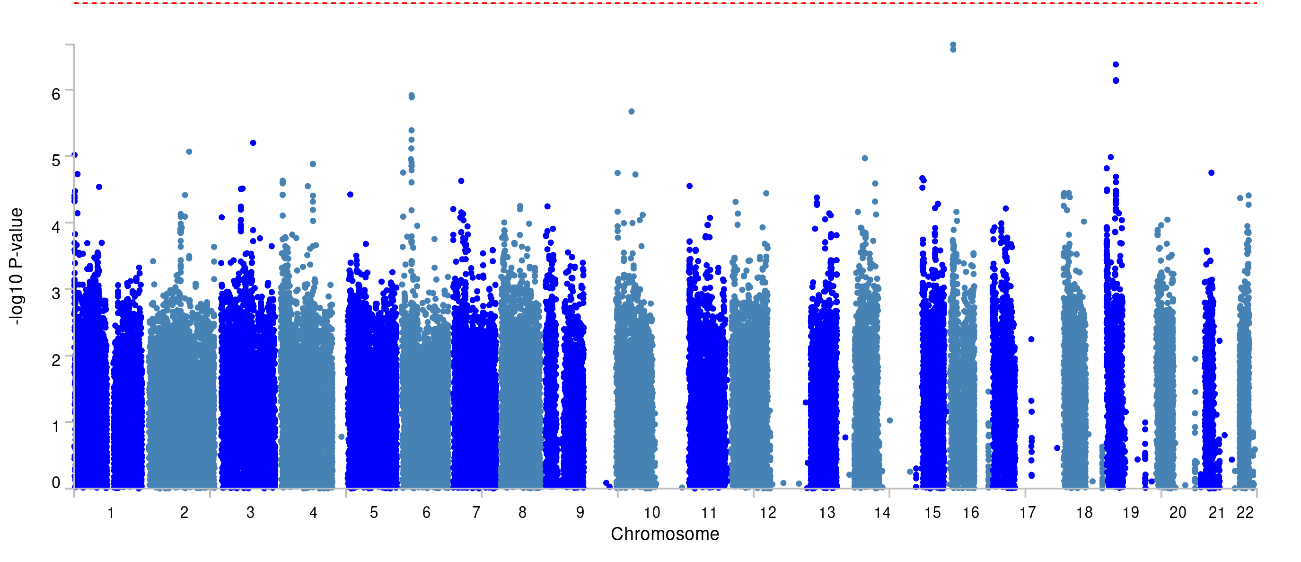 |
| C | 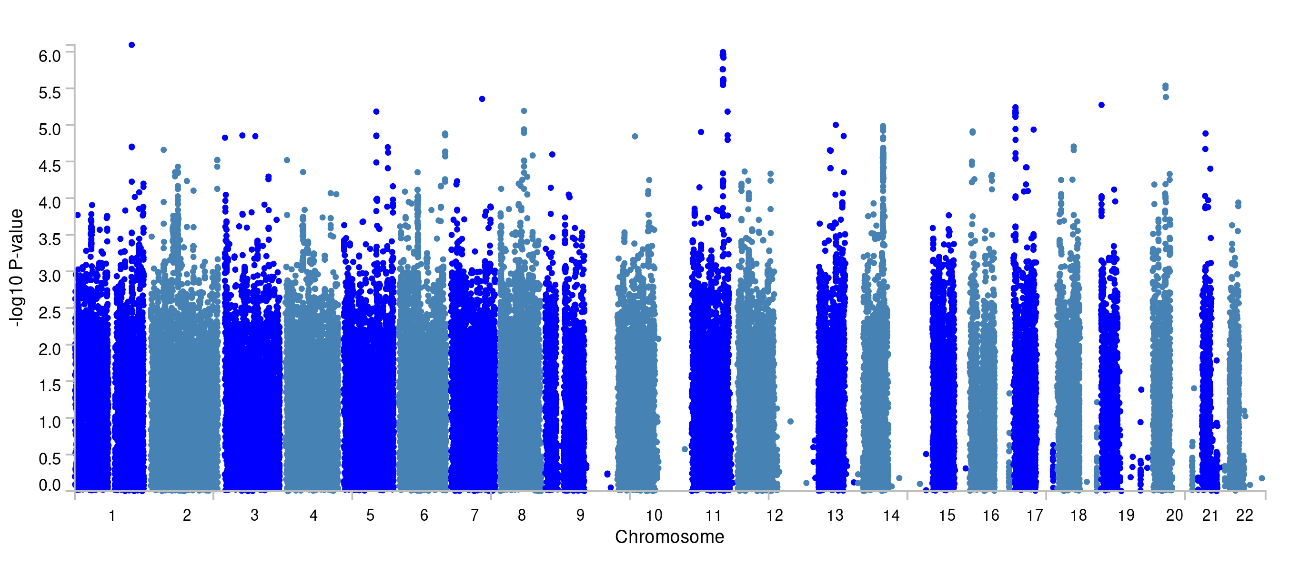 |
| D | 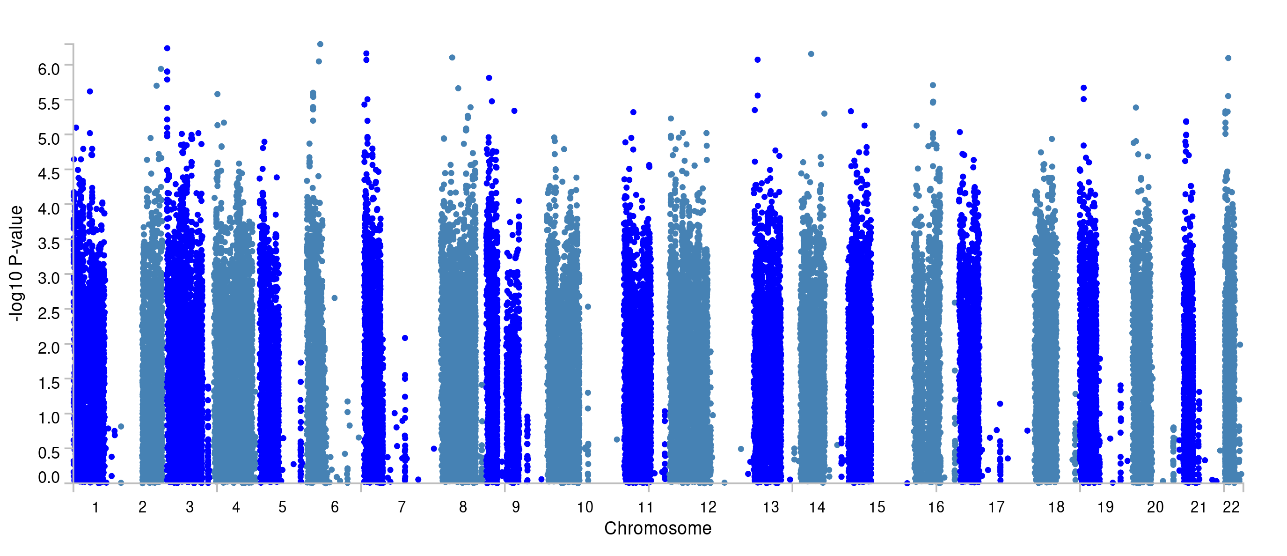 |

Figure 4 QQ plot of GWAS summary statistics for Global ILAS. (A) Asian. (B) African American. (C) White. (D) Hispanic.

| 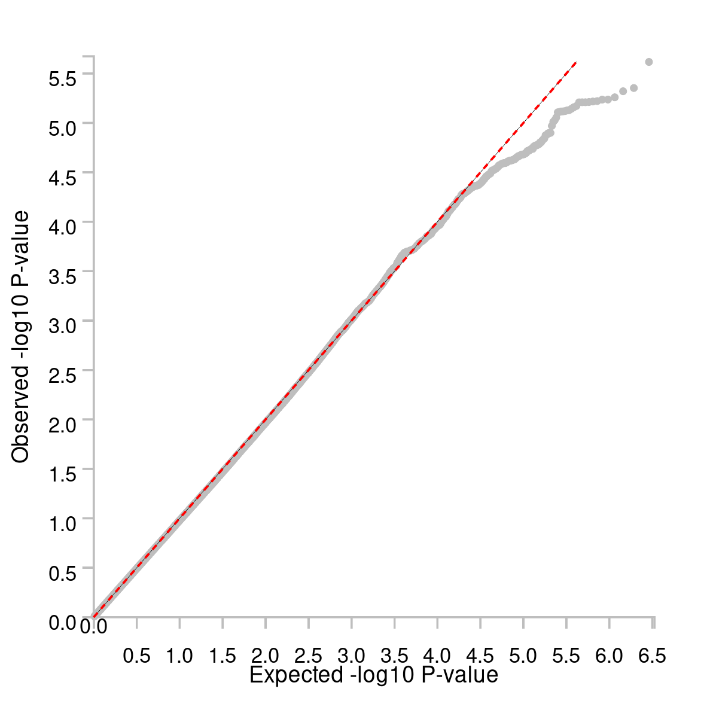 | 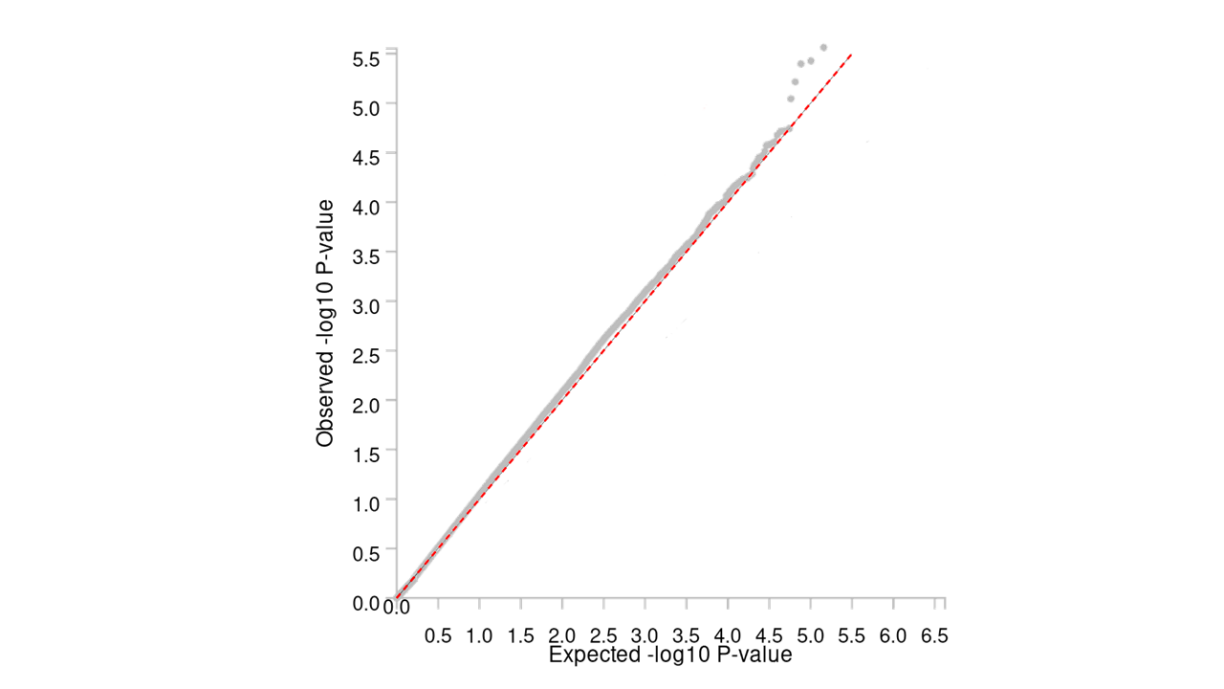 |
| --- | --- |
| A | B |
| 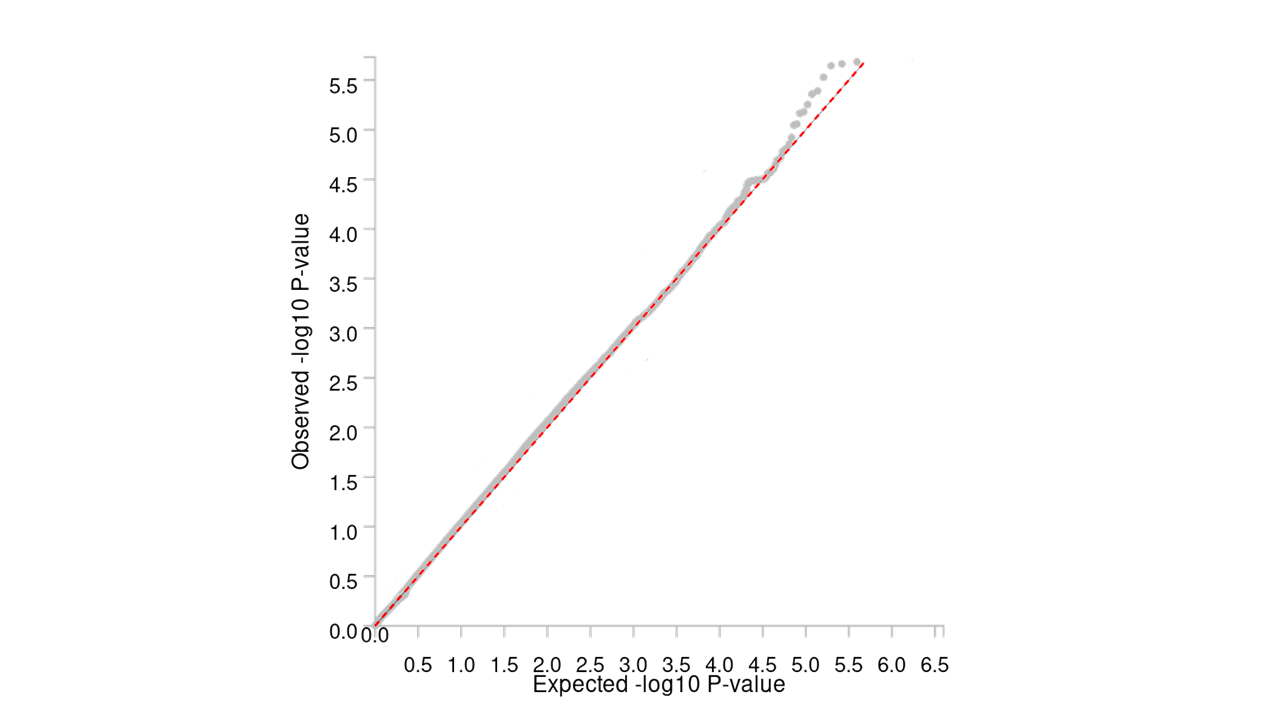 | 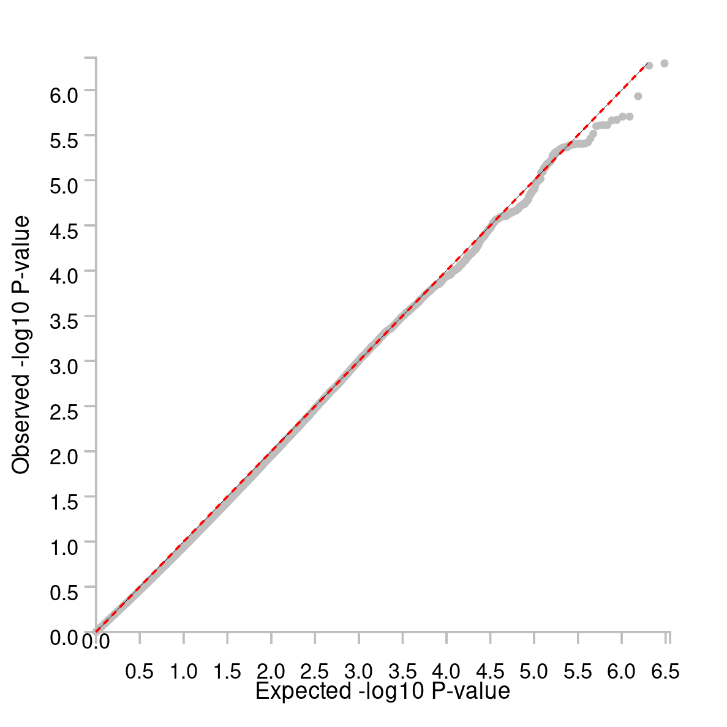 |
| C | D |

Figure 5 QQ plot of GWAS summary statistics for Anterior ILAS. (A) Asian. (B) African American. (C) White. (D) Hispanic.

| 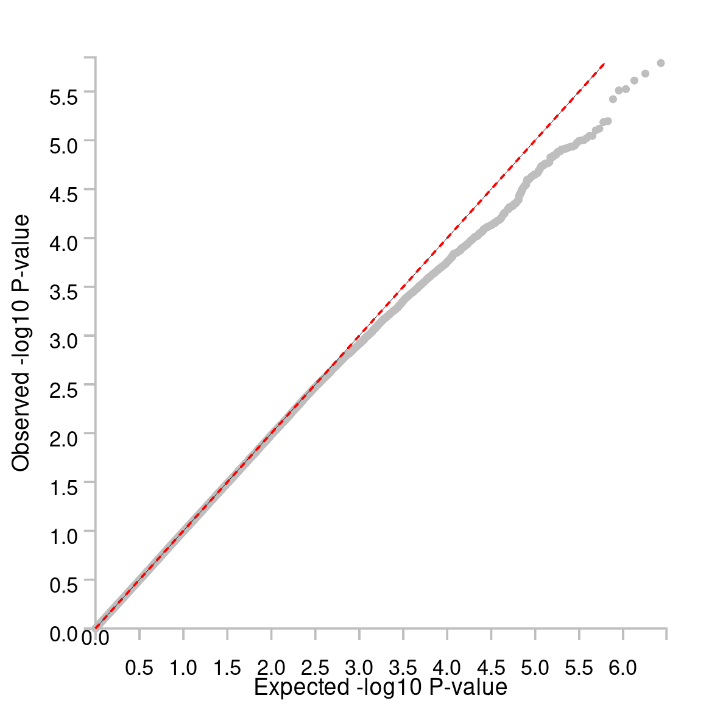 | 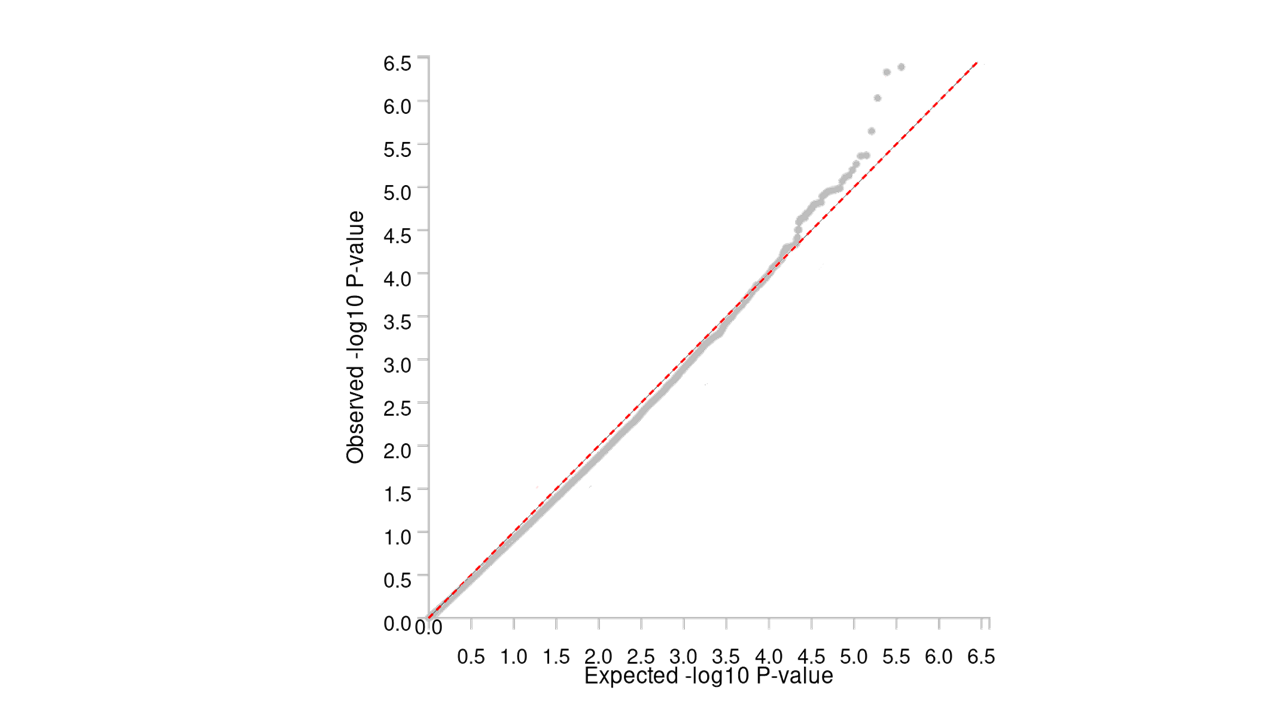 |
| --- | --- |
| A | B |
| 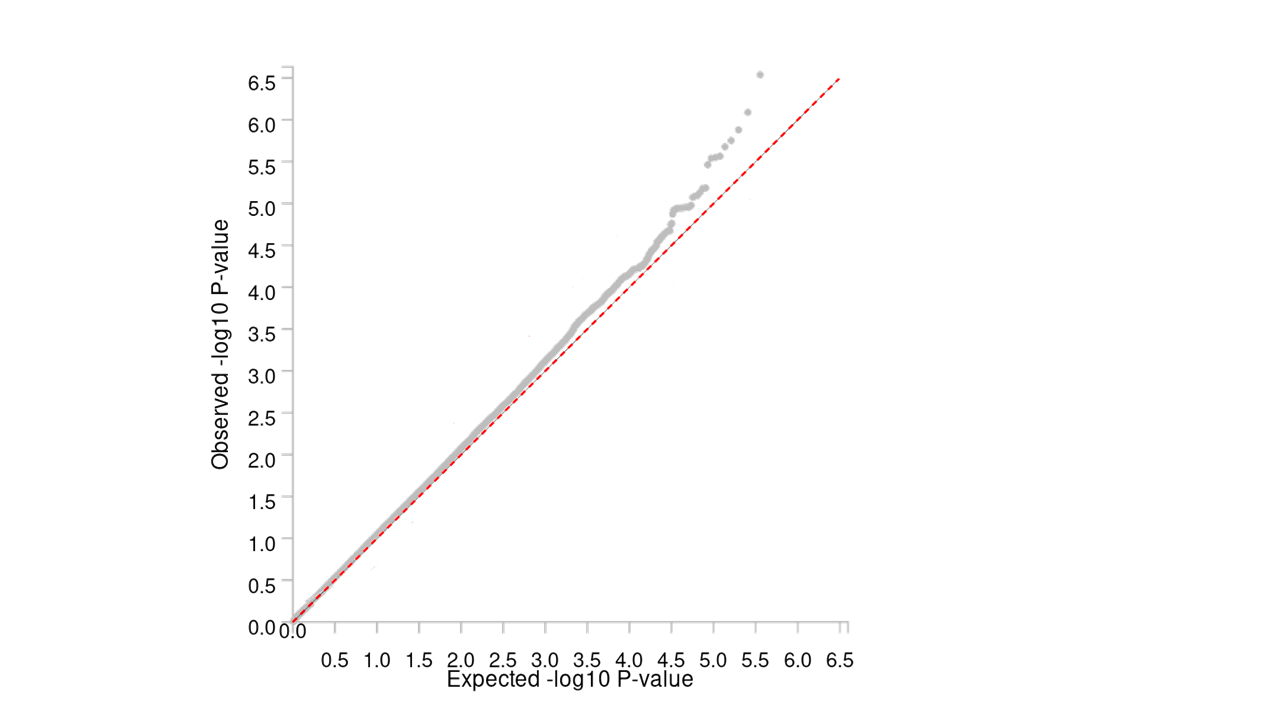 | 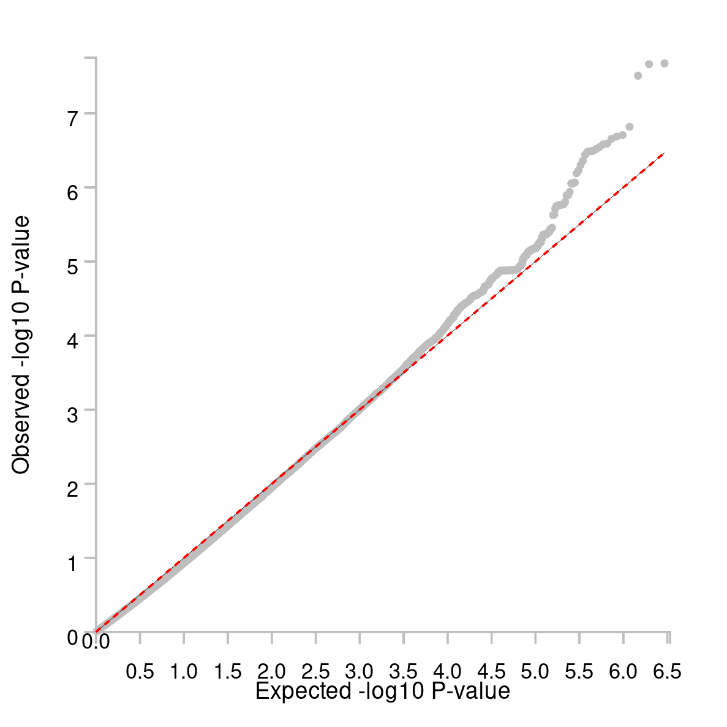 |
| C | D |

Figure 6 QQ plot of GWAS summary statistics for Posterior ILAS. (A) Asian. (B) African American. (C) White. (D) Hispanic.

| 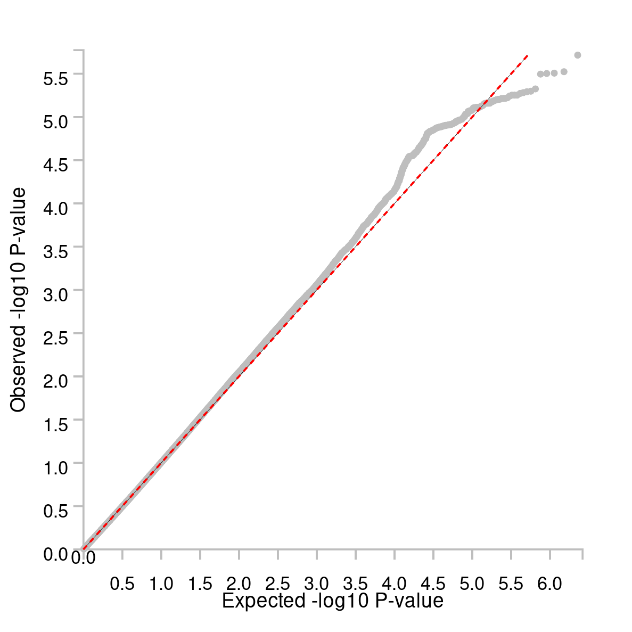 | 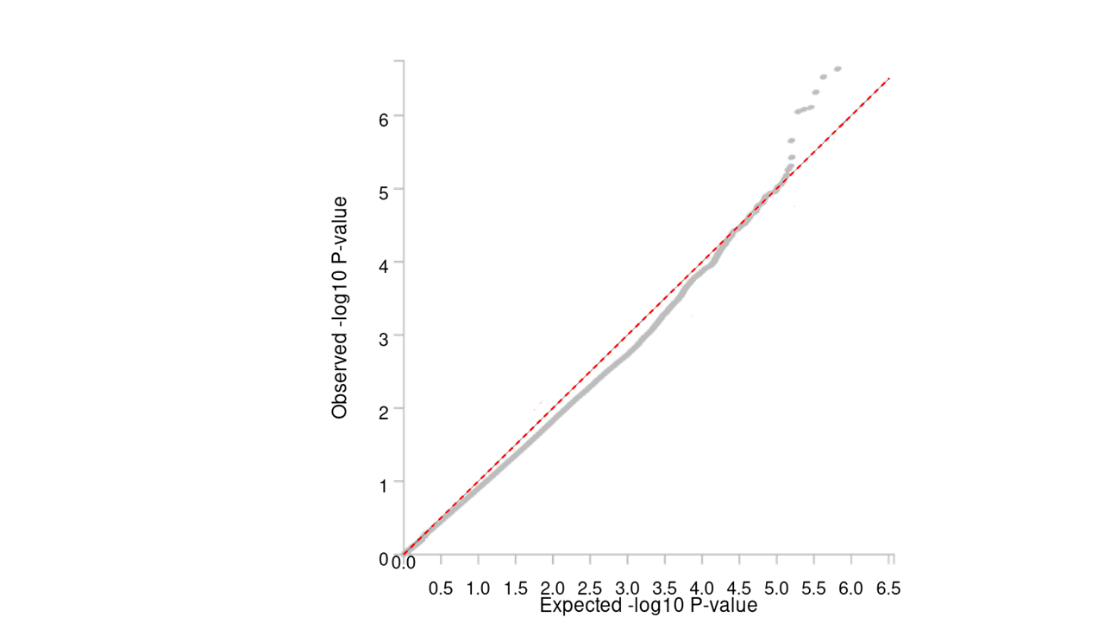 |
| --- | --- |
| A | B |
| 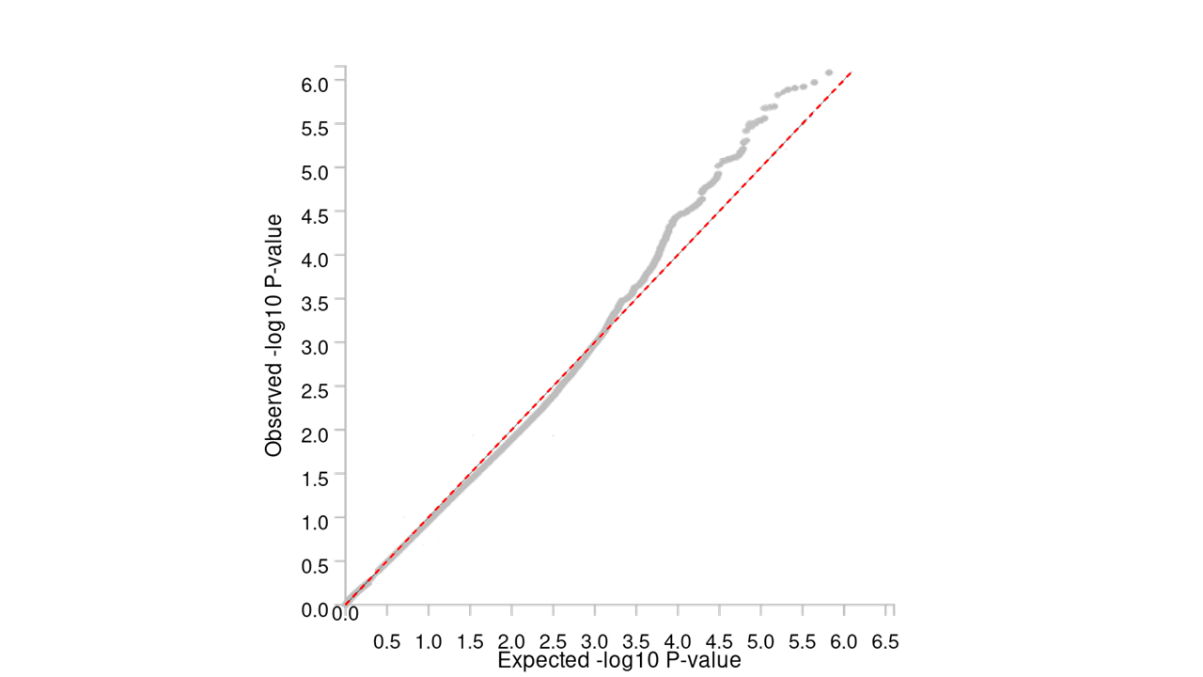 | 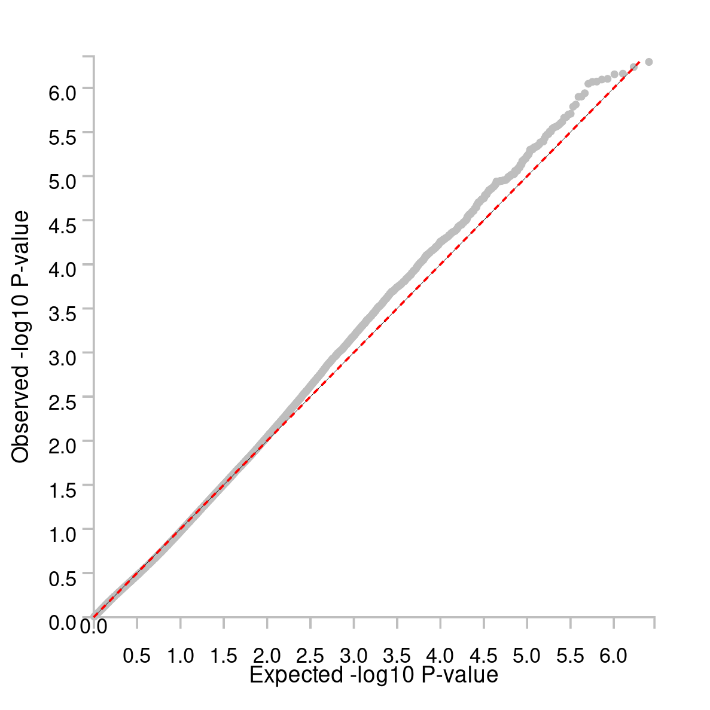 |
| C | D |

Figure 7 Mahattan Plot of gene-based test for Global ILAS. (A) Asian. (B) African American. (C) White. (D) Hispanic.

| A | 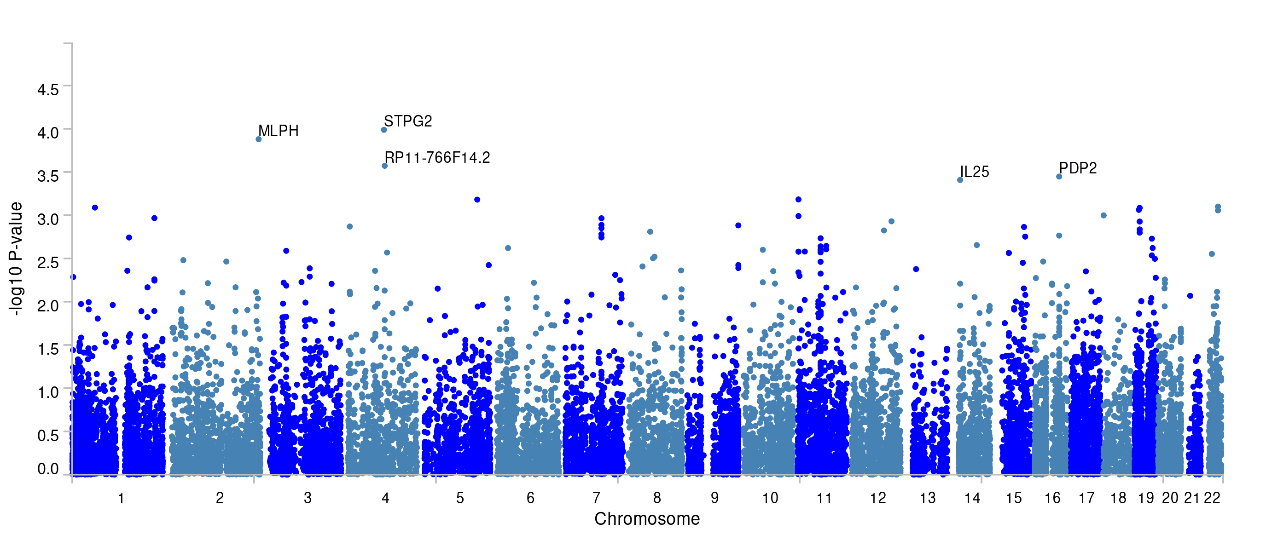 |
| --- | --- |
| B | 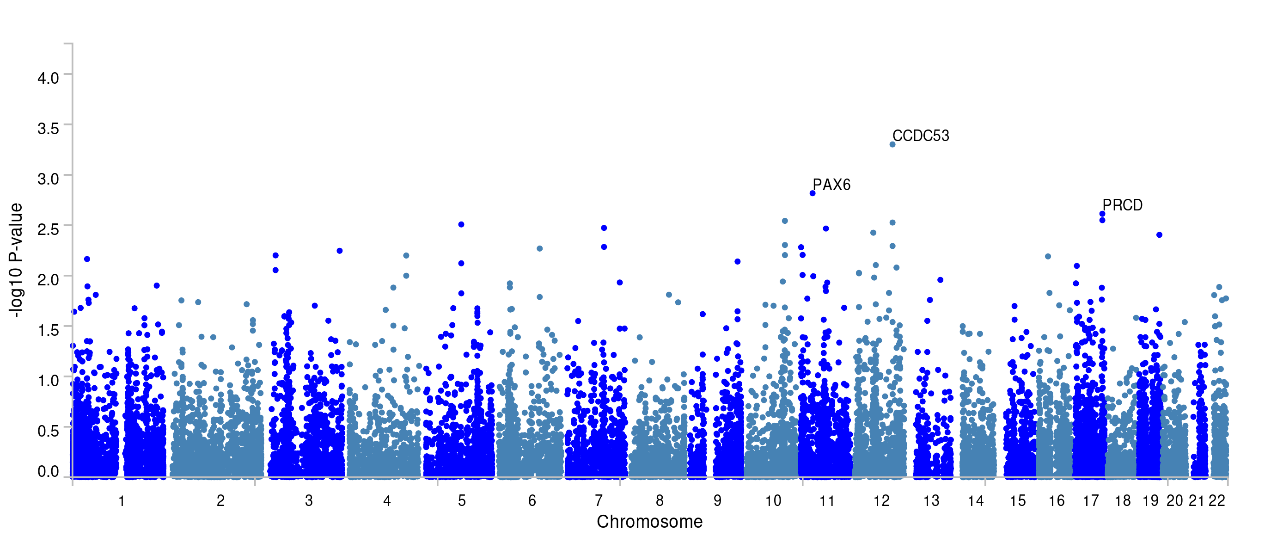 |
| C | 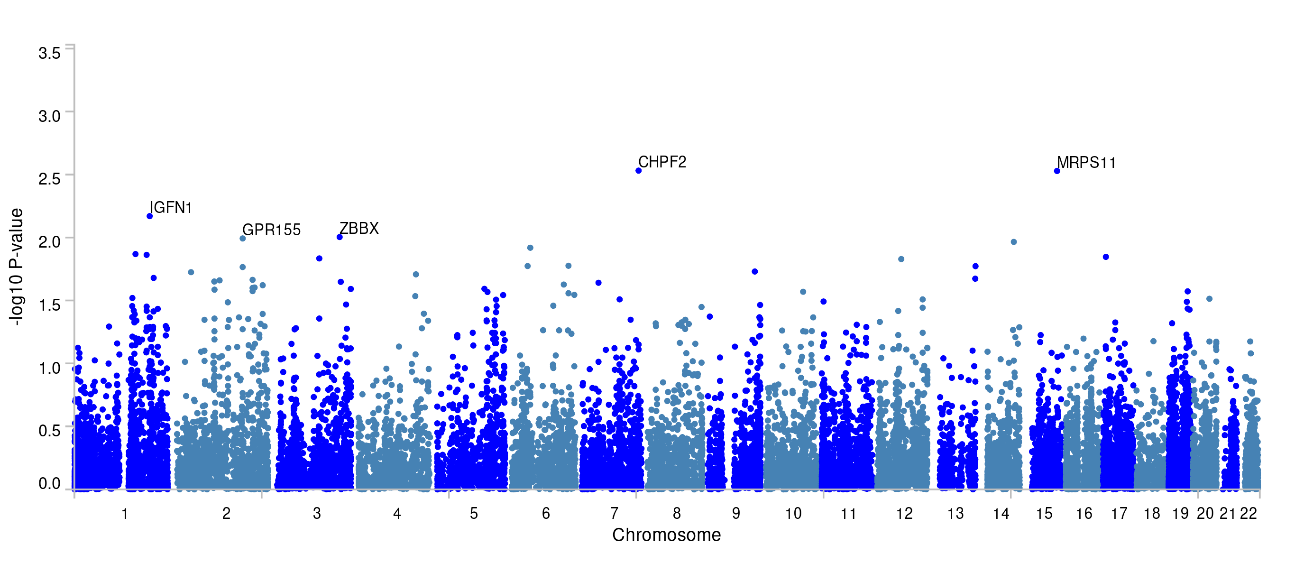 |
| D | 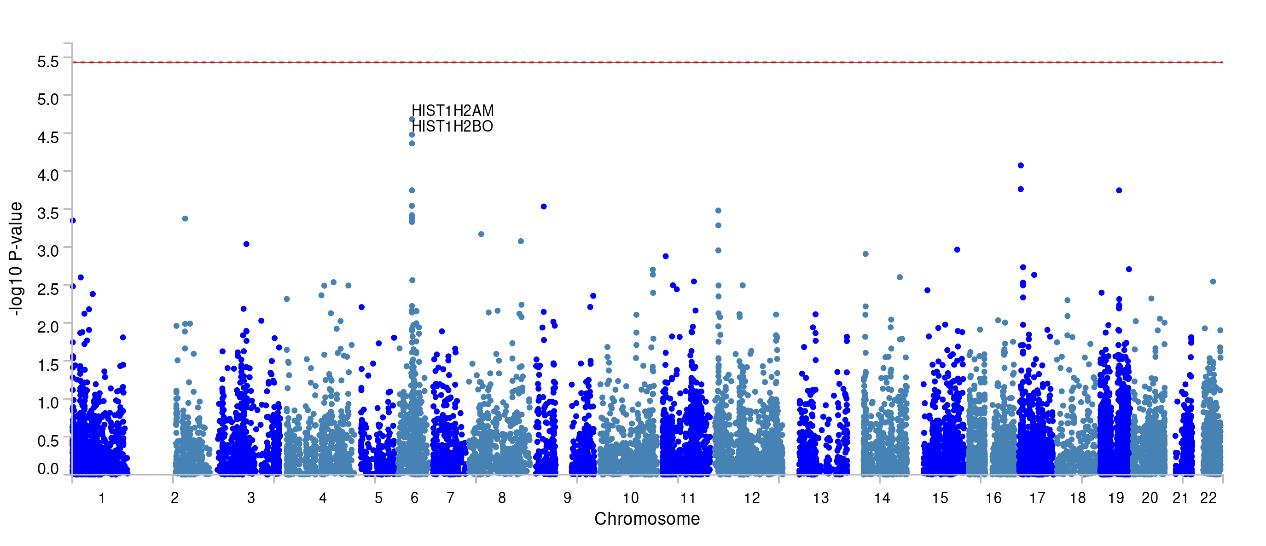 |

Figure 8 Mahattan Plot of gene-based test for Anterior ILAS. (A) Asian. (B) African American. (C) White. (D) Hispanic.

| A | 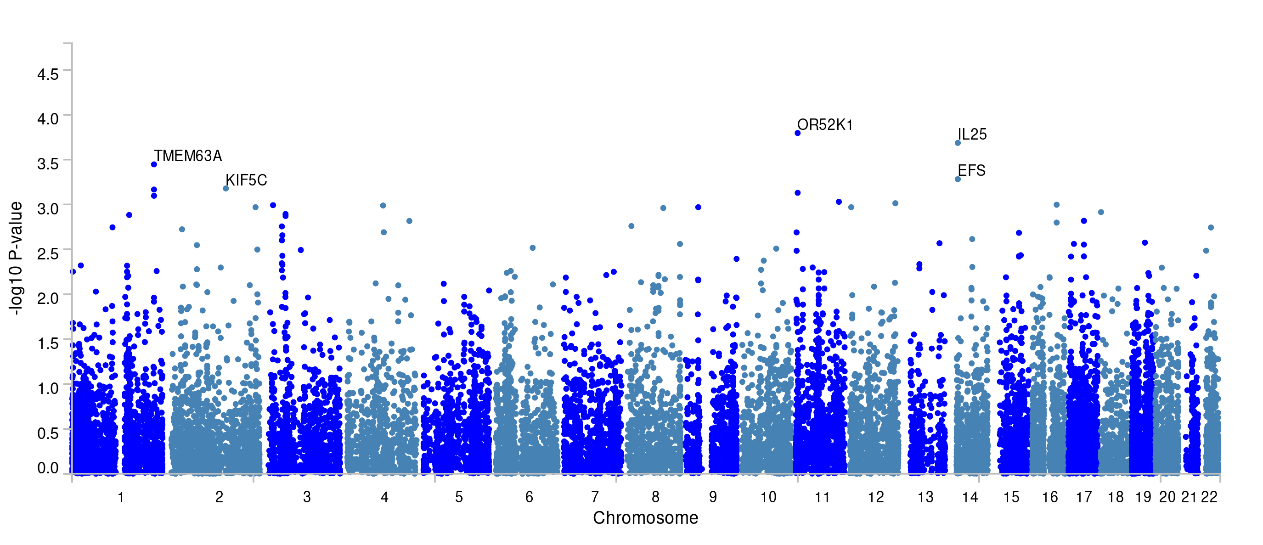 |
| --- | --- |
| B | 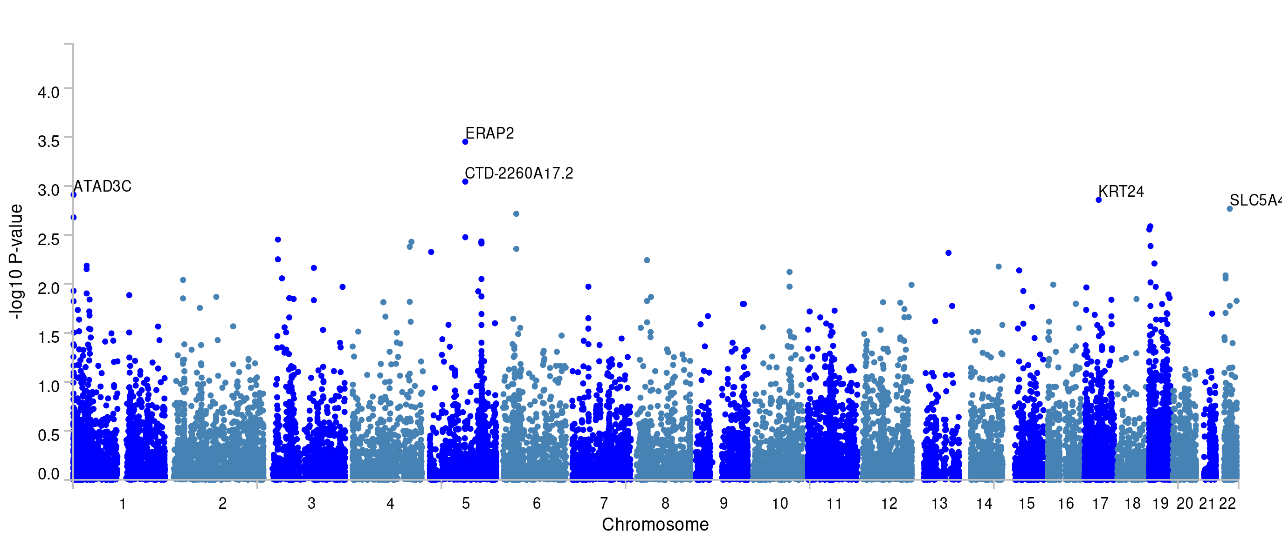 |
| C | 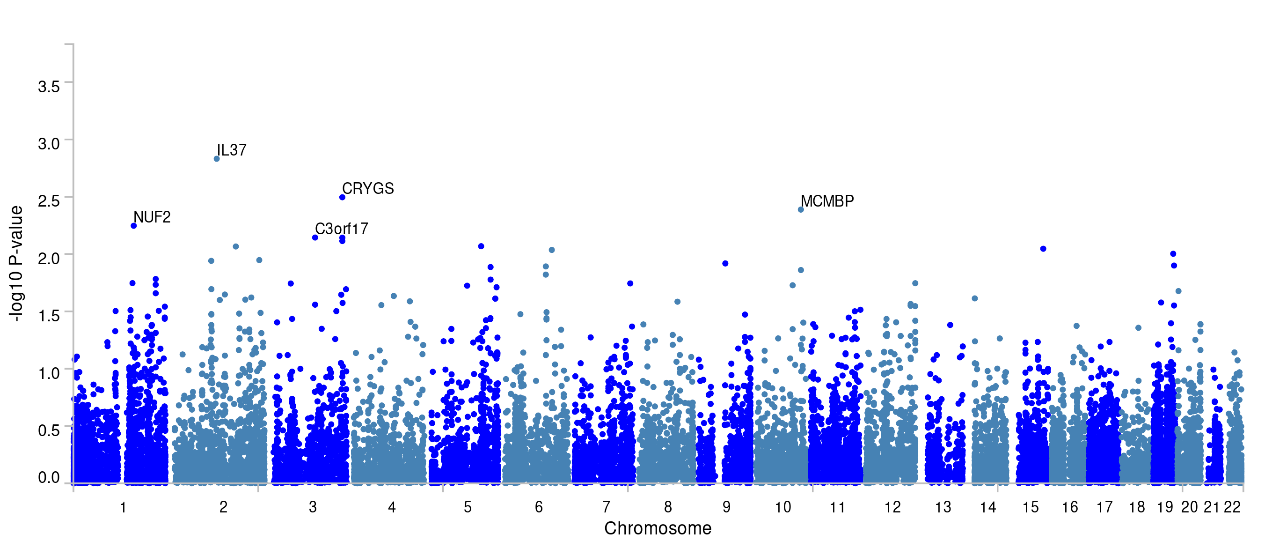 |
| D | 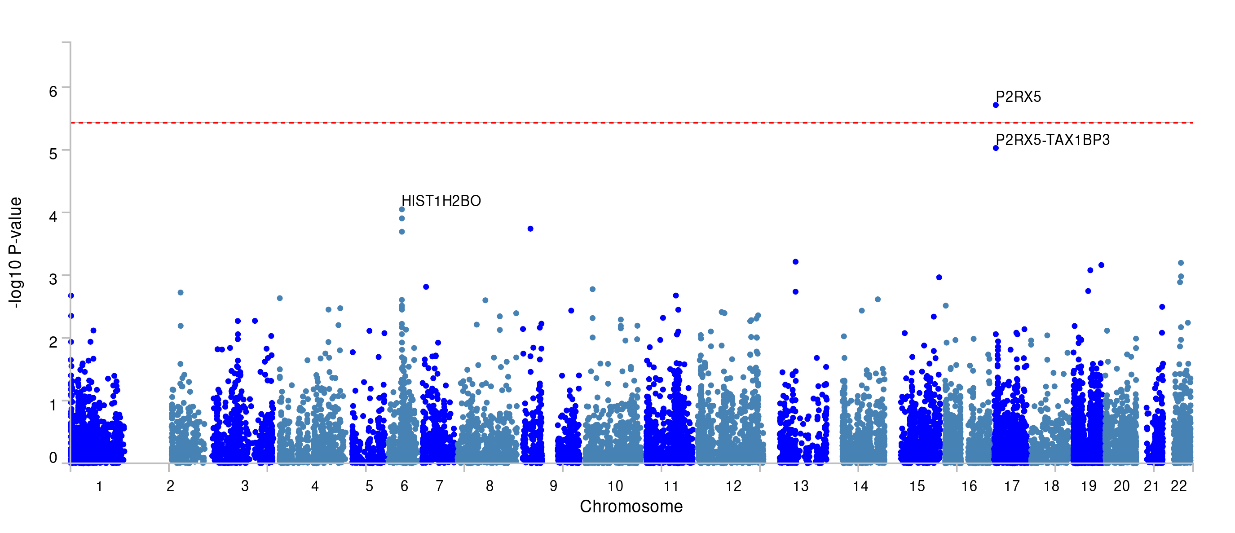 |

Figure 9 Mahattan Plot of gene-based test for Posterior ILAS. (A) Asian. (B) African American. (C) White. (D) Hispanic.

| A | 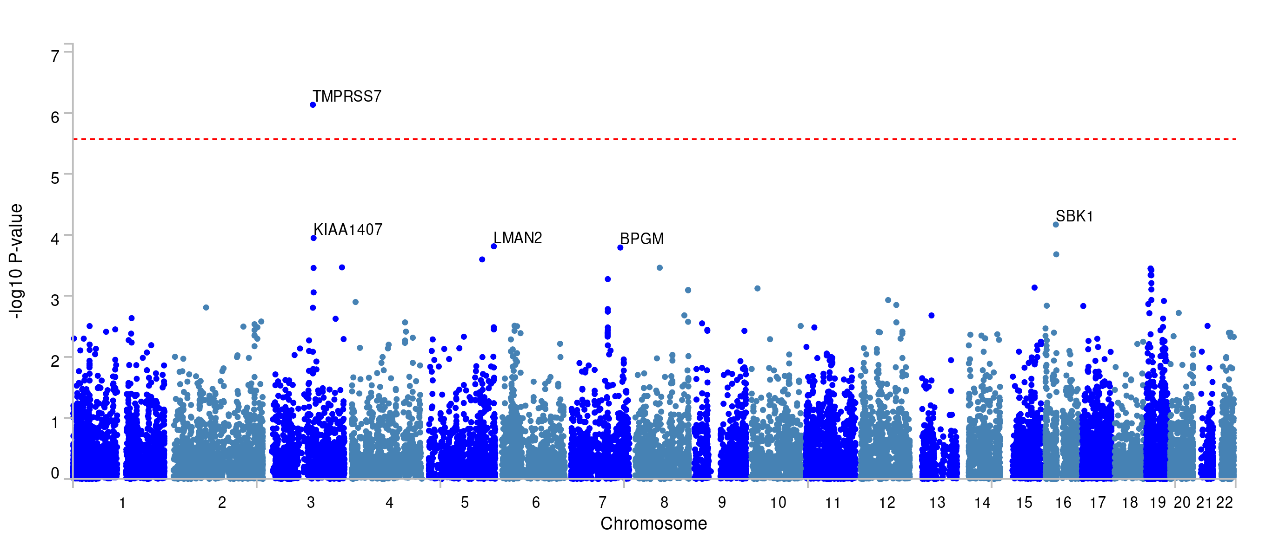 |
| --- | --- |
| B | 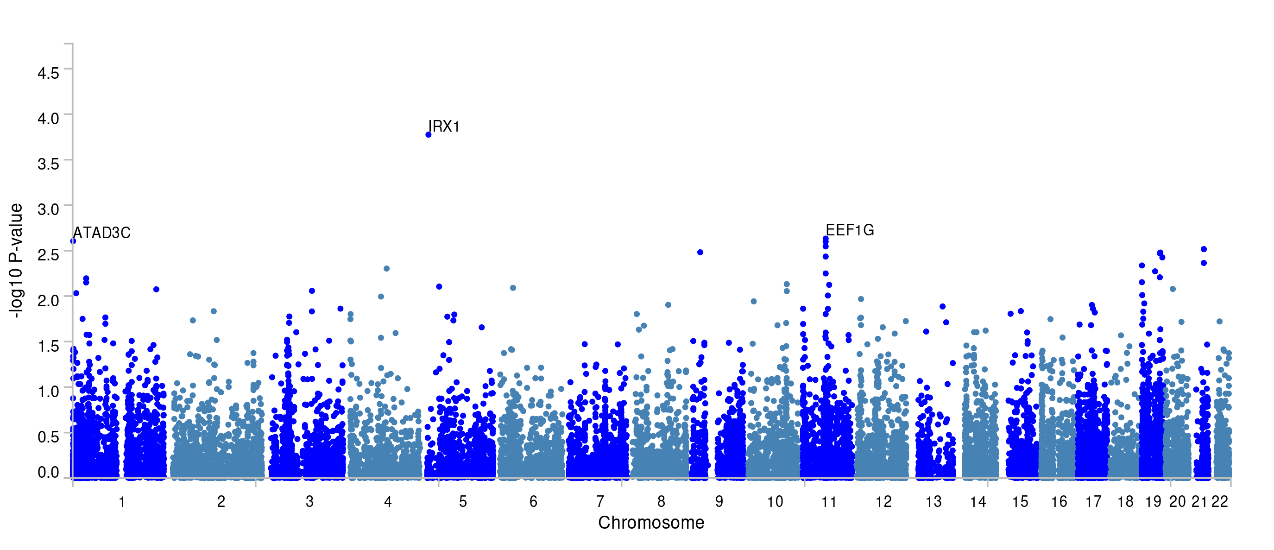 |
| C | 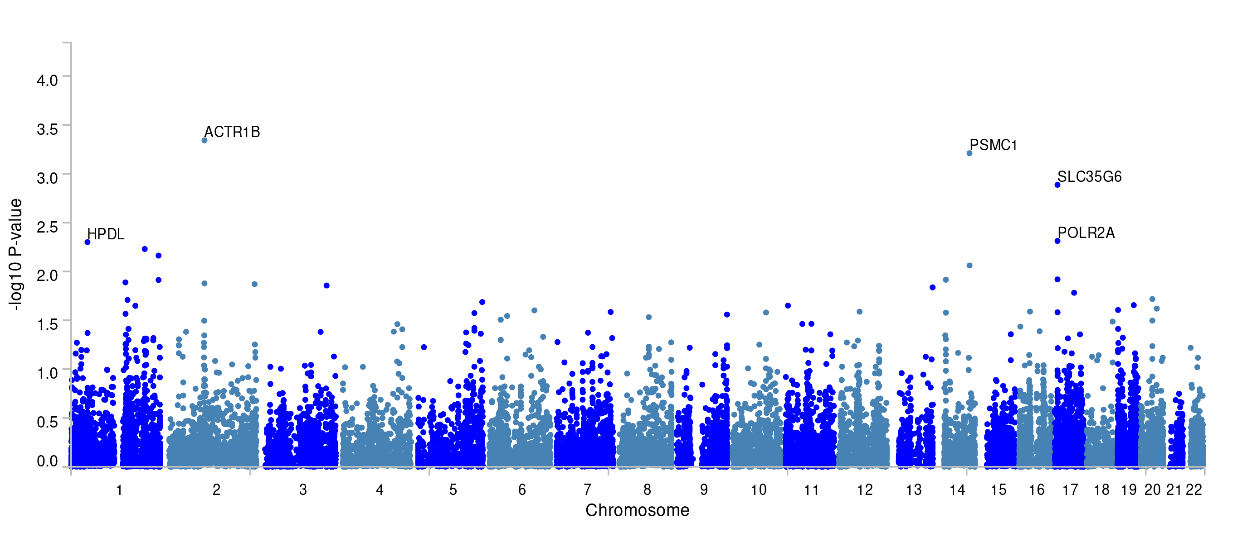 |
| D | 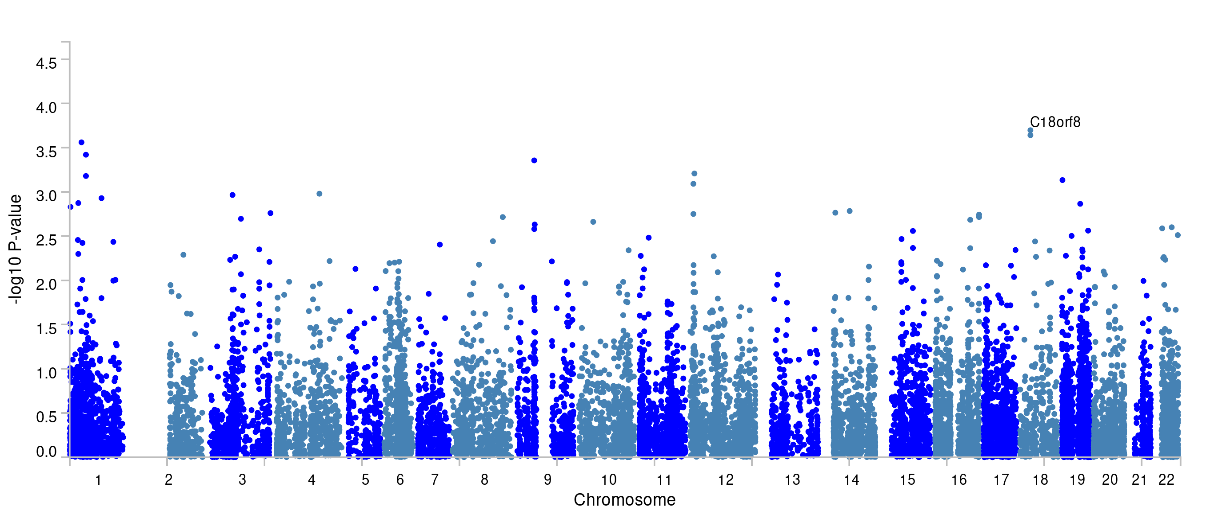 |

Figure 10 Regional plot. (A) rs1528343 (18:8464372) for Hispanic Anterior ILAS.

(B) rs73856305 (3: 111781467) for Asian Posterior ILAS.

| A | 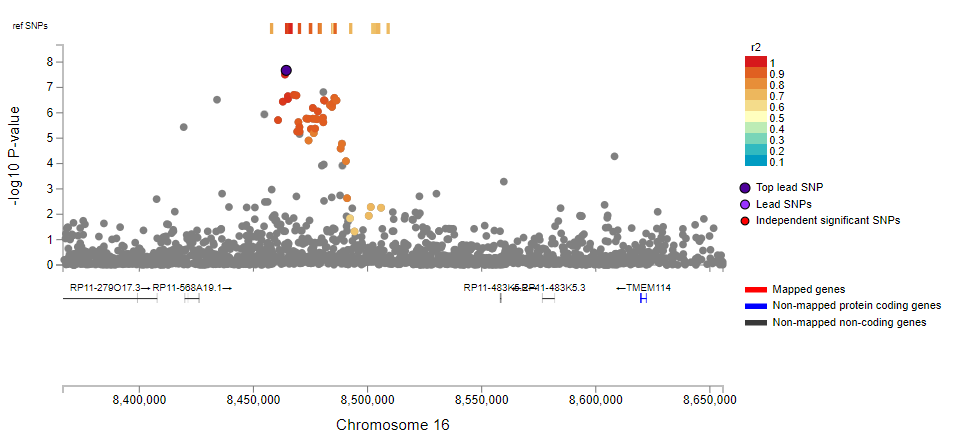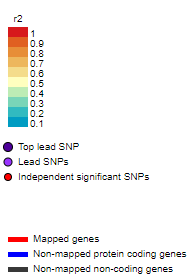 |
| --- | --- |
| B | 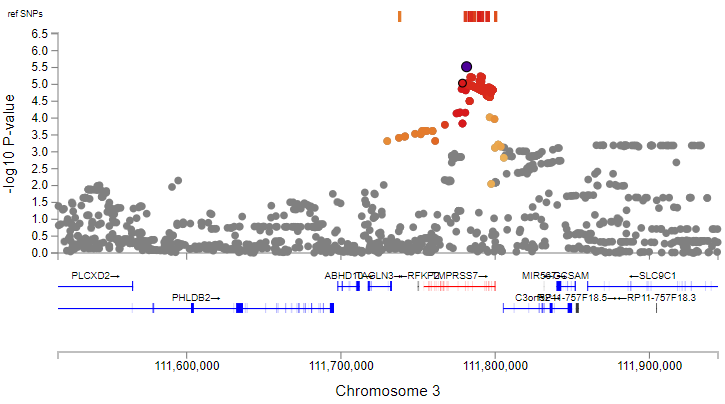 |
